# NaviSE: superenhancer navigator integrating epigenomics signal algebra

**DOI:** 10.1186/s12859-017-1698-5

**Published:** 2017-06-06

**Authors:** Alex M. Ascensión, Mikel Arrospide-Elgarresta, Ander Izeta, Marcos J. Araúzo-Bravo

**Affiliations:** 1 Computational Biology and Systems Biomedicine, Biodonostia Health Research Institute, San Sebastián, 20014 Spain; 2 Tissue Engineering Laboratory, Bioengineering Area, Biodonostia Health Research Institute, San Sebastián, 20014 Spain; 3 Department of Biochemistry and Molecular Biology, University of the Basque Country, Leioa, 48940 Spain; 4 IKERBASQUE, Basque Foundation for Science, Bilbao, 48013 Spain

**Keywords:** Superenhancers, Next-generation sequencing, Parallel computing, Epigenomics, Computational biology, Graphics user interface, Signal algebra

## Abstract

**Background:**

Superenhancers are crucial structural genomic elements determining cell fate, and they are also involved in the determination of several diseases, such as cancer or neurodegeneration. Although there are pipelines which use independent pieces of software to predict the presence of superenhancers from genome-wide chromatin marks or DNA-interaction protein binding sites, there is not yet an integrated software tool that processes automatically algebra combinations of raw data sequencing into a comprehensive final annotated report of predicted superenhancers.

**Results:**

We have developed NaviSE, a user-friendly streamlined tool which performs a fully-automated parallel processing of genome-wide epigenomics data from sequencing files into a final report, built with a comprehensive set of annotated files that are navigated through a graphic user interface dynamically generated by NaviSE. NaviSE also implements an ‘epigenomics signal algebra’ that allows the combination of multiple activation and repression epigenomics signals. NaviSE provides an interactive chromosomal landscaping of the locations of superenhancers, which can be navigated to obtain annotated information about superenhancer signal profile, associated genes, gene ontology enrichment analysis, motifs of transcription factor binding sites enriched in superenhancers, graphs of the metrics evaluating the superenhancers quality, protein-protein interaction networks and enriched metabolic pathways among other features. We have parallelised the most time-consuming tasks achieving a reduction up to 30% for a 15 CPUs machine. We have optimized the default parameters of NaviSE to facilitate its use. NaviSE allows different entry levels of data processing, from sra-fastq files to bed files; and unifies the processing of multiple replicates. NaviSE outperforms the more time-consuming processes required in a non-integrated pipeline. Alongside its high performance, NaviSE is able to provide biological insights, predicting cell type specific markers, such as *SOX2* and *ZIC3* in embryonic stem cells, *CDK5R1* and *REST* in neurons and *CD86* and *TLR2* in monocytes.

**Conclusions:**

NaviSE is a user-friendly streamlined solution for superenhancer analysis, annotation and navigation, requiring only basic computer and next generation sequencing knowledge. NaviSE binaries and documentation are available at: https://sourceforge.net/projects/navise-superenhancer/.

**Electronic supplementary material:**

The online version of this article (doi:10.1186/s12859-017-1698-5) contains supplementary material, which is available to authorized users.

## Background

Superenhancers (SEs) are a novel class of transcription regulatory DNA regions with unusually strong enrichment for binding of transcriptional coactivators such as Mediator of RNA polymerase II transcription subunit 1 (MED1), activation histone marks such as H3K27ac, or cell and tissue-specific transcription factors (TFs) [1]. As a result, SEs represent large clusters of transcriptional enhancers that drive the expression of ‘master control’ genes that define cell identity. SEs differ from typical enhancers (TEs) for enclosing higher TF binding density and number of TF binding sites (TFBSs), which correlate with a much higher expression of their target genes [2]. Since SEs determine cell fate and gene expression regulation [3], they are related to altered expression of genes contributing to diseases such as Alzheimer or systemic lupus erythematosus [4]. Aberrant DNA methylation patterns in SEs, as well as SE-associated gene sets, have also been found to be altered in cancer [5–7].

Although protocols for computational prediction of SEs already exist [4], there is yet no tool that integrates all the processing stages from the raw data reads generated by the sequencer, through quality control and reads alignment, to peak estimation and peak stitching, ending with a fully annotated and interactive documentation of the results.

Furthermore, although SEs were initially predicted with MED1 [4] and activation histone marks such as H3K27ac, which has been proposed as a proxy for their estimation [2], the combination of several activation and repression epigenomics marks could help sharpen SE predictions. Therefore, we have designed NaviSE to use data with a wide range of chromatin status information, being able to process raw data from Assay for Transposase Accessible Chromatin (ATAC-seq) and DNase I hypersensitive sites (DHSs) experiments, apart from the usual ChIP-seq signals. In the case of other signals such as DNA methylation, NaviSE is prepared to integrate their information to perform SE predictions with the only condition that the user provides such data in bed or bam files, such as the bam files produced by the Parallel Processing Pipeline software for automatic analysis of Bisulfite Sequencing data (P3BSseq) [8].

On the other hand, there are no computational tools neither integrating several epigenomics signals simultaneously, nor performing signal algebra. Moreover, CPU resources and running-time are crucial for the high quantity of data produced by Next Generation Sequencing (NGS) technologies, hence another of the main demands in NGS software development is the parallelisation of the most time-consuming processes.

To meet all these demands, we have developed NaviSE, a user-friendly tool which automatically processes and integrates multiple genome-wide NGS epigenomics signals from various input file formats into an interactive HTML report, built with annotations about SEs, such as associated genes, gene ontology (GO), graphs with metrics and statistical analysis, integrating all the data into the Graphical User Interface (GUI) to navigate through all the results. NaviSE parallelises the most relevant and time-consuming processes to optimise them, running multiple analysis in a significantly reduced amount of time. Finally, NaviSE is developed for users with working knowledge in informatics.

## Implementation

### Preprocessing of NGS files

Before the determination of SEs, NaviSE prepares the raw data, allowing multiple replicates and controls at once. The main steps for such preprocessing are as follows:

1. *Input format file recognition and file processing*: NaviSE recognizes multiple file formats, e.g.,.sra,.fastq,.sam,.bam and.bed, and transforms an *upstream* format (.sra,.fastq,.sam) into a.bam file. In the absence of *upstream* files, *downstream*.bed files are also processed to.bam files.
2. *Alignments*: Performed by default with bowtie2 [9],.sam files are processed to.bam files by samtools. NaviSE also allows read alignment with MOSAIK [10], STAR [11] and BWA [12] aligners. Furthermore, users may generate their own.sam or.bam files with other aligners, and NaviSE will recognize these files for further processing.
3. *Quality control with FastQC*: NaviSE performs the quality analysis of the reads from the.fastq files using FastQC to create a report including several quality parameters, such as per base quality, GC content, *k*-mers distribution or presence of adapters.
4. *Combination of replicates and peak calling with MACS*: If there is more than one replicate or control, NaviSE will combine all the associated.bam files into one, and calculate the signal peaks with MACS (Model-based Analysis for ChIP-Seq) [13]. If control files are introduced for background correction, NaviSE configures MACS to use the control signal to calculate the peaks from the sample. Conversely, if no control is introduced, NaviSE configures MACS to use a pre-calculated background.

### SE prediction and annotation

Once the data is preprocessed, a SE prediction and ranking is performed. SEs then are further analysed in search of SE related genes, DNA sequence motifs, GO terms or statistical estimations.

1. *Epigenomics signal algebra*: In case more than one epigenomic signal was used to predict SEs, NaviSE integrates all the signals to improve the SE prediction. The way in which different epigenomic signals are combined is defined by the names of the signal data files *S* *i* *g*∈ {H3K27ac, H3K4me1, H3K4me3, H3K9me3, H3K27me3, ATAC-seq, DHS,…} separated by signal operators *O* *p* *e*∈ {AND, OR, NOT, XOR, +, - SYM}.
  The way these algebra operators have been adapted to operate over pairs of genomic signals is illustrated in Fig. 1. To invoke this algebra, NaviSE is called writing these signal and operators as additional arguments in the command line:
  $$Sig_{1}\ Ope_{1,2}\ Sig_{2}\ Ope_{2,3}\ Sig_{3}\ Ope_{3,4}\ Sig_{4} $$
  where *S* *i* *g*_*i*_ is the name of the file containing the epigenomics data of a type of signal *i*, and *O* *p* *e*_*i*,*i*+1_ is the pairwise signal operator applied to combine *i* and *i*+1 signals.

**Fig. 1:**
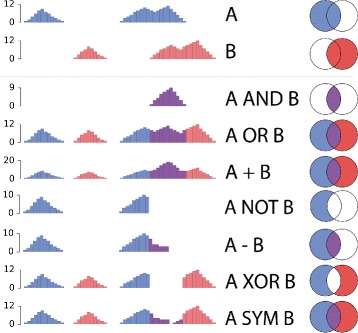
Epigenomics signal algebra. Schemes of the different pairwise operations implemented in NaviSE. The top two rows depict an example of the two epigenomic signals to be combined, and the remaining rows illustrate the signal profile after applying the respective operator. Simplified Euler-Venn diagrams given in the rightmost column illustrate the set operations. AND, OR, NOT and XOR are Boolean operations which do not change the signal pileup; whereas +, -, and SYM are arithmetic operations which can change the signal pileup. In the case of - and SYM, negative pileups are transformed into zeros
  When performing ‘epigenomics signal algebra’, NaviSE picks the first pair of signals separated by each operator starting from the left (*S* *i* *g*_1_ *O* *p* *e*_1,2_ *S* *i* *g*_2_). Once an operation is processed, its results are combined with the next signal using the next operator ((*S* *i* *g*_1_ *O* *p* *e*_1,2_ *S* *i* *g*_2_) *O* *p* *e*_2,3_ *S* *i* *g*_3_). This process continues from left to right side recursively until the last signal identifier is reached. To speed up the process, for each pair of signals NaviSE searches first all their overlapping regions and performs the signal operator only over these regions.
2. *SE prediction*: To predict the SEs in a sample, NaviSE performs the *stitching* of MACS peaks which fall within a threshold distance, using our own implementation of the algorithm developed by Young’s lab [2], in which MACS peaks (inferred as enhancers) are stitched according to a constant distance (12.5 kb by default) criterion algorithm, in case the distance between the end of one MACS peak and the start of the following peak is less than the established threshold, they are *stitched* as a single peak. Then, NaviSE ranks the *stitched* peaks with a score based on the measured signal level within the *stitched* region.
  NaviSE assigns a score to each *stitched enhancer*, considering that a *stitched enhancer* with a higher number of bam reads has a higher SE predictive value. Thus, to build the SE ranking, NaviSE takes the raw reads from the.bam files, and for each *stitched enhancer* it collects all the reads over the *stitched enhancer* support. This support is defined by the DNA sequence lying between the *stitched enhancer* start, *S* *T* *I* *T*_*start*_, and the *stitched enhancer* end, *S* *T* *I* *T*_*end*_, nucleotide positions. Then, we define the *stitched enhancer* count, *C* *o* *u* *n* *t*_*STIT*_, as the cumulative sum of the bam reads throughout the *stitched enhancer* support:
  1 $$ Count_{STIT}= \sum\limits_{i=1}^{N_{reads}}\sum\limits_{j=STIT_{start}}^{STIT_{end}} read_{i}(j)   $$
  where *r* *e* *a* *d*_*i*_(*j*) indicates whether a bam read *i*, from the set of *N*_*reads*_, lies at the position *j* of the *stitched enhancer* within the support [ *S* *T* *I* *T*_*start*_, *S* *T* *I* *T*_*end*_]. Therefore, *r* *e* *a* *d*_*i*_(*j*)=1 if a nucleotide of the bam read *i* is mapped to the location *j* of the *stitched enhancer*, and *r* *e* *a* *d*_*i*_(*j*)=0 otherwise.
  Then, the SE ranking, *r*, is defined as the sorted list of *C* *o* *u* *n* *t*_*STIT*_ in descending order:
  2 $$ r=\text{sort}_{\blacktriangledown} \{Count_{STIT}\}   $$
  thus, Eq. 2 assigns position one in the ranking to the *stitched enhancer* with the highest *C* *o* *u* *n* *t*_*STIT*_, position two in the ranking to the *stitched enhancer* with second highest *C* *o* *u* *n* *t*_*STIT*_, etc. until we reach the *stitched enhancer* with the lowest *C* *o* *u* *n* *t*_*STIT*_.
  The next step is the determination of the SE threshold (*θ*_*SE*_), the position of the ranking for which the *stitched enhancers* whose rank is below *θ*_*SE*_ will be considered as SEs, and TEs otherwise. To determine *θ*_*SE*_, we scale both *C* *o* *u* *n* *t*_*STIT*_ and *r* between 0 and 1. Then, we determine *θ*_*SE*_ as the position of *r* whose slope is nearest to 45°.
3. *SE gene assignment*: Once the SE locations are determined, each SE is assigned a gene by proximity with the closest transcription start site (TSS). NaviSE also includes information about genes overlapping the SE or genes proximal to each SE.
4. *Subpeak annotation*: The SEs and TEs subpeaks have been shown to act synergistically within the SE despite being individual and independent structures [14]. To provide detailed information about the SE subpeaks structure and location, NaviSE performs a structural annotation of the subpeaks that represent each SE. The annotation contains the following parameters:
  Number of subpeaks, *loci* and TSS locations.
  Association to TSSs: Due to the TSS specific regulation role, a SE inside a TSS might not exert the role of SE itself. Thus, to understand the regulatory role of the SEs, it is important to resolve their association to TSSs. This analysis is portrayed by two related values: (i) the *Percentage OUTS*, which is the percentage of subpeaks outside the range of the user-defined distance within the TSS, and (ii) the *Enhancer Type*, a classification of the SE according to *Percentage OUTS*. The categories assigned to *Enhancer Type* are labeled as *Pure* if all the subpeaks are outside the TSS, *Only TSS* if all the subpeaks lay within the TSS, and *Mixed* if there are both types of subpeaks.
5. *Automatic generation of SE peak distribution profiles*: To visualize the SE peak distribution we have implemented in NaviSE our own Genome Viewer Tool (GVT). With this tool, two snapshots at *near* and *far* distances for each SE are portrayed, which are shown in the *SE table* window of the final report. NaviSE dynamically calculates the optimal range for each snapshot, based on the width of the SE. With *near* shot the user is able to determine the morphology of the SE, and with *far* the user is able to locate the SE in its genomics surroundings. In each snapshot both the location of the SE and the enhancer peaks determined by MACS are shown.
6. *HOMER motif finding*: SEs enclose high number of TFBSs [2]. Therefore, identifying such TFBSs is important for SE annotation. To find motifs of regulatory elements (mainly TFs) that are specifically enriched in the *loci* of SEs, relative to the *loci* of TEs, NaviSE uses the Hypergeometric Optimization of Motif Enrichment (HOMER). As a result, NaviSE generates in the final report a *HOMER table*, which includes motifs enriched in SEs, and a list of *de novo* motifs for which their respective binding elements are predicted by HOMER.
7. *Gene Ontology Enrichment Analysis (GOEA)*: To predict the functionality of the SEs, based on the closest gene of each SE determined by HOMER, NaviSE uses goatools [15].
8. *Pathways and protein-protein interaction annotation*: To obtain annotation of TFs and pathways related to SEs, NaviSE uses Enrichr [16]. To obtain protein-protein interaction (PPI) networks of SEs, NaviSE uses the database of PPIs String [17]. Results from Enrichr and String are processed and integrated into the final report to be navigated through NaviSE GUI for an easier interpretation for the user.
9. *NaviSE GUI*: To navigate throughout all the results, we have implemented an interactive chromosomal plot (Fig. 2) that represents the SE location in a karyotype; alongside with graphs that depict statistical values and properties related to SEs (shown in “Results” section), as well as information related to GOEA or Enrichr.

**Fig. 2:**
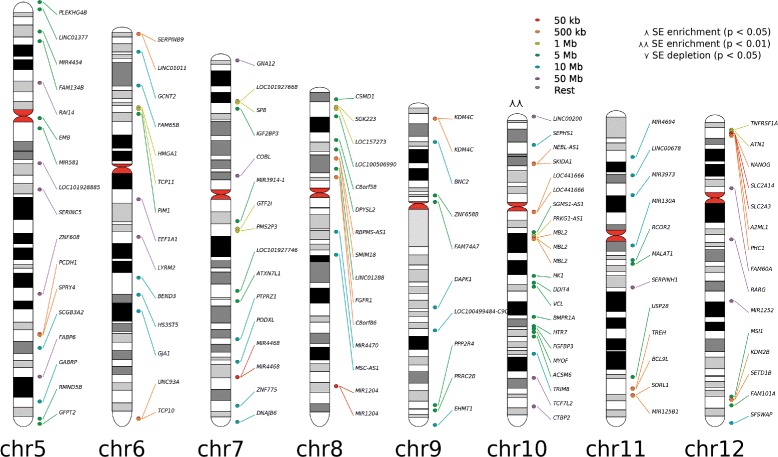
Chromosomal plot. Partial snapshot of the chromosomal plot of SEs predicted with H3K27ac histone mark in ESCs. *Hot-spots* and *line colours* represent distances between two SEs (*red* to *violet* represents smaller to bigger distances), and $\curlywedge /\curlywedge \curlywedge $ and $\curlyvee /\curlyvee \curlyvee $ represent chromosome enrichment or depletion in genes with statistical significance *p* of 0.05 and 0.01, respectively
  Chromosomal plots are designed to include *hot-spots* with links to the elements of the *SE table* from the final report, which are activated when the user navigates with the mouse over the gene names on the chromosomal plot. To enhance the usability of this feature, NaviSE generates three types of chromosomal plots:
  Enrichment plot: it shows the *loci* location and the chromosome enrichment or depletion.
  Rank plot: it shows *loci* coloured according to their rank in the *SE Table*. Several percentiles are represented based on the rank of the SE, and SEs falling within a percentile will be coloured correspondingly.
  Closeness plot: it represents the proximity between SEs, according to which SEs will be coloured. This plot is highly useful to discern clusters of SEs that look overlapped. For the ordered list {*S* *E*_1_,*S* *E*_2_,⋯,*S* *E*_*a*−1_,*S* *E*_*a*_,*S* *E*_*a*+1_,⋯,*S* *E*_*c*−1_,*S* *E*_*c*_}, of *c* SEs within a chromosome, for which each *S* *E*_*k*_ support is defined by its start (*S* *E*_*k*,*start*_) and end (*S* *E*_*k*,*end*_) *loci* positions, the closeness of a *S* *E*_*k*_ is its distance *C*(*S* *E*_*k*_) to the closest SE, determined by the following expression:
    3 $$ {}C(SE_{k}) = \left\{\begin{array}{ll} SE_{2,start}-SE_{1,end} & \text{if}\ k=1\\ SE_{c,start}-SE_{{c-1},end} & \text{if}\ k=c\\ \min(SE_{{a+1},start}-SE_{a,end},\\ E_{a,start}-SE_{{a-1},end}) & \text{otherwise}\\ \end{array}\right.  $$
  In all these chromosomal plots a probability *p* determining whether a chromosome is enriched (marked with $\curlywedge $ for *p*<0.05 and $\curlywedge \curlywedge $ for *p*<0.01) or depleted (marked with $\curlyvee $ for *p*<0.05 and $\curlyvee \curlyvee $ for *p*<0.01) with SEs is calculated by a binomial approximation of the hypergeometric distribution ($h(k; K,n,N) \rightarrow b(k; K,z) \;; \;\;z=\frac {n}{N}$) [18], where *N* is the number of genes in the whole genome, *K* is the number of SEs in all chromosomes, *n* is the number of genes in that chromosome, and *k* is the number of SEs in that chromosome.
10. *Gene Set Enrichment Analysis (GSEA)*: To obtain additional functional annotation of SEs, NaviSE performs the GSEA [19] from SE-associated genes, using gene sets from the Molecular Signatures Database (MSigDB).

### Statistics of the comparison between TE and SE

Although both SEs and TEs derive from MACS peaks, they structurally differ for having higher peak density. To illustrate the differences between SEs and TEs, NaviSE shows in the final report a collection of metrics and plots depicting the differences between them. Among the most important plots are:

1. Ranking of SEs by the order of SE score: It is the plot of *C* *o* *u* *n* *t*_*STIT*_, given by Eq. 1 *vs* *r*, given by Eq. 2. It typically shows a *hockey stick* shape, with the inflection point marking the boundary between SEs and TEs, *θ*_*SE*_.
2. INSs and OUTs: It shows statistics about the percentages of SEs and TEs that lay within a TSS or not. This might be interesting if a sample contains an elevated percentage of SEs within TSS, as some of these SEs might be misinterpreted as promoter signals.
3. SE *vs* TE length distribution: It shows the distribution of SE and TE length and pileup in a double histogram and a scatter plot. The histogram lying on the X-axis of the scatter corresponds to the length of SEs and TEs; and the histogram on the Y-axis corresponds to the pileup. This graph is complementary to the ranking of SEs by SE score, to shed light on the population of SEs and TEs.
4. SE *vs* TE subpeak length distribution: This graph contains the same elements than the SE *vs* TE length distribution graph, although showing the distribution of enhancers inferred by MACS.
5. Number of subpeaks (for number of bins *N*=10 and *N*=20): It shows the distribution of subpeaks each SE or TE has.

### Generation of the NaviSE report

The final step of NaviSE is the generation of an HTML report, in which all the results from the analysis are gathered and presented within several windows, each of which contains interactive links both to external website which provide the user with further information about the SEs, as well as to other internal HTML pages created by NaviSE within the report. The content of this report is discussed in detail in the “Results” section.

### Parallelisation implementation

**Fig. 3:**
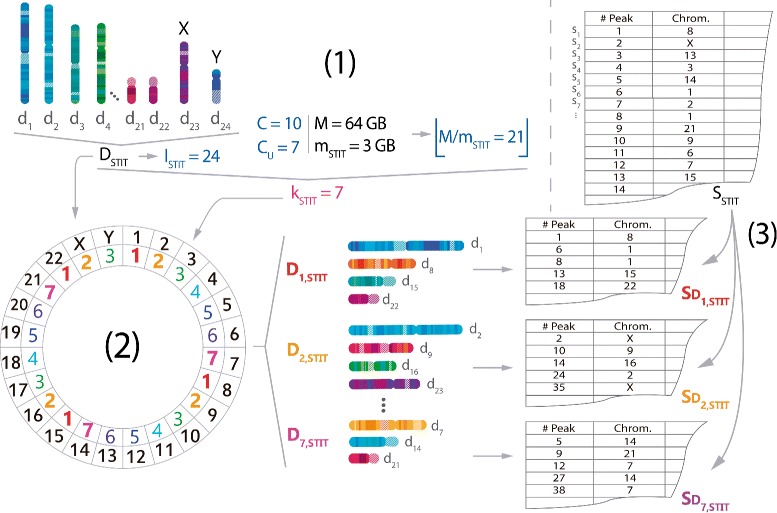
Scheme of parallelization of stitching. **1** Determination of the number of processes (*k*_STIT_) based on Eq. 4 for a case in which the number of available cores (*C*) is 10, the maximum number of cores reserved by the user (*C*_*u*_) is 7, the memory main of the computer (*M*) is 64 GB, the memory allocated to stitching (*m*_STIT_) is 3 GB and the cardinal (*l*_STIT_) of the set of chromosomes (*D*_STIT_={*d*_1_=1,*d*_2_=2,⋯,*d*_22_=22,*d*_23_=*X*,*d*_24_=*Y*}) is 24. The resulting number of allocated cores calculated by Eq. 4 is *k*_STIT_=*C*_*u*_=7. **2** Construction of data *chunks* is calculated by Eq. 5. Since *k*_STIT_=7, the set of chromosomes, *D*_STIT_, is divided into 7 subsets or *chunks*: *D*_1,STIT_={*d*_1_,*d*_8_,*d*_15_,*d*_22_}; *D*_2,STIT_={*d*_2_,*d*_9_,*d*_16_,*d*_23_}; ⋯; *D*_6,STIT_={*d*_6_,*d*_13_,*d*_20_} and *D*_7,STIT_={*d*_7_,*d*_14_,*d*_21_}. **3** Assignment of information elements. In the case of stitching, assigned elements are MACS peaks (inferred as enhancers). After the assignment of the subsets *D*_1,STIT_, *D*_2,STIT_, etc., the set of MACS peaks, *S*_STIT_={*s*_1_,*s*_2_,⋯ } is divided into 7 subsets of elements, $S_{D_{1,\text {STIT}}}=\{s_{1}, s_{6}, s_{8},\cdots \}$, $S_{D_{2,\text {STIT}}}=\{s_{2}, s_{10}, s_{14},\cdots \}$, ⋯, $S_{D_{7,\text {STIT}}}=\{s_{5},s_{9},s_{12},\cdots \}$, based on the chromosome of each row. Finally, all the subsets of elements are simultaneously processed by NaviSE, combined into one file, and the SE ranks are calculated

The algorithm of parallelisation developed in NaviSE constitutes a significant improvement of performance in the analysis of NGS samples compared to non-parallelised pipelines. NaviSE determines the optimal number of processes, *k*, compatible with the computer resources as Luu et al. do in [8]. Such resources are the parallel processing capability of the computer measured as the number of cores, *C*, and the total main memory, *M*, in Gigabytes (GBs). NaviSE optimizes automatically, for each processing task *i*, the number of threads, *k*_*i*_, in which the task *i* will be parallelised by the expression:

4 $$ k_{i}=\min\left({C, C_{u}, \lfloor M/m_{i}\rfloor, l_{i}}\right)   $$

where *C*_*u*_ is the maximum number of cores reserved by the user to run NaviSE, *m*_*i*_ is the memory, measured in GBs, needed to run one process in task *i*, ⌊ ⌋ is the floor operator and *l*_*i*_ is the cardinal of *D*_*i*_={*d*_1_,*d*_2_,⋯,*d*_*m*_} which is the set of *chunks* of distributed data elements to be processed in task *i*. If *l*_*i*_>*k*_*i*_, the first *k*_*i*_ chunks are distributed to *k*_*i*_ threads. The distribution of information (number of chromosomes for stitching, SE peak distribution profiles for GVT, number of gene sets for GSEA) to be parallelised is based on a cyclic algorithm, implemented in Python, with the following outline: For the ordered set *S*_*i*_={*s*_1_,*s*_2_,⋯,*s*_*n*_} of information elements, the set *P*_*i*_={1,⋯,*k*_*i*_} of processes, and for the set *D*_*i*_ of data (chromosomes, positions on a list, gene sets) to be distributed across processors, we define *D*_*pi*_ as the *chunk* of data of the task *i* that is assigned to each processor *p*:

5 $$ {}D_{pi} = \{d_{j}\;|\; \forall d \in D_{i},\; p \in P_{i},\; j \in \{1, \cdots, l_{i}\},\;\; j\bmod{k_{i}} = p\}   $$

where mod is the module operator. Once the *chunk* *D*_*pi*_ is constructed, the subset of information elements $S_{D_{pi}} \subset S_{i}$ will be defined depending on the task *i* which is being parallelised. The list of parallelised tasks in NaviSE is *i*={STIT,GVT,GSEA,HOMER}. In the case of parallelisation of SE prediction by stitching (STIT), the set of peak coordinates from MACS (*S*_STIT_) is divided into *k*_STIT_ files, calculated with Eq. 4, with *m*_STIT_=3 GBs. Here, *D*_STIT_={1,2,3,⋯,*X*,*Y*} chromosomes, *D*_*p*,STIT_ represents the sets of chromosomes that will be processed in each *p*∈*P* calculated by Eq. 5, $S_{D_{p,\text {STIT}}}$ is the *chunk* of *s*∈*S*_STIT_ peaks which share the same chromosome from each set of chromosomes from *D*_*p*,STIT_. For a better understanding of the process, an example is developed in Fig. 3.

In the case of SE signal profile generation with GVT, *S*_GVT_≡*D*_GVT_, is the set of SE *loci*. Hence *D*_*p*,GVT_ contains all the *loci* that fulfill Eq. 5, based on *k*_GVT_ calculated with Eq. 4 with *m*_GVT_=2 GBs.

In the case of GSEA parallelisation, *D*_GSEA_ is the set of combinations (GSEA signatures × GSEA cutoffs) and *S*_GSEA_ is the set genes associated to SEs and TEs up to the corresponding GSEA cutoff. Therefore, *D*_*p*,GSEA_ contains all the combinations that fulfil the Eq. 5, based on *k*_GSEA_ calculated with Eq. 4 with *m*_GSEA_=2 GBs.

The parallelisation of all these tasks has been implemented with the *multiprocessing* module of Python. In the case of HOMER parallelisation, we took advantage of the capabilities already implemented in HOMER, with the number of processes *k*_HOMER_, optimized by Eq. 4, with *m*_HOMER_=2 GBs.

## Results

To illustrate the performance of NaviSE, we have selected H3K27ac histone mark whose raw signal data has been downloaded from the GEO database [20] for three cell types: human Embryonic Stem Cells (ESC) (GSM663427, with control GSM605335), monocytes (MON) (GSM- 1003559 with control GSM1003475) and neurons (NEU) (GSM2072642, with control GSM2072639). For other analysis, we also used H3K4me1 (GSM409307) and H3K4me3 (GSM409308) from ESCs.

### HTML report generation

The output of NaviSE for each experiment is a collection of HTML linked pages whose main page contains dynamic graphical elements, namely, a blue horizontal ribbon with links to all the HTML pages from the report, detailed below; a grey sidebar by which the user can access the different subsections; and a window in which the results are displayed.

**Fig. 4:**
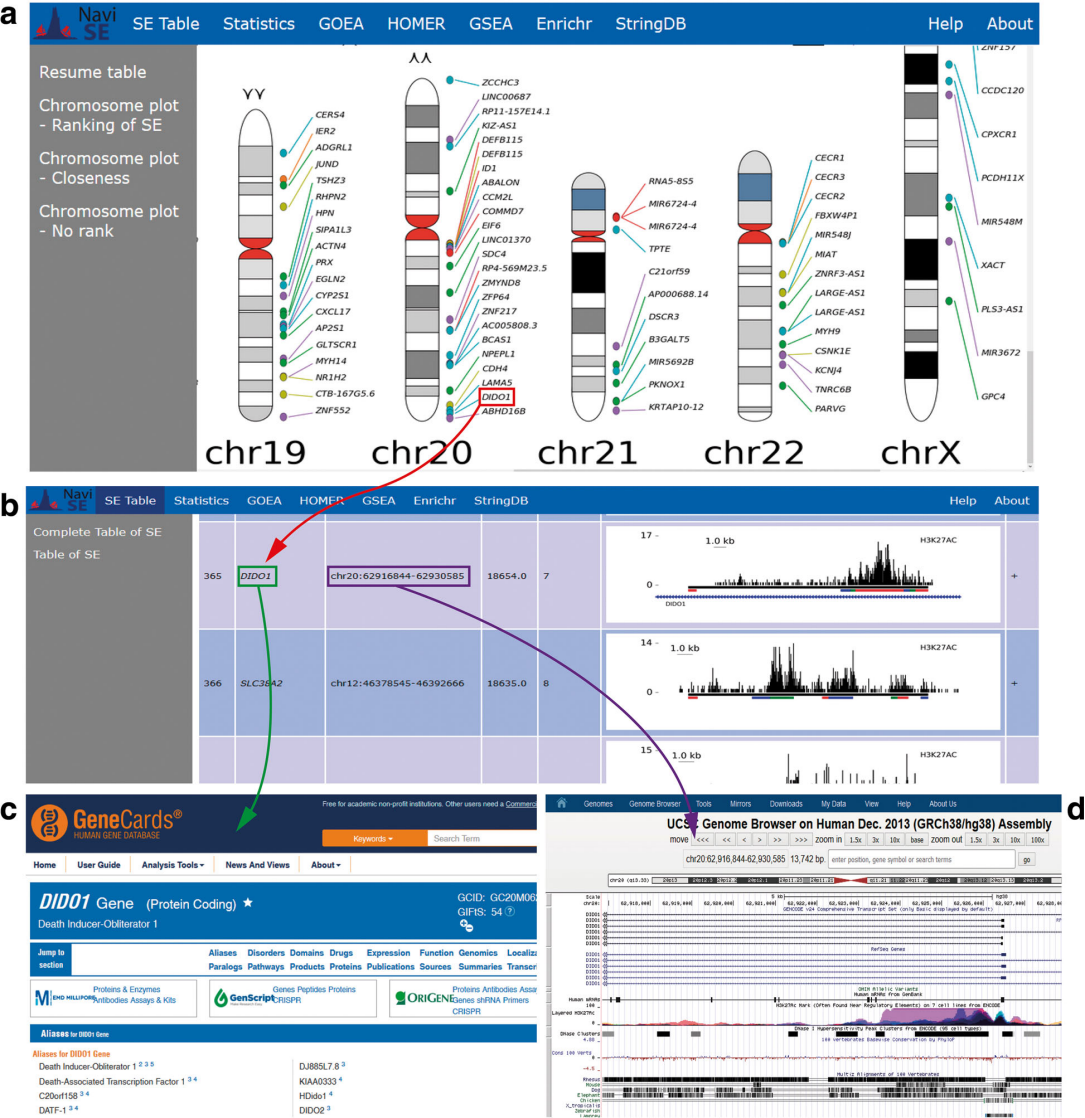
NaviSE GUI. All NaviSE windows contain a navigation bar on the top with links to all the results windows. On the *left side* there is a side *menu* bar with links to subsections of the active window. **a** The *main window* of NaviSE depicting the chromosomal plot in which the positions of all predicted SEs are mapped into a karyotype. Each SE in this window is a *hot-spot* with a link to the SE table. **b** Amongst other features, *SE table* contains the ranking of SEs, the names in the SE table linked to GeneCards (**c**), the chromosomal locations linked to UCSC Genome Browser (**d**), the SE score, the number of subpeaks, and, in the last column, the SE signal profile drawn with our GVT module

The *main window* contains basic information about the analysis and different chromosomal plots, defined in the point 9 of “SE prediction and annotation” section, represented in the chromosomal plot snapshot of Fig. 2. The chromosomal plot includes links to the SEs in *SE Table* window of the final report, which includes general information about each SE (genomic location, number of subpeaks, SE score), alongside with a snapshot of the SE genomic signal profile, included for visual evaluation of the SE quality, together with the quantitative SE score. The *SE Table* columns referring to gene names and genomic location include, respectively, a link to GeneCards site [21] and UCSC Genome Browser [22], as shown in Fig. 4.

*Statistics* window implements a series of graphs which allow the user to obtain information related to the SEs in the sample. Some of those graphs are analysed thoroughly in the corresponding *Analysis of different cell lineages* “Results” section.

*GOEA* window includes the results from the GOEA. At first, a barplot shows the significant terms from GO categories (biological process, cellular component and molecular function) which, upon clicking, will lead to a Directed Acyclic Graph (DAG) of the GO terms associated with the significant term, each of which contains the related genes associated to that term. Below the barplot, there is a table that leads to the DAG for the corresponding GO term, which includes values such as enrichment ratio of the predicted cell population, and the False Discovery Rate (FDR) for each term.

**Fig. 5:**
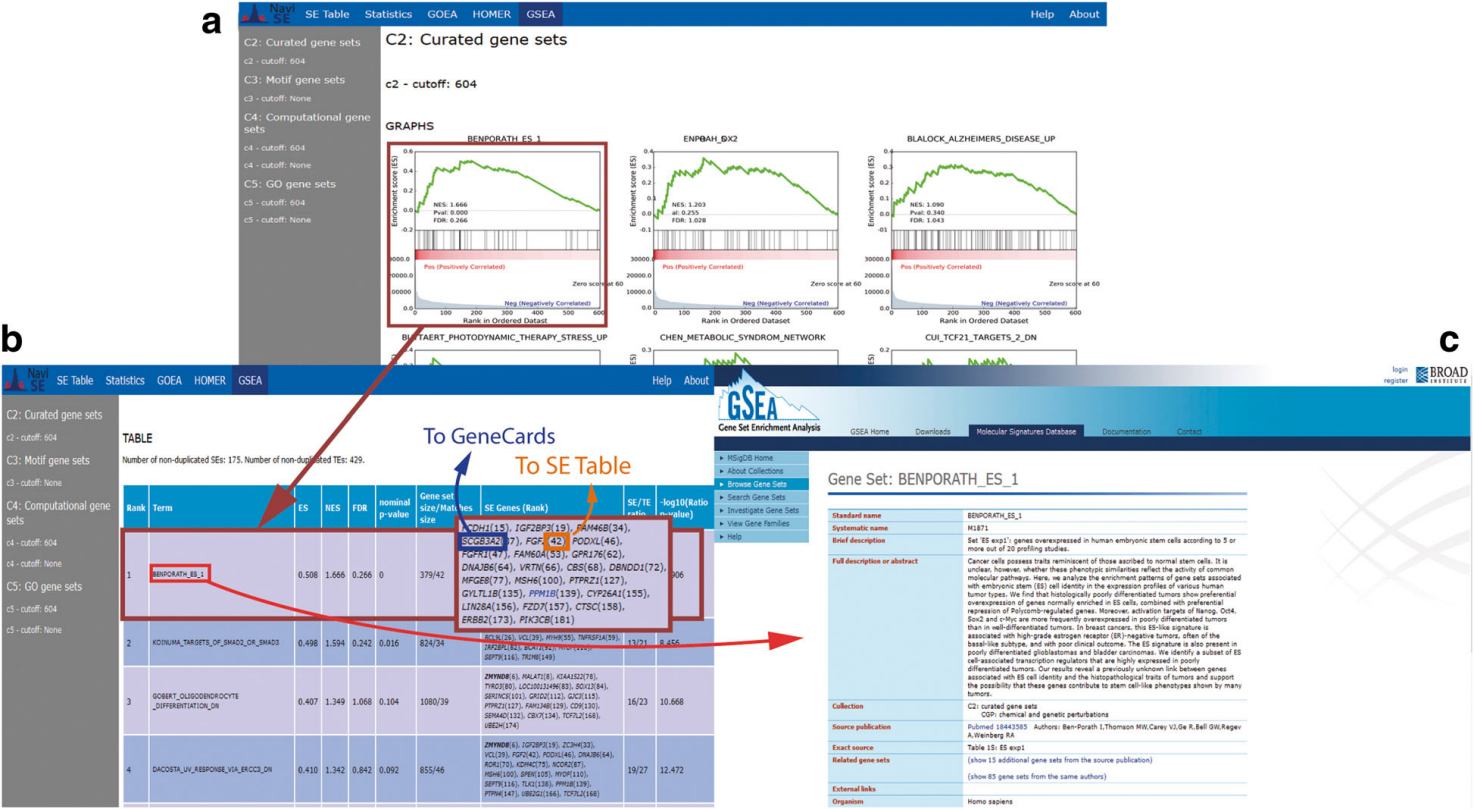
GSEA NaviSE GUI. **a** Window with interactive links to the corresponding GSEA terms in the *GSEA table*. **b** Table with the ranking of GSEA terms; each GSEA term, linked to GSEA website (**c**); statistical values such as Enrichment Score (ES), Normalized Enrichment Score (NES), FDR and *p*-values provided by GSEA; list of associated genes, linked to GeneCards with SE ranking value (in parentheses) linked to *SE table* window, described in Fig. 4 b

Similarly, the *GSEA* window (Fig. 5) contains several graphs depicting the GSEA profile of the significance of the analysis, for each signature (group of gene sets) and threshold. Clicking on a graph leads to its corresponding information element on a table below, which contains several related values, such as the significant GSEA term, related SE genes, and statistical values linked to the GSEA term such as Enrichment Score (ES), Normalized Enrichment Score (NES), FDR and *p*-values provided by GSEA, which are further described in Additional file 1.

*HOMER* window shows the results from the motif analysis by the HOMER tool, which includes two ranked tables, one for known motifs and another one for *de novo* motifs. ‘Known motifs’ table contains a LOGO image for each motif and the name of the TF or binding protein using such binding motif. It also includes the percentage of SE and TE sequences that has such motif, and a *p*-value that measures the statistical significance of the association of the SE with such motif. The ‘*de novo*’ table includes motifs predicted by HOMER to bind elements differentially in SEs and TEs. Upon clicking on each element in the ‘*de novo*’ table, NaviSE redirects to a HOMER-generated page that includes more information about the motif.

Finally, *StringDB* and *Enrichr* windows show, respectively, PPI networks from SEs at different confidence values; and results from Enrichr website including TFs related to SEs, cell or tissue specification or metabolic pathways linked to the SE population. Each subsection includes a barplot of the significant terms which link to the elements in a specific table. This window is described in detail in Additional file 1.

### Process parallelisation

**Fig. 6:**
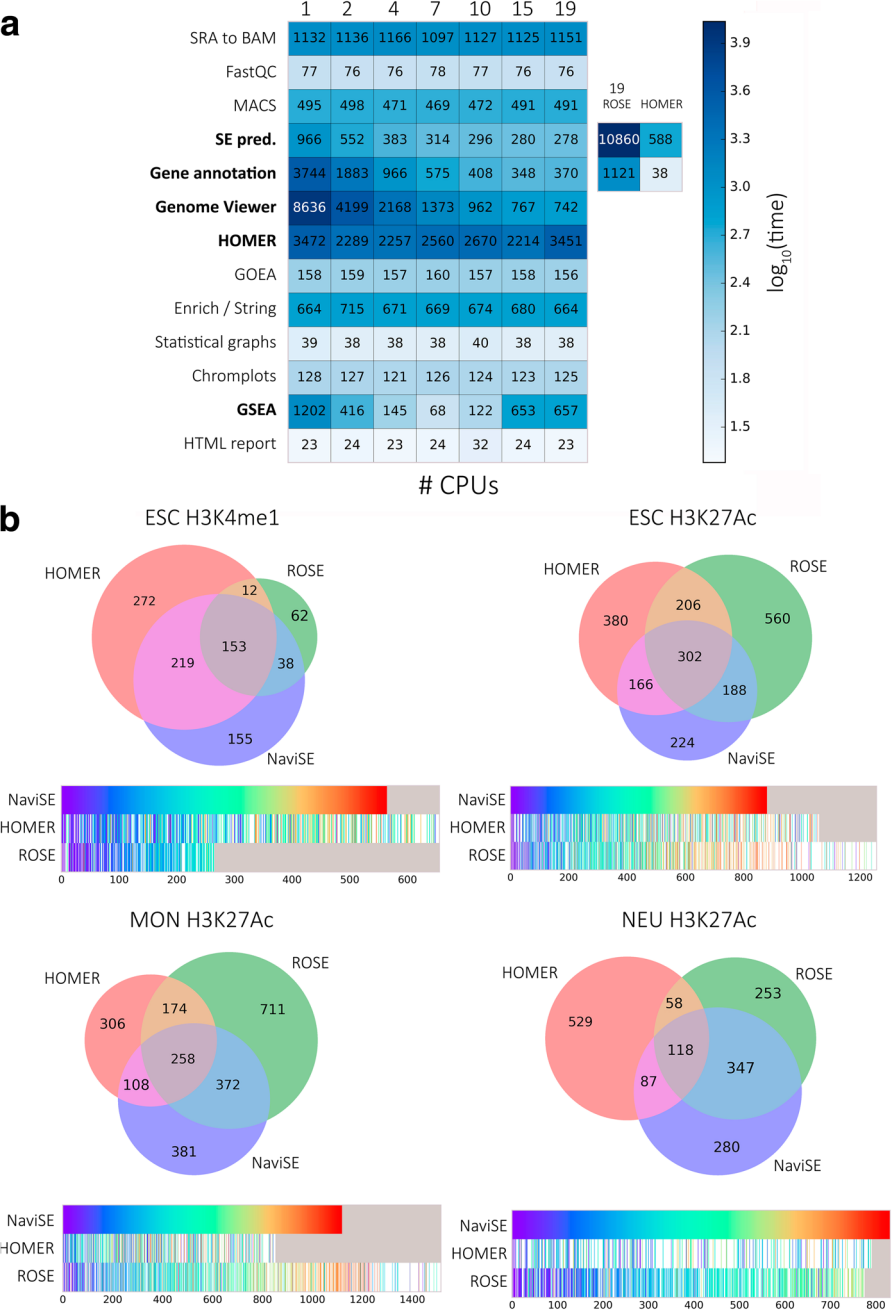
NaviSE peformance comparisons. **a** NaviSE CPU running time for different numbers of CPUs. Heatmap of the processing time for each NaviSE process for different numbers of CPUs, writen on top. Tasks parallelised by NaviSE are highlighted in *bold* typeface. For the SE prediction and gene annotation, running times of ROSE and HOMER on 19 CPUs are also provided. **b** SE prediction similarities among different software. For each cell type and histone mark, the Euler-Venn diagram with the number of commonly predicted SEs is represented on top and the comparisons among the SE ranking generated by the different software at the bottom. The rank of each SE predicted by NaviSE is colour-codified (*bluer* colours indicate higher positions in the rank and redder colours lower positions). Each NaviSE SE is mapped onto HOMER and ROSE SE ranking tracks in the position predicted by HOMER and ROSE for such SE, with the colour codification corresponding to the ranking predicted by NaviSE. SEs predicted by other software that are not predicted by NaviSE appear in white. *Grey* boxes mark the indexes for which a rank in a predictor has exhausted its number of predicted SEs in comparison to the maximum rank predicted by the three software {HOMER, ROSE, NaviSE}

The parallelisation of NaviSE is fundamental to save time during the data processing, more so when the analysis is performed simultaneously with numerous cell types or marks. The computing time optimization achieved upon NaviSE parallelisation is shown in Fig. 6 a.

Most time-consuming processes show a considerable decrease in running time: in SE prediction up to a 30% of the original time, in gene annotation up to 10%, and in GVT up to 8.5%. TF prediction by HOMER, and GSEA, are also parallelised, although, interestingly, their optimal processing time achieved is obtained using between 4 and 7 processors, probably due to limits in main memory usage or difficulties of the Python-operating system interface for managing the optimal access to all the CPUs. In short, the overall amount of time is reduced up to a 40% between 1 and 19 processors, and the optimal difference is achieved at 15 processors, with a reduction up to 30%.

Hence, NaviSE shows a considerable reduction of processing time even with small processing capability, below 6 CPUs, which may allow conducting research with mid-range computers. As for the non-parallelisable processes, they involve very fast computing tasks that do not require parallelisation or in which the algorithm shown in Eq. 5 cannot be efficiently implemented (such as reading files, alignment of reads or processing of some tables).

### Comparison of SE predictions among different software

As previously mentioned, NaviSE performs the whole processing from raw data files to comprehensive annotations of SEs. However, there are alternative software packages that can perform the SE prediction task as well. Here, we compare NaviSE SE predictions with those obtained with ROSE and HOMER, in ESCs epigenomics data.

The stitching calculation that we have implemented in NaviSE is much faster than the ones implemented in ROSE and in HOMER. For example, when running in 19 CPUs, the stitching of NaviSE takes 278 seconds, whereas the stitching of ROSE takes 10,860 s (39 times slower than NaviSE) and the stitching of HOMER 588 seconds (2.1 times slower than NaviSE) (Fig. 6 a).

It has to be taken into account that NaviSE does not only annotate the SE peaks but also the subpeaks. This feature provides NaviSE with an important annotation feature to understand the SE structure that HOMER cannot provide.

To test the similarity between the SE predictions produced by the different software, we have used ESC, MON and NEU cell types. Although NaviSE, ROSE and HOMER predict different number of SEs, they share a significant number of predictions (see Euler-Venn diagrams in Fig. 6 b). To analyse deeply the similarities among these predictions we have designed a graphical representation that allows us to track the ranking of each SE predicted by each software in comparison with the ranking predicted by NaviSE. This representation shows that the rank of the score of the SEs is very similar among all of the predictors (ranking bars in Fig. 6 b). A detailed explanation of prediction divergences between different software, as well as between epigenomic combinations, is provided with an example with ESCs at “NaviSE epigenomics signal algebra is able to predict SEs with sharper signals” “Results” section.

### SE prediction of different cell lineages

To assess the capabilities and performance of NaviSE, we have run several real datasets from different species (human and mouse), histone marks (H3K27ac, H3K4me3 and H3K4me1), and cell types (ESC, MON and NEU), using the hg38 human genome version.

#### Main page, SE table, and Statistics

**Fig. 7:**
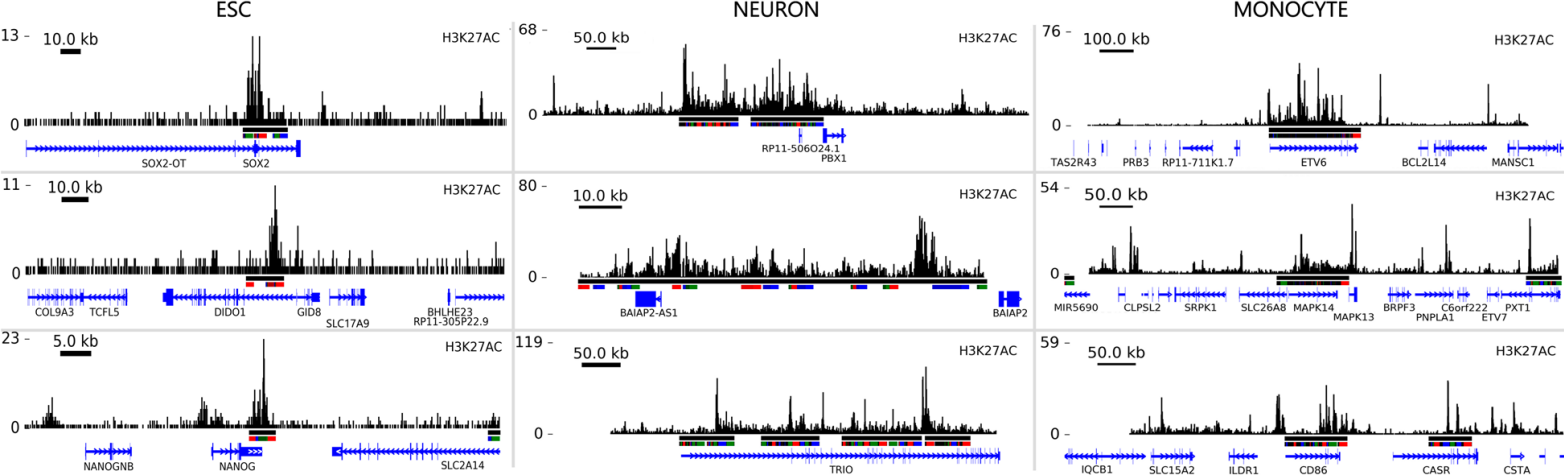
SE ChIP-seq peak distribution. Box plot of peak distribution for each SE obtained with our GVT module, for some representative SE predictions. Each column represents SEs from each cell type. *Black lines* below the signal represent the SE supports at each sample, and bars in alternating colours below SE bar show the supports of the SE subpeak composition

**Fig. 8:**
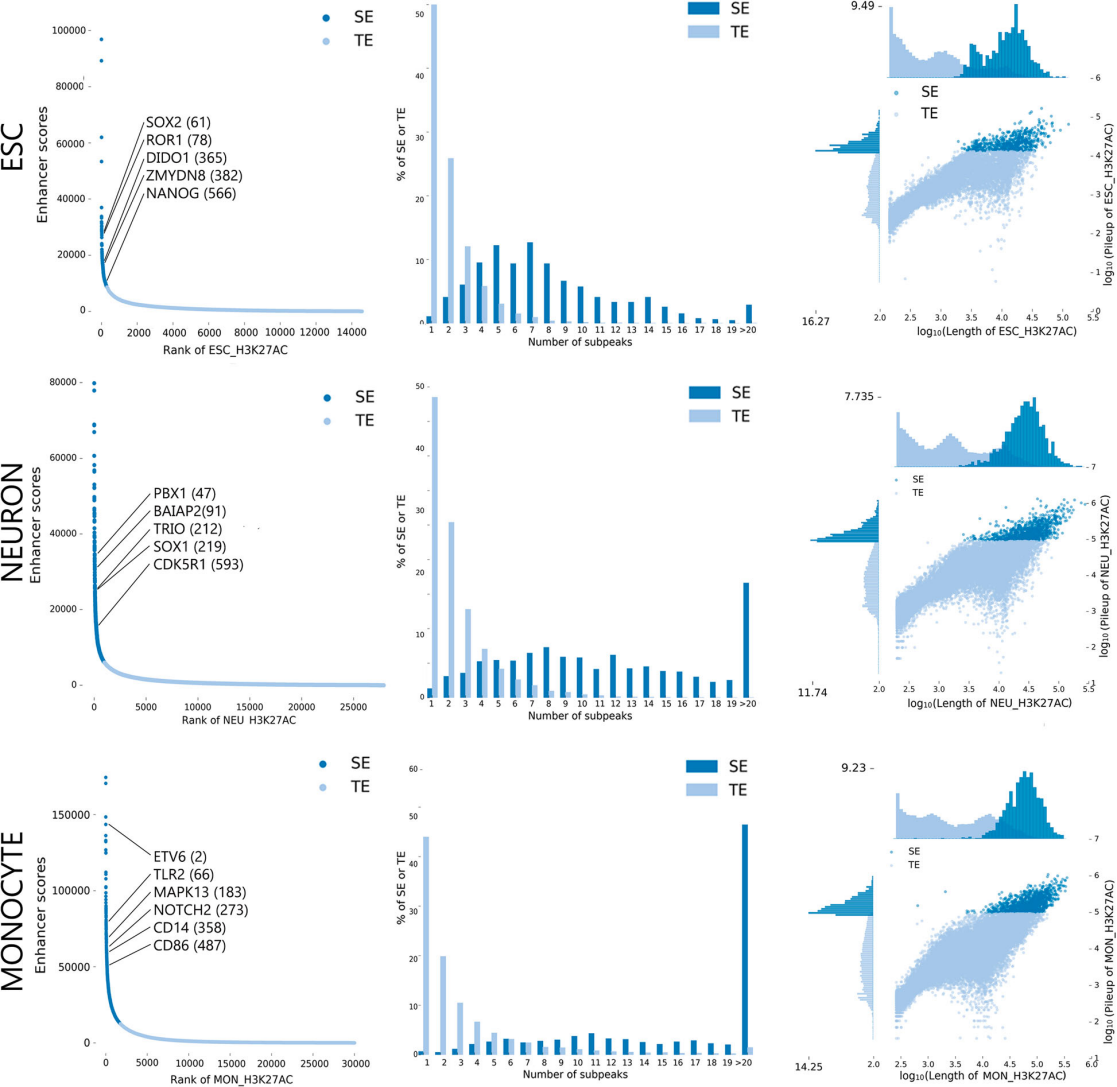
NaviSE GUI statistics. Diagrams of statistical parameters for each cell type, arranged in rows. SE ranking (Eq. 2) by ChIP-seq signal (*left column*) with the most relevant SEs of each cell type and their corresponding ranking, SE distribution of the number of subpeaks of the SEs (*center column*) and SE pileup *vs* length scatter plots in log_10_ scale with the respective distributions of SE pileups in ordinates and SE lengths in abscissas (*right column*)

Using the same default parameters with H3K27ac histone mark, the NaviSE analysis for the different cell lines yielded a wide range of SEs (*n*_*ESC*_:664, *n*_*NEU*_:1073, *n*_*MON*_:1235). The signals of the most important SEs are shown in the Fig. 7 and the main statistics for each cell type are depicted in the Fig. 8.

The distribution of subpeaks varies considerably between SEs and TEs. TE subpeak distribution follows a Zipfian-like distribution in all the analysed cell lines, that is, most of the samples contain only 1 subpeak, and the number of samples that contain higher amount of subpeaks goes down at a rate of ∼50% of the previous number of subpeaks; whereas the SE distribution might follow a *χ* ^2^ distribution or a normal distribution. In the case of ESCs, the maximum of subpeaks is between 5 and 7, whereas in NEU and MON the distribution is uniform between 6 and 14 subpeaks, with a considerable amount of SEs having more than 20 subpeaks.

The differences in length distribution between TEs and SEs are apparent in all samples. Interestingly, TEs usually show a bi or trimodal distribution with maxima at ∼100, ∼1000 or ∼10,000 nt in all the analysed cell types, whereas SEs show a monomodal normal-like distribution with means around 25,000 - 50,000 nt. On the other hand, subpeak distribution shows no significant differences between SEs and TEs, both in length and pileup.

#### HOMER analysis

The results of the most relevant TFs revealed by HOMER are shown in Table 1. Although all three cell lines showed shared TFs such as *TCF3*, each cell type contained a set of cell-specific TFs. For instance, ESC contained *NKX2-2* and *NKX2-5*, involved in heart and nervous development; NEU contained *RXR*, involved in neural development, *NR5A2*, involved in embryonic development and, interestingly, *RUNX1*, thought to be involved in hematopoiesis. Finally, MON contained *GATA2* and *GATA1*, the first closely related to hematopoiesis and the second involved in the switch of fetal hemoglobin to adult hemoglobin.

**Table 1:** The most relevant TFs, and their binding motifs for all cell types obtained from HOMER analysis. *p*-values are presented in their *integer* logarithmic form (p*P-val* ≡− log10*P-val*)

ESC			NEU			MON		
TF	Motif	p*P-val*	TF	Motif	p*P-val*	TF	Motif	p*P-val*
*NKX3-2*		121	*TCF3*		142	*TCF3*		142
*NKX2-2*		83	*TBX21*		132	*TEAD2*		105
*NKX2-5*		77	*RXR*		124	*NPAS2*		90
*ESRRA*		75	*RUNX1*		124	*GATA2*		85
*TBX5*		75	*NR5A2*		122	*GATA1*		83

#### GOEA and GSEA results

**Fig. 9:**
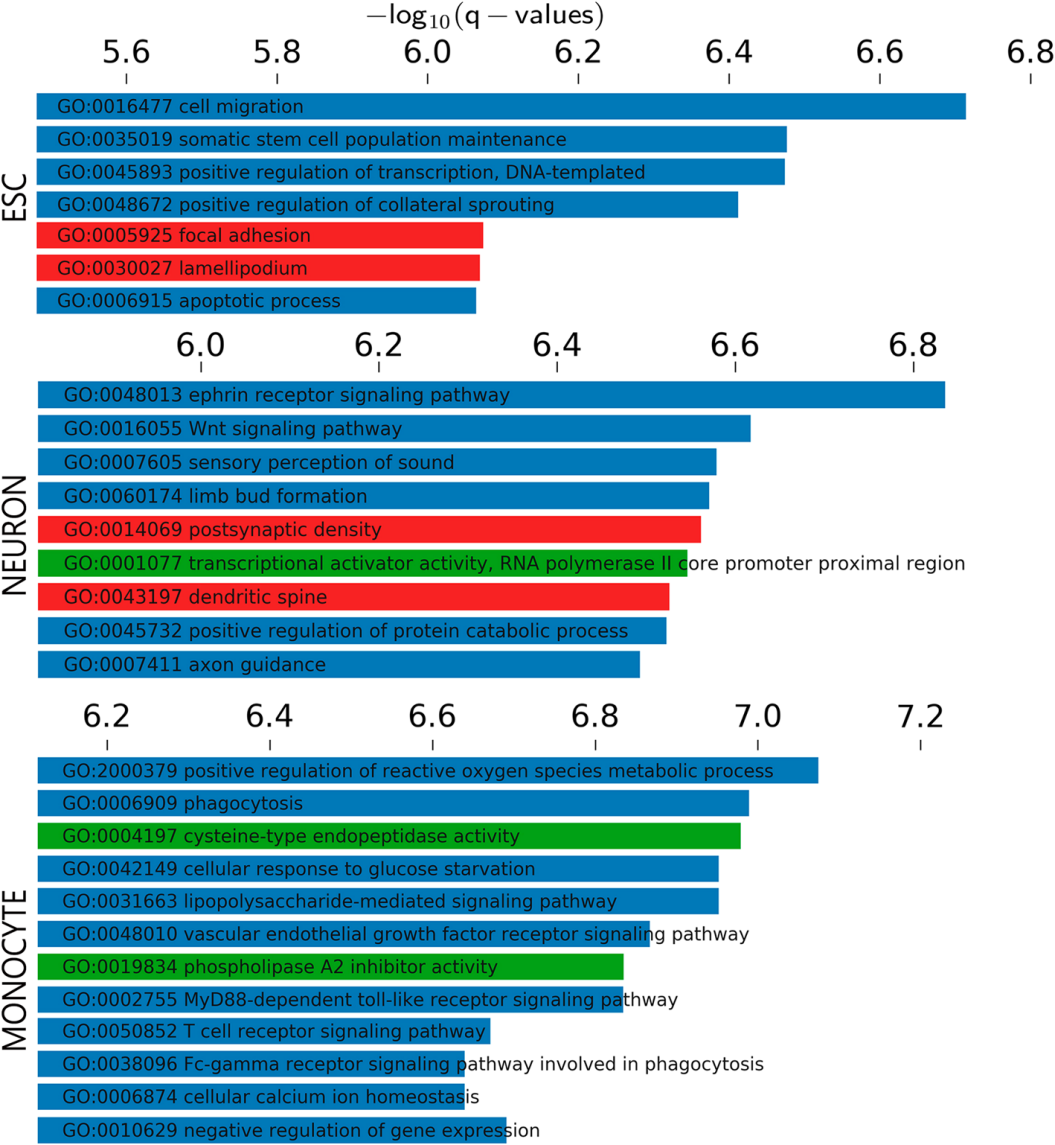
NaviSE GUI GOEA significant terms. *Bar* plots for each cell type depicting the most relevant and statistically significant terms for GOEA of the genes associated with SE predicted for H3K27ac. *Red* - cellular component, *blue* - biological process, *green* - molecular function

**Fig. 10:**
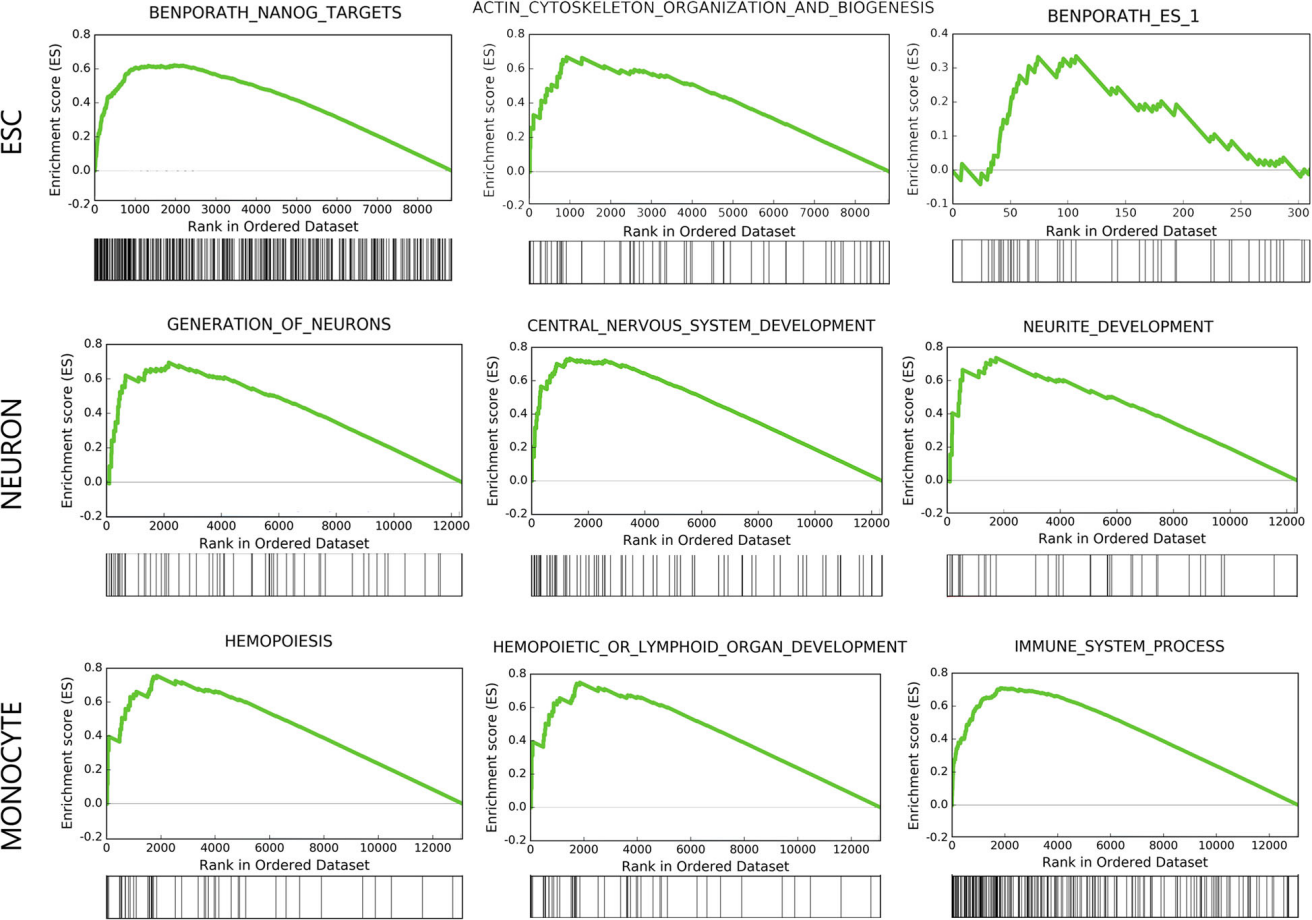
NaviSE GUI GSEA most significant terms. GSEA profiles depicting three significant GSEA sets, from MSigDB, for each cell type for genes associated with SE predicted for H3K27ac. Each graph contains the typical GSEA profile alongside its positive matches in the bar below

GOEA and GSEA are represented in Figs. 9 and 10 respectively. Both results are related, as the signatures used for GSEA contain sets of genes related to GO sets. Both analysis show correlation of functions to each cell type.

For ESC, the most relevant GO terms are related to protein expression (*positive regulation of transcription*), rearrangement of cellular morphology (*focal adhesion, lamellipodium*) or pluripotency (*somatic stem cell population maintenance*). As for GSEA, significant terms are related to master TFs of ESCs, such as *NANOG*, or cytoskeletal reorganization. Among the predominant genes, most of them repeated in several functional terms, we remark *ROR1* (which modulates neurite growth and is highly expressed during early embryonic development [23]), *ZIC3/5* (involved in the formation of right/left axis during development, and direct activator of *NANOG* promoter in ESC [24]) or *SOX2* (one of the Yamanaka’s reprograming TFs, used for the induction of pluripotency, as well as a core pluripotency factor in ESC [25]).

Regarding NEU, the most relevant GO terms are related to neural development (*ephrin signaling*, *Wnt signaling pathway*, *dendritic spine*, *axon guidance*). As for GSEA, three relevant terms are *generation of neurons*, *neuron differentiation*, and *neurite development*. Three highly ranked genes in these GSEAs are *CDK5R1* (neuron-specific activator of cyclin-dependent kinase 5, required for proper development of the central nervous system, also found essential for oligodendrocyte maturation and myelination [26]), *BAIAP2* (brain-specific angiogenesis inhibitor binding protein, might be related to neural growth-cone guidance, dendritic spine development and NMDA receptor regulation [27]) and *PBX1* (regulates differentiation and survival of certain neurons, and is impaired in Parkinson’s disease [28, 29]).

Regarding MON, the most relevant GO terms are related to specific functions of monocytes involved in immune response (*phagocytosis*, *T cell receptor signaling pathway*, *MyD88-dependent toll-like receptor signaling pathway*, *lipopolysaccharide-mediated signaling pathway*). As for GSEA, three relevant terms are *T cell receptor signaling pathway*, *reactome immune system* and *immune system process*. Genes shared by several GO terms are *NOTCH2* (related to hematopoiesis), *CD14* (one of the main markers of monocytes), *TLR2* (Toll-like receptor 2, which plays a fundamental role in pathogen recognition and activation of innate immunity [30]), *MAPK13* (is activated by proinflammatory cytokines and cellular stress [31]) or *LYN* (might be involved in the regulation of mast cell degranulation, and erythroid differentiation [32, 33]). Interestingly, *NOTCH1* gene, which is essential for hematopoiesis [34], does not appear in the list of SEs predicted by NaviSE for this dataset.

#### Enrichr analysis

We performed an Enrichr analysis in order to search genes involved in cellular processes related to each cell type. Most of the found genes, if not mentioned previously, appeared in GSEA and GOEA as well.

For ESC, the Enrichr Reactome presents several terms such as *transcriptional regulation of pluripotent stem cells*; and *POU5F1, SOX2, NANOG genes related to proliferation*, widely related to embryogenesis. Predominant genes are *FGF2* (implicated in a multitude of physiologic and pathologic processes, including limb development, angiogenesis, wound healing, and tumour growth [35]), *SOX2* or *NANOG* (TF belonging to Homeobox proteins, critically involved with self-renewal of undifferentiated ESCs, which is also one of Thomson’s reprogramming factors [36]). ENCODE and Chromatin Enrichment Analysis (ChEA) TFs includes TFs related to pluripotency (*TCF3*, *NANOG*, *SOX2*, *POU5F1* and *KLF4* as the most relevant) which share several genes, such as *ZMYDN8*, or *DIDO1* (involved in apoptosis, autophagy, and meiosis). Interestingly, and as described by Hnisz et al. [4], we found that the SEs predicted by NaviSE are capable of disclosing a crosstalk between TFs (for instance, all the aforementioned TFs interact with *SOX2* and *NANOG*, according to ENCODE).

As for NEU, Reactome includes significant terms such as *axon guidance* or *semaphorin interactions*, with genes such as *TRIO* or *CDK5R1*; which also appear as genes associated with several TFs such as *REST* (transcriptional repressor that represses neuron-specific genes, such as type II sodium channel gene [37, 38]), determined by ENCODE or TRANSFAC. A gene predicted to associate with *REST* is *SOX1*, a known neuronal marker.

Regarding MON, Reactome presents several terms such as *immune system*, *innate immune system*, *hemostasis* or *toll-like receptor 2 cascade*, widely related to monocytes, whose associated genes are *TLR2*, *FOS* (implicated as regulator of cell proliferation, differentiation, and transformation, associated with B lymphocyte differentiation and involved in lypopolisaccharide and low density lipoprotein response [39–41]) or *CD86*, expressed by antigen-presenting cells. Binding of this protein to CD28 antigen is a co-stimulatory signal for activation of the T-cell. TRANSFAC and ENCODE include genes associated with TFs like *GATA1*, *GATA2*, *SPI1* or *RUNX1*, among which are *IKZF1* or *JARID2*. Enrichr also determined markers for monocytes or lymphoid cells, such as *RIN3*, *CXCR4*, *TREM1* or *ETV6*.

### NaviSE epigenomics signal algebra is able to predict SEs with sharper signals

To evaluate to which extent the use of the epigenomics algebra improves the SE predictions, we have selected combinations of activation and repression epigenetic signals and compared SE predictions of HOMER, ROSE and NaviSE in ESCs. We denote the set formed by a SE software predictor {HOMER, ROSE, NaviSE}, and the set of SEs and TEs derived from an algebra of single or combined epigenetic signals {H3K27ac, H3K4me1, H3K4me3, H3K27ac NOT H3K4me3, H3K27ac NOT H3K27me3, H3K27ac + H3K4me1 - H3K4me3, H3K27ac + H3K4me1 - H3K27me3} as *S* *T* *I* *T*_*pred*−*algebra*_. To quantify the results of the different *S* *T* *I* *T*_*pred*−*algebra*_, we collected a set of ESC core pluripotency markers from the literature [42] and built a metric of the global goodness of the *S* *T* *I* *T*_*pred*−*algebra*_ based on the SE ranking generated for each *S* *T* *I* *T*_*pred*−*algebra*_ over the set of ESC markers. As each *S* *T* *I* *T*_*pred*−*algebra*_ contains a different number of SEs (thus, producing ranks of different length), to make the different SE ranks comparable, we designed a transformation to re-scale each SE rank, *r*, given by Eq. 2 into a scaled rank *s*(*r*) as follows:

6 $$ s(r) = \frac{r}{\left\vert{STIT_{pred-algebra}}\right\vert}\cdot 100   $$

where |*S* *T* *I* *T*_*pred*−*algebra*_| is the number of SEs predicted by each *S* *T* *I* *T*_*pred*−*algebra*_. Thus, when we apply Eq. 6 to scale the rank *r*, it produces a *s*(*r*) in the range [0,100] if the epigenomics signal algebra is predicted as a SE, and *s*(*r*)>100 if the signal algebra is predicted as a TE or is not predicted at all. Better 1 *S* *T* *I* *T*_*pred*−*algebra*_ assigns lower *s*(*r*)s to the SEs associated to ESC gene markers.

**Fig. 11:**
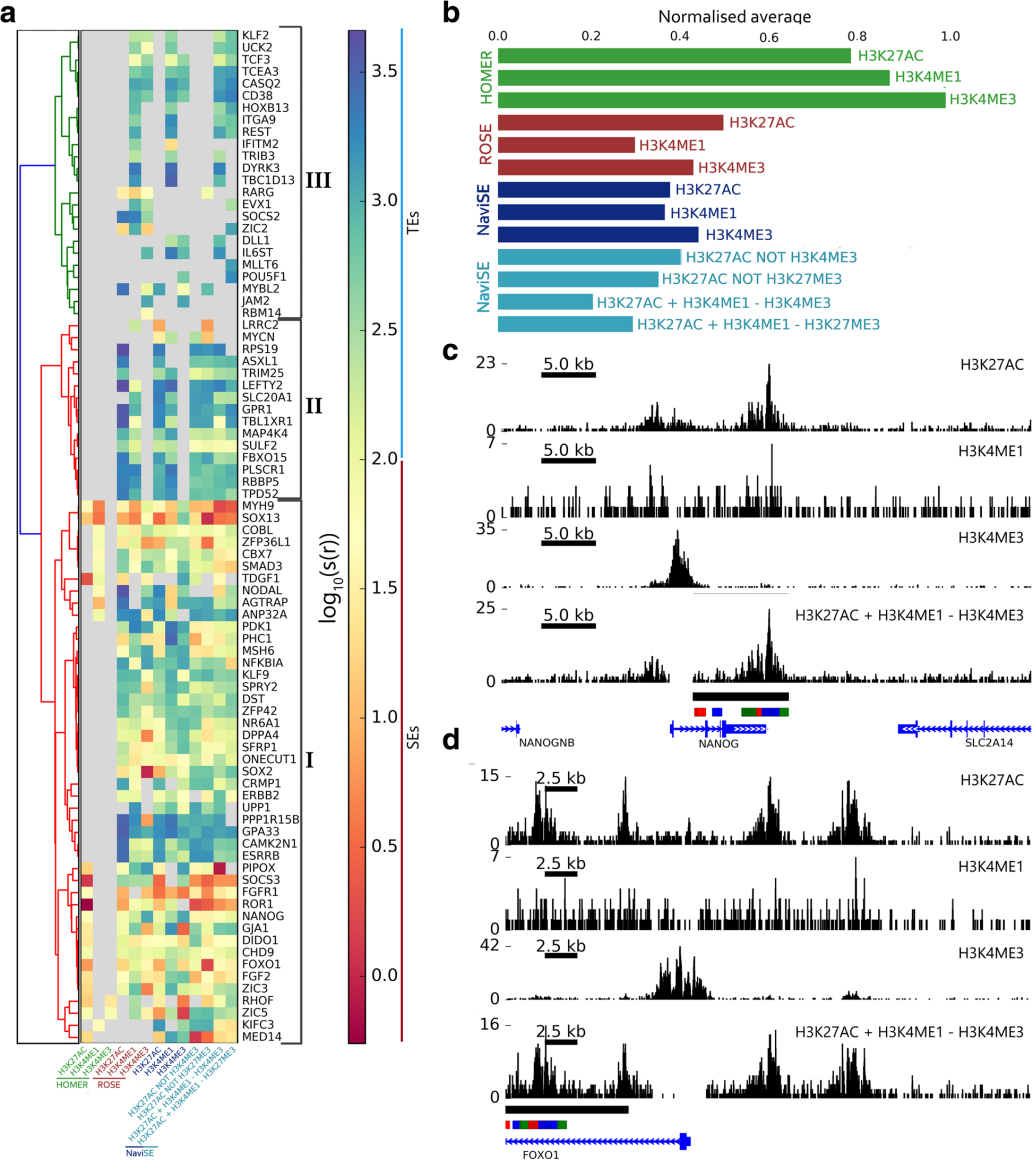
Performance of the epigenomics algebra on ESC gene markers. **a** Heatmap of the scaled ranking *s*(*r*) calculated by Eq. 6 for the SEs predicted by different *S* *T* *I* *T*_*pred*−*algebra*_ for ESC gene markers. The scaled ranking is colour coded in log_10_ scale, in *red* for good ranked (low ranking values) SEs, in *yellow* (from 2.0 onwards) for good ranked TEs, in *green* and *blue* for bad ranked TEs, and in *grey* for TEs without signal prediction. **b** Global goodness of each *S* *T* *I* *T*_*pred*−*algebra*_ over the whole set of ESC gene markers; normalised to the predictor of highest average (HOMER H3K4me3). Epigenomic algebra and single epigenomic signal box plot of peak distribution, depicted by our GVT module, for the SE associated to *NANOG* (**c**) and *FOXO1* (**d**). The *bottom row* contains the combination of epigenomic signals, and the rows above contain the original single signals. *Black lines* below the signal represent the SE supports at each sample, and bars in alternating colours below SE bar show the supports of the SE subpeak composition

To quantify the global performance of each *S* *T* *I* *T*_*pred*−*algebra*_, we calculated the average $\overline s$ of *s*(*r*) over the list of all ESC markers. Therefore, the best *S* *T* *I* *T*_*pred*−*algebra*_ will produce the lowest $\overline s$. We depict the *s*(*r*) for the list of ESC gene markers and the list of *S* *T* *I* *T*_*pred*−*algebra*_ in the heatmap of Fig. 11 a.

We observe three main patterns of behaviour, a group I of genes (from *MED14* until *MYH9*) that has associated a majority of SEs predicted by almost all the *S* *T* *I* *T*_*pred*−*algebra*_, some of them not by HOMER, a group II (from *TPD52* until *LRRC2*) that has associated TEs predicted by ROSE and NaviSE *S* *T* *I* *T*_*pred*−*algebra*_ but not by HOMER, and a group III (from *RBM14* until *KLF2*) that has associated lower ranked TEs from some of the combined algebras of NaviSE.

Interestingly, no *S* *T* *I* *T*_*pred*−*algebra*_ predicts SEs associated with the master regulator of pluripotency *POU5F1/OCT4* (they appear as TEs with H3K4me3 and H3K27ac + H3K4me1 - H3K27me3 from NaviSE), suggesting that *POU5F1* has a subtle epigenomic regulation that hinders the discovery for upstream *POU5F1* regulators, as it has been observed in the computational attempts with unconstrained discovery algorithms to find *ab initio* motifs regulating the *POU5F1* promoter [43].

The plot in Fig. 11 b depicts the normalised metric of global performance $\overline s$ of each *S* *T* *I* *T*_*pred*−*algebra*_, where the lowest values are associated to the best performance. We observe that HOMER-based predictors show the worse performance, NaviSE single epigenomic signal SE predictions are better than those of HOMER and ROSE, and NaviSE H3K27ac + H3K4me1 - H3K4me3 algebra is better than any other single epigenomic signal SE predictions, thus showing the advantage of using the NaviSE epigenomic signal algebra to perform SE predictions.

To illustrate how the profiles of the combined epigenetic signal algebras are developed, we selected the best performing algebra (H3K27ac+H3K4me1-H3K4me3) and depicted its resulting combination and component signals profiles H3K27ac, H3K27me1, H3K4me3 for *NANOG*, (Fig. 11 c) and *FOXO1* (Fig. 11 d). In both cases, the deletion of the H3K4me3 promoter signal upstream and over the first exon and intron shortens the SE support to focus the SE support upstream of these genes.

Therefore, although there might not be a ‘gold standard’ on what a real SE is, we can conclude that the SE predictions of NaviSE are better than other predictors’, with the added advantage to be fast obtained, fully automatized and comprehensively annotated.

## Conclusions

We designed NaviSE to perform automatic parallelised SE prediction from genome-wide epigenetic signals, or an algebra of them, due to an optimization that reduces the necessity of inputting most of the parameters, providing a comprehensive annotation of SEs. NaviSE SE annotation runs from the motifs of TFBSs enriched in SEs through functional analysis (GOEA, GSEA and enriched metabolic pathways) to PPI networks to a broad tissue prediction, thus, covering a wide range of valuable information. Such integrated annotation is of paramount importance due to the regulatory nature of the SEs, which have been described as key players in the determination of cell fate and in the involvement in the mechanisms of disease. Simultaneously, NaviSE performs all these tasks optimizing the use of the computer resources, identifying the available cores and main memory, and takes maximum advantage of them in function of the task requirements.

Furthermore, the automatic recognition of multiple file formats and the capability of working with replicates and controls, alongside with the possibility of integrating onto other pipelines or running multiple samples with multiple replicates and signal algebras at once with a simple script in Python, makes NaviSE a foremost tool for an efficient study of SEs. Due to all these capabilities, NaviSE is a time-saving and user-friendly tool for SE analysis.

To validate the biological performance of NaviSE, we applied it to predict the SEs on real data sets of several cell types with a different level of differentiation and commitment, and predicted in all cases SE-associated genes in agreement with the expected cell-specific markers. In the case of ESCs, NaviSE predicted SEs on the ESC markers *NANOG* and *SOX2*, in the case of neurons it predicted the *SOX1* and *CDK5R1* neuron markers, and in the case of monocytes, predicted the *CD86* and *CXCR4* monocyte markers.

The Additional file 1 provides a complete guide to the software installation and use instructions.

## Availability and requirements

**NaviSE. Project name:** NaviSE. NaviSE is freely available at urlhttps://sourceforge.net/projects/navise-superenhancer/.

**Operating system:** Linux 64bit (Ubuntu 11.04).

**Programming language:** Python 3.5. License: GNU GPL.

## Additional file

Additional file 1 Supplementary Information. Manual for installation, use and running examples of NaviSE. (pdf 33792 kb)

## Abbreviations

ChIP-Seq: Chromatin immunoprecipitation sequencing
ChEA: ChIP enrichment analysis
DAG: Directed acyclic graph
ESC: Embryonic stem cell
FDR: False discovery rate
GB: Gigabyte
GO: Gene ontology
GSEA: Gene Set enrichment analysis
GOEA: Gene ontology enrichment analysis
GUI: Graphic user interface
GVT: Genomic viewer tool
HOMER: Hypergeometric optimization of motif enrichment
MACS: Model-based Analysis for ChIP-Seq
MSigDB: Molecular signatures database
MON: Monocyte
NEU: Neuron
NGS: Next generation sequencing
P3BSseq: Parallel processing pipeline software for automatic analysis of bisulfite sequencing data
PPI: Protein-protein interaction
ROSE: Ranking of superenhancers
SE: Superenhancer
TE: Typical enhancer
TF: Transcription factor
TFBS: Transcription factor binding site
TSS: Transcription start site
