# Supplementary material for: NaviSE: superenhancer navigator integrating epigenomics signal algebra
Source: BMC Bioinformatics. 2017 Jun 6;18:296. doi: 10.1186/s12859-017-1698-5 (PMC5461685; doi:10.1186/s12859-017-1698-5)
Supplement: Additional file 1 — Supplementary Information. Manual for installation, use and running examples of NaviSE. (pdf 33792 kb) [file 12859_2017_1698_MOESM1_ESM.pdf]

NaviSE: Superenhancer Navigator

Documentation

Version: 1.1

Alex M. Ascensión and Marcos J. Araúzo-Bravo

2017

Contents

What is NaviSE? 3
Installation 4
- **Step–by–step installation** 4
- Basic linux programs 7
- Installing SRA-TO-BAM programs 8
- Gene Ontology 8
- Installing Goatools (Gene Ontology) 8
- Installing Genome viewer associated files 9
- Installing HOMER 9
- Installing GSEA and beatifulsoup 10
- Installing programs for Enrichr/StringDB data extraction 10
- Last but not least... setting NaviSE path 12
- **Automatic installation** 12

NaviSE Genomes 13
- Installation of other genomes 14

Commands 18
Running NaviSE 24
- Parallelization of NaviSE 29

NaviSE output 30
- Main page 31
- SuperEnhancer table 31
- NaviSE Graphs 33
- GOEA results 35
- HOMER analysis 39
- Enrichr results 41
- StringDB results 42
- GSEA results 42

What is NaviSE?

NaviSE (SuperEnhancer Navigator) is a software designed to obtain analytic superenhancer (SE) data from ChIP-seq (or similar) data. NaviSE executes a series of commands which extract information from raw data and include information about associated genes, overrepresented motifs or Gene Ontology Analysis (GOEA). All the information is gathered, processed and exported to an html file that the user can navigate and extract the relevant information from for their analysis.

NaviSE acts *mainly* as a 'program of programs', i.e. uses different software from third parties to process raw data, analyse motifs, perform Gene Ontology Enrichment Analysis (GOEA) or Gene Set Enrichment Analysis (GSEA). It also uses information from web databases (Jaspar, Transfac, String, etc.) which complement the original information. Finally, NaviSE also includes self-processed information, such as graphs or plots, which users may find supportive for the analysis of their results.

In other words, the only requirements for NaviSE to work are data from ChIP-seq experiments (data formats described in data formats) and introducing the commands on the console. All the results are presented in an html report (explained in NaviSE output) for the sake of simplicity and handiness, which allows the user to navigate through the information, and access complementary information via links.

Installation

As explained before, NaviSE works with different third-party software, so before your first NaviSE run, you will need to spend some time to install all these modules. I know that the installation is a tedious process, although, if you follow these steps carefully everything should work.

In order to download and install the files, download the original files from `https://sourceforge.net/projects/navise-superenhancer/`.In this page three different files appear: (1) **Python files** - It contains all the files NaviSE requires for proper working of the program, (2) **Programs** - It contains some installation files for third-party software, and (3) **Files** - It contains files which NaviSE requires for proper working.

First of all, download all the files and extract them into a directory where all the compressed files will be extracted. We recommend to create a general directory such as *Programs* where both NaviSE and other related programs will be located, and then extract the files into a subdirectory. In order to extract the files, right click on each compressed file and press on *Extract here* or any other similar message (this requires a uncompressing software to be installed). In total, two subdirectories (*Programs* and *Files*), along with several *.py* files should appear in the directory.

The installation steps appear on the Fig. 1.

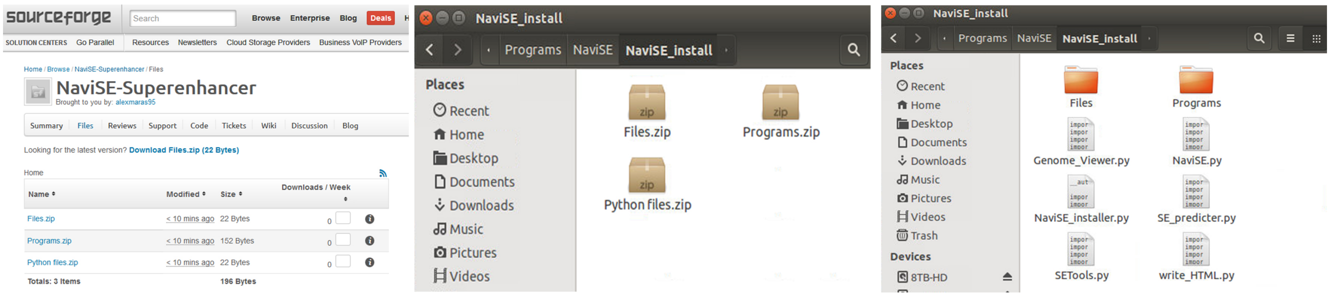

Figure 1: NaviSE binaries download.

Warning: Please, DO NOT move any of the files into other directories, nor delete any files unless they are dispensable.

Now that all the files have been downloaded, you can proceed to install NaviSE and its dependencies step by step or automatically.

Step-by-step installation

NaviSE is developed in **python 3.5**, so a python distribution is required.

Warning: Python2.7 is not allowed by NaviSE, I will never downgrade my program. Also, there is a newer python distribution, python3.6, but it still has incompatibilities with some modules, so we have to stick to python3.5.

Linux should come with its own python distribution, although it doesn't implement some modules required for NaviSE. Therefore, we must install a Python distribution which includes these modules (pandas, numpy, etc.). Our election is anaconda distribution, which includes essential packages like Numpy or Scipy, used throughout NaviSE run; and also installs dependencies related to packages when a module is installed, so manual installation of the dependencies is not required (which usually are not version wise correlated, and NaviSE may crush). Moreover, it can be used in other projects as well.

Warning: We expect users to install anaconda. If other installation lines are followed or other distributions are installed, NaviSE will not work, as it requires anaconda to set paths to he programs. We apologize for the inconveniences.

From now on, we will work via Linux command prompt (terminal), executing a series of commands. Those commands will appear in verbatim mode (`like this text`), for easier interpretation. The terminal can be prompted pressing Ctrl + Alt + T, which should appear like a black or violet window, in this fashion (Fig. 2):

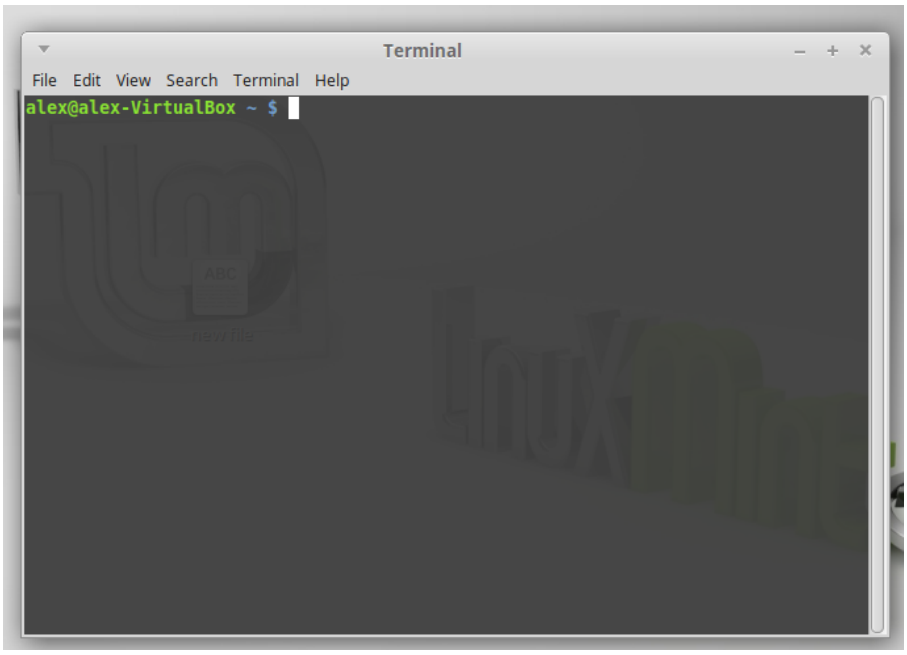

Figure 2: Basic prompt.

The first step is to download anaconda (version 3.4 in this case). It can be downloaded in two ways:

- By downloading the file (with extension .sh) from the official page.
- By downloading it via the command

```
wget https://repo.continuum.io/archive/Anaconda3-4.1.1-Linux-x86_64.sh
```

In both cases, downloaded file with a name similar to *Anaconda3-4.X.X-Linux-x86_64.sh* should appear. Now, we have to run the installation file, for which the easiest way to do is to write in the terminal `bash`, hit the Space bar, and then grab the downloaded file and drag it the terminal. A screen like this should appear (Fig. 3):

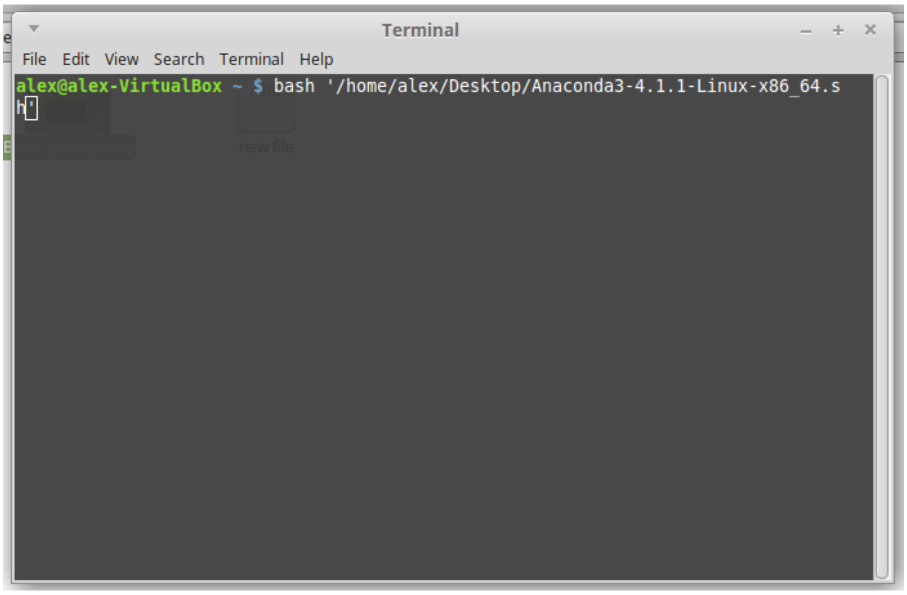

Figure 3: Anaconda download.

Hit Enter and wait. Several messages may appear, being the first that if you want to relocate your installation directory (the standard directory is the */home* directory). You can leave it as it is or write something like `/YOURUSERNAME/home/Programs/anaconda3`, as we will install other programs and it is a good practice to keep everything in the same directory.

Finally, once everything is installed, you will be asked about setting a PATH file; write `Y` and hit Enter. Remember NOT to change the directory of anaconda once installed, as it will not work. If so, you would need to change your path file of anaconda (described later).

Setting a path to a program

With the last changes I performed, it is not fully necessary to set the path to anaconda or HOMER, because NaviSE automatically recognizes the main files from the programs. However, it is highly recommendable to set the paths to those programs (NaviSE installator automatically does it) in case they will be used independently with other software on their own.

Setting a path means changing a file which tells Linux where the executables of the program are located. If the PATH is absent, changed or the file is relocated, Linux will not be able to detect the

location of this program, and when running it, it will be considered as 'non-existent'. On default, anaconda remembers the installation site and adds the PATH automatically. However, if you need to change the location of anaconda or you have not selected *Yes* when installing anaconda, the PATH must be set for proper recognition. Setting a PATH requires the following steps:

- Open the terminal and write `vi ~/.bashrc`. *vi* is a text editor and */.bashrc* is the file with the location of the paths. If it asks to create a new file, or that the file exists and you want to modify it, write `Y` and hit Enter. An empty window or a window like that should appear (Fig. 4):

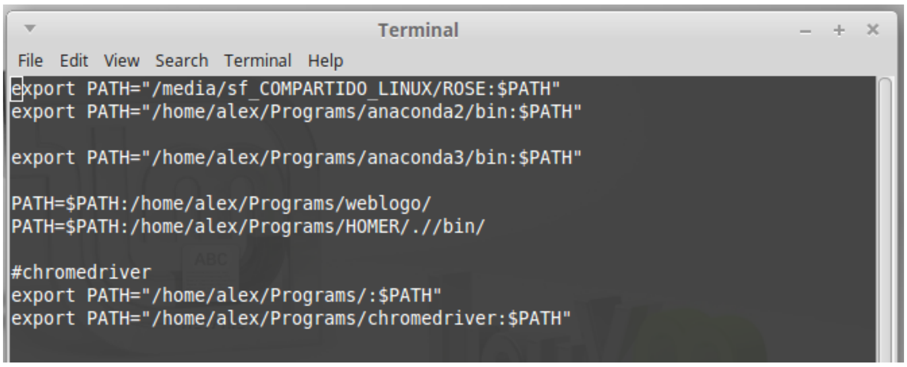

Figure 4: Vi editor and path setting.

- Enter into the edition mode by pressing i.
- Now, write the following line: `export PATH="XXX/anaconda3/bin:$PATH"` where XXX is the path of the directory where anaconda is located.
- Once the line is written, press Esc and write `:wq` to save the changes and exit.
- In order for the changes to apply, close the terminal and open it again. To reassure that the path has been correctly set, write `which anaconda`, if the output is `/XXX/anaconda3/bin/anaconda/` (XXX being the installation dir), the path is correctly set; if nothing appears, make sure the path is correctly written or that the command prompt has been restarted.

Basic linux programs

Before beginning with the installation of NaviSE components, we will need to install python2.7 and pip-2.7 to install some basic components. First, we check the presence of both programs.

For python2.7, write `which python2.7` and for pip write `which pip2.7`. If in any of them a path appears, it means that the program has been installed. If nothing appears, it means that the program in not installed still.
In order to install the programs:

- For python2.7, write `sudo apt-get install python2.7`. This process may require writing a password.

- For pip2.7, it will be installed in two steps:
  1) `wget https://bootstrap.pypa.io/get-pip.py`
  2) `sudo python2.7 get-pip.py`

Installing SRA-TO-BAM programs

For the following programs, just write each command in the prompt, press Enter and write `Y` when asked:

- MACS: `pip2.7 install macs2`
- Sra-Tools: `conda install sra-tools`
- Samtools: `conda install samtools`
- Bedtools: `conda install bedtools`
- FastQC: `conda install fastqc`

NaviSE integrates several read aligners. Bowtie2 is the aligner by defect, and STAR, BWA, and MOSAIK are other aligners which can be used to transform .fastq files into .bam or .sam. The installation of the aligners is as follows:

- Bowtie2: `conda install bowtie2`
- STAR: `conda install c bioconda star`
- MOSAIK: `conda install c bioconda mosaik`
- BWA: `conda install -c judowill bwa`

Gene Ontology

Gene Ontology file binaries (installation files) are located in the Programs directory of NaviSE files. The location of this file must be indicated when running NaviSE, so it is important to locate it in a known place (for instance, in the same directory where anaconda is installed).

Please, mind not to rename, cut or delete any file inside *Programs* directory. If so, NaviSE may crash in the middle of the run.

Installing Goatools (Gene Ontology)

Goatools requires both an installation and some minor fixes that are patched in a file that comes in *Programs* directory. In order to install Goatools, follow these steps.

- First, install goatools by typing `pip install goatools==0.6.5` in the command prompt.
- We will also install wget `conda install wget` in the command prompt.
- Now, we have to apply the patch. Locate a file named `goatools` in the *Gene_Ontology* directory. Copy this file and paste it in your anaconda installation directory: `XXX/anaconda3/lib/python3.5/sit` It will ask if you want to replace the file, say Yes.

Goatools works with third-party software, which is required as well:

- Install pyparsing by typing `easy_install pyparsing` in the command prompt.
- Install fisher by typing `easy_install fisher` in the command prompt.
- Then, install graphviz by typing `pip install graphviz` in the command prompt.
- Finally, install pydot2 typing `pip install pydot2`. pydot2 also requires to be patched, for which you only have to copy the `pydot.py` located in the *Gene_Ontology* directory, and paste it in `XXX/anaconda3/lib/python3.5/site-packages/` (say Yes if Linux ask you whether you want to replace the file).

Installing Genome viewer associated files

The programs required by the genome viewer to run are:

- pysam: `conda install -c bioconda pysam`
- pysamstats: `conda install -c bioconda pysamstats`

Installing HOMER

In order to run HOMER, two third-party software are required:

- Install weblogo by typing `conda install -c percyfall weblogo`
- Then, blatz by typing `conda install -c bioconda blat`

Now for HOMER installation, follow these steps:
In order to install HOMER, download this file from HOMER website and place it in the HOMER installation directory (make a directory named *HOMER* inside the directory where anaconda is located, for instance). Then, open the terminal and write `perl` and press Space ; drag the downloaded `configureHomer.pl` file into the prompt (the route to the file should appear), press Space and write `install`. The terminal should look like this (Fig. 5):

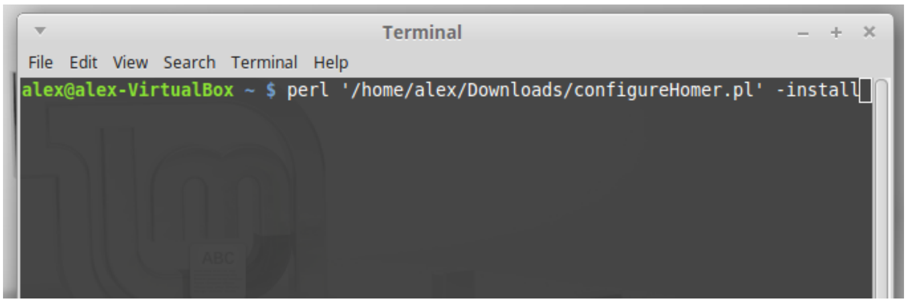

Figure 5: Homer installation.

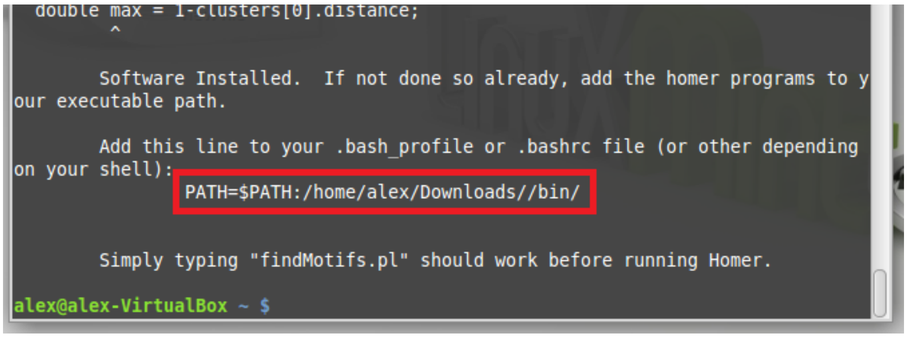

Figure 6: Homer PATH setting.

During the installation HOMER, will check whether all the third-party software was installed. If so, the installation continues (otherwise a message appears and HOMER waits 10 seconds for the user's response) and a message like that appears when the installation is finished (Fig. 6):

The message that appears in the red-squared area contains a path similar to the anaconda installation one, that has to be added to the PATH file (see Setting a path to a program), adding in into a new line.

Following the installation, we need to load information about the genome and the promoters of the animal. In order to install information about the genome, we follow the same steps used for homer installation and add the *name* of the genome. For instance, if we were interested in the version *hg38* from human, we should write:

```
perl PATH-TO-ConfigureHomer.pl -install hg38
```

HOMER should be able to recognize the genome and will install the information. This process may take some minutes, so it is recommendable to install other programs on the meantime.

Installing GSEA and beatifulsoup

For those two programs, the installation is quite simple: write these two lines at the command prompt:

- For GSEA: `conda install -c bioconda gseapy`
- For beautifulsoup: `conda install -c anaconda beautifulsoup4=4.5.1`

Installing programs for Enrichr/StringDB data extraction

In order to extract information from Enrichr and String, we need to have a version of Firefox $\leq$ 45.0.2. In order to check your firefox version write `firefox -v` in the prompt. The version should appear like this (Fig. 7):

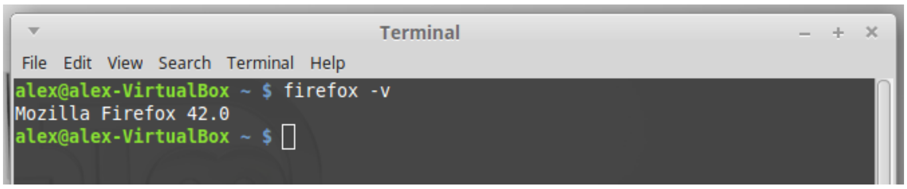

Figure 7: Firefox version.

Downgrading Firefox

If you need to downgrade firefox, follow these steps.

Warning: despite these steps are necessary (selenium does not work with Firefox versions more recent than 45) the following steps will uninstall your current Firefox, so make a security copy of all your bookmarks or history if necessary. Moreover, Firefox does not 'really' support downgrades, so it is possible that the new installation will induce some *errors*, like no icon or no direct access to firefox on program bar or Desktop (although you can just run Firefox typing 'firefox' in the command prompt).

We apologize for the inconveniences. Some solutions we propose on the meantime are installing chromium and use it as the default browser, or just reinstall firefox from the official page when you are done with the analysis. It is not *that* hard to do the downgrade when necessary anyways.

Note: theoretically, not installing the software for this part should not induce any errors (it will just not appear on the report), although the information obtained with this part is interesting and, therefore, worth the try.

- First, uninstall Firefox: `sudo apt-get remove firefox`
- Download the v45 release of Firefox:

  For 32-bit systems:

  `wget http://ftp.mozilla.org/pub/mozilla.org/firefox/releases/45.0/linux-i686/en-US/firefox-45.0.tar.bz2`

  For 64-bit systems (common):

  `wget http://ftp.mozilla.org/pub/mozilla.org/firefox/releases/45.0/linux-x86_64/en-US/firefox-45.0.tar.bz2`
- Extract the downloaded file: `tar xvjf firefox-45.0.tar.bz2`
- We move the file onto a *bridge* directory: `sudo mv firefox/ /opt/firefox3`
- We backup our current Firefox distribution: `sudo mv /usr/bin/firefox /usr/bin/firefox-old`
- Configure firefox: `sudo ln -s /opt/firefox3/firefox /usr/bin/firefox`

Now that firefox has been downgraded the following programs are required. As the previous programs, just type each line into the command prompt:

- Selenium: `pip install selenium`
- Selenium libraries: `sudo pip install -U selenium`
- pyvirtualdisplay (it allows to run the programs in the background): `pip install virtualdisplay`
- Xvft (required by pyvirtualdislay): `sudo apt-get install xvft python-pip`

Last but not least... setting NaviSE path

Although this last step is not necessary, we highly recommend doing it. By setting the path to NaviSE, in order to run NaviSE in subsequent times, it will only be required to write `python3.5 NaviSE.py` in the command prompt, instead of the full path of *NaviSE.py* location.

In order to set the path, the following line must be added to the bashrc file (see how to do it in Setting a path to a program):

`PATH=PATH$:XXX`
Where `XXX` is the directory where *NaviSE.py* file is located.

Automatic installation

We have recently developed a python file which allows easy NaviSE installation without going through the tedious step-by-step installation. In order to install NaviSE automatically, head to the directory of NaviSE and look for *NaviSE_installer.py* file. Then, open the command prompt (pressing Ctrl + Alt + T) and write `sudo python3 PATH-TO-FILE/NaviSE\_installer.py`, which should prompt the installation program. The command in the prompt should look like this (Fig. 8).

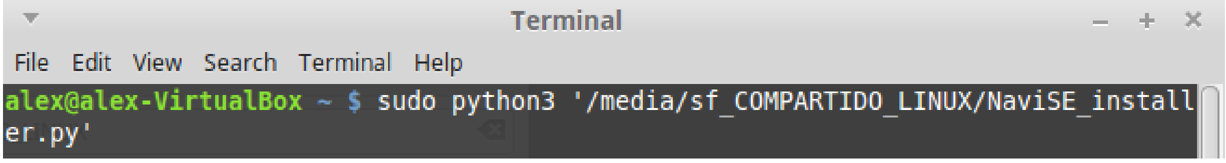

Figure 8: Installation prompts.

If the program does not run, it may yield two errors:

- Python3 is not installed in the system. You can install Python3 by typing in the console `sudo apt-get install python3`
- Python3 tkinter (python3-tk) is not installed in the system. You can install it by typing in the console `sudo apt-get install python3-tk`

In both cases, it will ask you to input a password. After you do it, all the basic programs will be installed and you will be able to run the installation program.

When running the installation software, a first blue screen appears which reminds you that Firefox will be downgraded (see Downgrading Firefox for further details); make sure you **CLOSE FIREFOX** before running the installation software and click on *I agree*. Then, a second prompt appears in which the directory where al the programs will be installed. Click on the '...' button and select the directory. Then, click on *Continue*. Both prompts appear on Fig. 9.

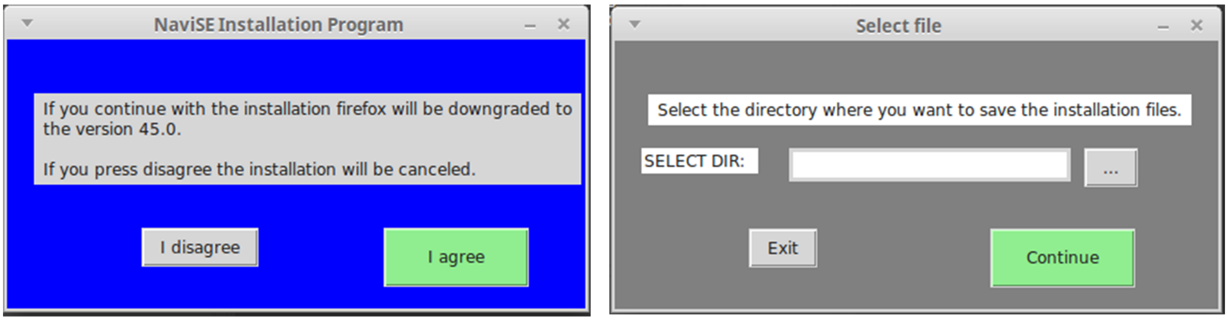

Figure 9: Installation prompts.

The installation may take a while (even more than an hour for some computers), so we recommend to read the manual thoroughly in the meantime to understand how to run NaviSE properly.

Warning: this installer checks the presence of HOMER and Anaconda installations, so as not to install them in other places. If anaconda is already installed **IN THE DIRECTORY OF THE INSTALLATION**, the installer will skip anaconda installation and will automatically install all the required dependencies through conda. If HOMER is already installed, please, install manually the hg38 and mm10 genomes if they have not already been installed. Install all the rest of the genomes which are not from human or mouse as well, if they are going to be used. Look at Installation of other genomes and Installing HOMER to see how to install a genome with HOMER.

NaviSE Genomes

NaviSE is programmed to allow the prediction of superenhancers based on any sort of genome, which is explained later.

Total implementation of NaviSE (functional chromosomal plots, Enrichr, GOEA, etc.) is only applied to hg38/19 in human and mm10/9 in mouse. The organisms in the following table include information about TF from HOMER and StringDB:

Name	Version	Scientific name	Common name
dm	3, 6	*Drosophila melanogaster*	Fruitfly
rn	4, 5	*Rattus norvergicus*	Rat
hg	18,19,38	*Homo sapiens*	Human
susScr	3	*Sus scrofa*	Pig
sacCer	2, 3	*Saccharomyces cerevisiae*	Yeast
rheMac	2, 3	*Macaca mulatta*	Rhesus macaque
tair	10	*Arabidopsis thaliana*	Arabidopsis thaliana
mm	8,9,10	*Mus musculus*	Mouse
galGal	4	*Gallus gallus*	Chicken
ce	6, 10	*Caenorhabditis elegans*	Caenorhabditis elegans
xenTro	2,3	*Xenopus tropicalis*	Xenopus tropicalis
danRer	7, 10	*Danio rerio*	Zebrafish
ci	2	*Ciona intestinalis*	Sea squirt
canFam	3	*Canis familiaris*	Dog
gorGor	3	*Gorilla gorilla*	Gorilla
panTro	4	*Pan troglodytes*	Chimpanzee

Finally, the use of any other genome will include the prediction and annotation of superenhancers, chromosomal plots, and statistical graphs. Apart from hg19/38 and mm10/9, any other genome must be installed in the system of files of NaviSE.

Installation of other genomes

In order to install other genomes, these are the steps to follow:

- If the genome appears in the table, in order to obtain information about HOMER TF, the genome must be installed in HOMER binaries. In order to install the genome, take the homer installation file `configureHomer.pl` and load it into the console. Then, install the genome by typing: `perl PATH/TO/ConfigureHomer.pl -install XXX` where `XXX` is the genome. This step is also explained in the section Installing HOMER.

- Downloading fasta assemblies and chrom sizes. Fasta assemblies are files with information of the genome of the organism. They are required to align the reads with Bowtie2 or other aligners. Chrom sizes is a file that contains information about the size of the chromosomes, which is used to create the chromosomal plot.
  In order to download these files: (1) Head to `http://hgdownload.soe.ucsc.edu/downloads.html` and click on the organism. (2) Choose the desired genome version.(3) It will redirect to an ftp where several files are located. Choose the file ending with .chrom.sizes and one which end in .fa.gz. This last file should also contain the name of the genome or something similar. Put the chrom sizes file into the *Chrom_sizes* directory at NaviSE files. The fasta assembly file is compressed (into the .gz file) so it must be extracted with a compressor like gzip or 7zip. The final file, `XXX.fa` must be renamed to `.fasta` and must contain the name of the genome. These steps are depicted in the figure 10

- Downloading gene files. Gene files are files which provide with information about genes and are required by NaviSE to annotate the superenhancers. In order to download the gen files, head to Biomart web page (1), `http://www.ensembl.org/biomart/martview/` and choose the organism from the database. Then, stablish some filters for genes, like genes with Entrez IDs (2). Then, select the attributes to be shown (3). Among the number of attributes, those that

are ticked must be chosen. Finally, download the CSV file (4). The file should be *mart_export.txt*. Rename the file with the genome (*XXX.txt*) and place in in the *Genes* dir at NaviSE files. These steps are depicted in the figure 11

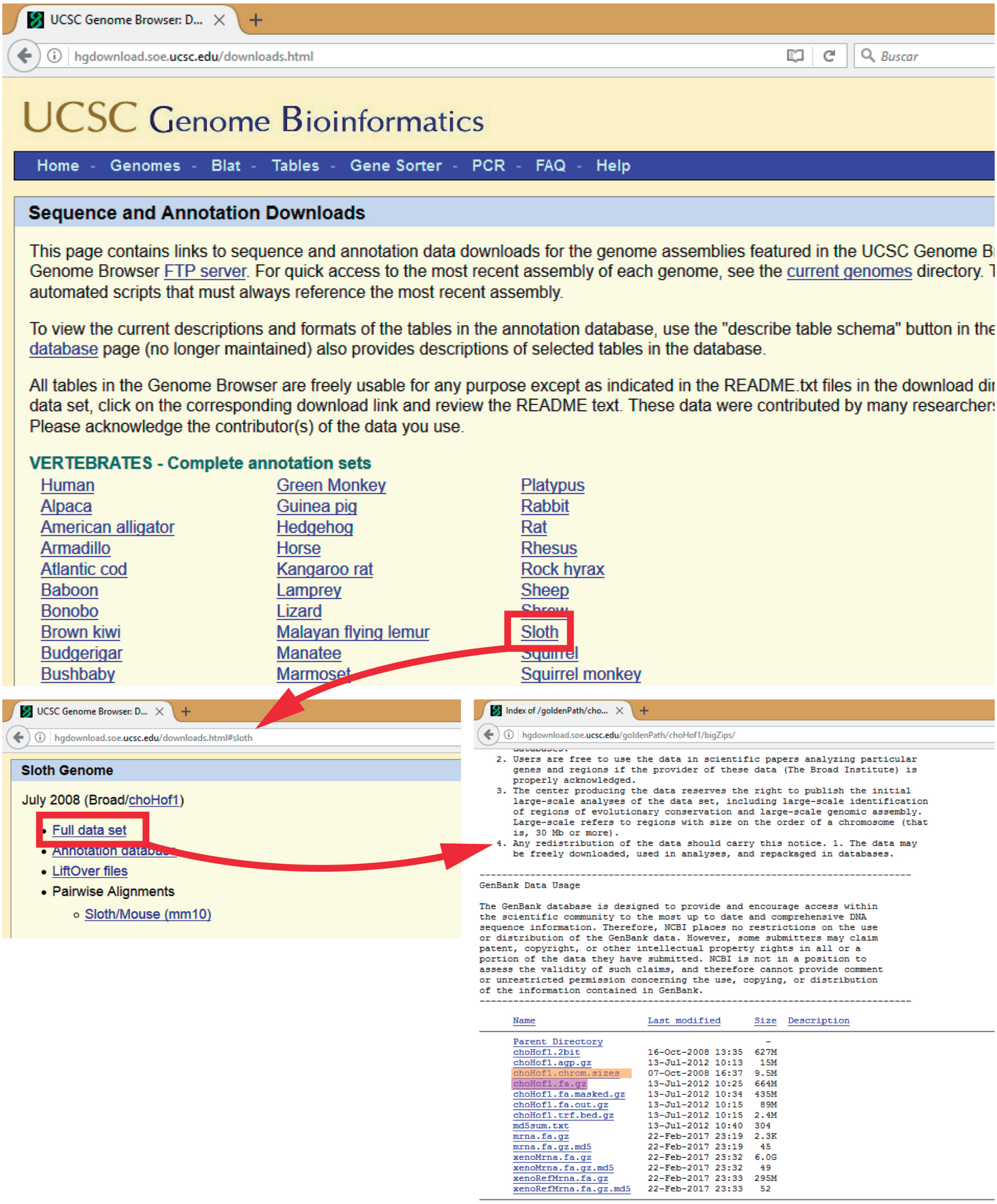

Figure 10: Download of files from UCSC

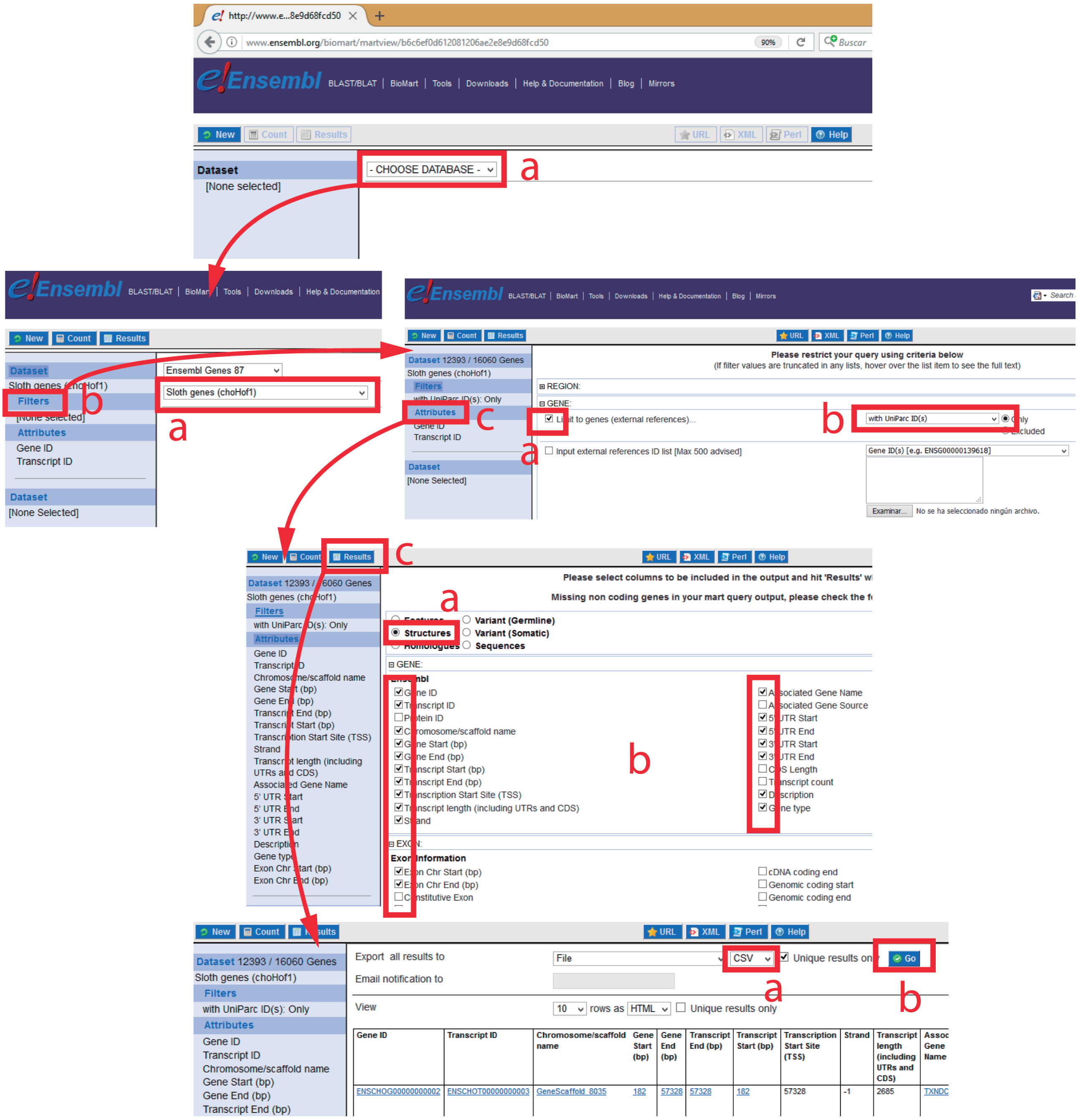

Figure 11: Download of files from Biomart

Commands

In order to run NaviSE, the python file `NaviSE.py` must be run in the terminal, adding all the required commands afterward. NaviSE requires python3.5, so the python file must be run with python. Hence, an example of a run of NaviSE should be like that:

```
python3.5 XXX/NaviSE.py [OPTIONS]
```

Where `XXX` is the path where *NaviSE.py* file is located. If you have automatically installed NaviSE or you have set the PATH to NaviSE, you only have to write:

```
python3.5 NaviSE.py [OPTIONS]
```

The commands allowed in user input are described below. The color scheme is the following:

(O/R) [-s][–long-format] (default-value) [input] : [Description]

(O/R) indicates if the argument is Required or is Optional. If a required argument is not inserted, it will throw an error. If an optional argument is not introduced, it will use the default value.[-s][–long-format] is the format of the variable to which you can assign your value. Remember that for [-s] a single hyphen at the start is required whereas for [–long-format] a double hyphen is required. (default-value) indicates which is the default value in case an optional variable is not declared. Lastly, [input] refers to the user input that will be assigned to that variable (sometimes an example of a value appears).

As for inputting, NaviSE will follow some recognition steps, so take this points into account so as not to get any error:

- If a path to a directory/file is introduced, the paths of the files must not contain any spaces. For instance, /path to file/file a.png should be corrected to /path_to_file/file_a.png. Also, it is recommended not to include symbols like (, ; ! ? % &), etc.
- In some cases, more than one option can be selected (like a gene list or several GSEA options). In that cases, each element must be separated by a **SPACE**, no commas, colons or semicolons. For instance, [a b c d] will be split to elements ['a', 'b', 'c', 'd'], whereas [a, b, c, d] will be split to ['a,', 'b,', 'c,', 'd'], or [a,b,c,d] will not be split because there are not spaces.

Warning: some of the programs referred in the commands may sound unknown. We describe them in detail in the Running NaviSE section.

Allowed commands are the following:

- (O) [-r][–root] (location of NaviSE.py) [XXX/NaviSE.py] : Directory where all NaviSE python files and other related files (logos for html, bowtie indexes, band files for chromosomal plots, etc. will be located.)
- (O) [-a][–program-root] (location of NaviSE.py) [XXX/Programs/] : Directory where ROSE, IGV and Gene Ontology directories are located. All directories must be located in the same directory.)

- (O) [-ch][–conda-homer] (location of NaviSE.py or home directory) [XXX/Programs/] : Directory where conda and homer files are located. By default it will search at /home directory as well as the directory where NaviSE.py is located, although it is recommended to include the exact location of anaconda and HOMER directories if there are many files at /home, since the search may take a while. There are several combinations of input:
  - If no directory is introduced, NaviSE will perform an automatic search. If it does not find anything, because files are not located at /home or NaviSE.py directory, or because conda might not be installed, please, locate the directories and include them at the command line. In order to search for conda or HOMER, write at the terminal, respectively, `which conda` and `which annotatePeaks.pl`.

    It will return something like `../anaconda3/bin/conda` and `.../HOMER/bin/annotatePeaks.pl`. Therefore, you should add to the command line the directories `../anaconda/bin/` and `../HOMER/bin/`, or `../anaconda/` and `../HOMER/`.
  - If homer and conda share the same directory, you can write the common directory, although we recommend to introduce both specific directories to save time.
  - If both directories are introduced, NaviSE will allocate the **first directory to conda** and the **second directory to HOMER**, not the other way round.
  - If HOMER is not installed and you do not know the directory, it is possible to write `None`, and NaviSE will look for conda at /home or at the location of NaviSE.py. However, we recommend writing the directory to conda to save time.
  - If conda directory is to be introduced but HOMER is not installed (because it is not going to be run), the way to introduce the command is `XXX None` where `XXX` is the directory to conda. In this way, NaviSE recognizes that, with `None`, HOMER is not installed, and will not spend time looking for it.
- (R) [-i][–input] [XXX/DIR_OF_CHIPSEQ_FILES/]: Directory where all the ChIP-seq files will be located. Whether one file or multiple files are analyzed at once, this directory must be created, as all the result files will be created in that directory.)
- (O) [-o][–output] (/root-of-bams/SUPERENHANCERS/cell-mark/) [XXX/NaviSE.py] : Directory where Superenhancer results will be located. If it is blank it will be automatically completed, if it is a name (does not contain /) a directory will be created in the input directory (directory where bam files are located), and if the name is a route, it will create the route and will set the files there. In order to create subdirectories in the input directory, write "../DIR1/DIR2/".
- (R) [-n][–file-name] [H3K27AC]: Corresponds to the name of the file. Our recommendation is to put the name of the transcription factor or histone mark that is being analyzed. If there is other information to add, like cell types or other *index* names, we recommend using the command [-m] to assign that parameter. If more than one replicate is analyzed, like [H3K27AC_1, H3K27AC_2, H3K27AC_3], write the common name of them (H3K27AC), and NaviSE will take charge of determining the replicates by itself. NaviSE also allows the combination of different marks/samples with logical operators. The combinations allowed are the following:
  - AND: it takes the region of superenhancers which appears in both marks, and takes the minimum value from the intersection.
  - OR: it takes the junction of both samples, that is, the signal which appears in A or in B; and takes the maximal signal of both samples in case of the intersection.

- +: similarly to OR, it takes the junction of both samples, although it performs the sum of the signal instead of the maximal signal of the intersection.
- NOT: it takes the peaks which appear only in the first signal.
- -: similar to NOT, but although it substracts the second signal from the first one. IF any pileup value is negative, it is converted to zero.
- XOR: it takes the signal that appears only in A or in B, but not in the intersection.
- SYM: similar to XOR, although the signal at intersection points may appear, as it performs the substraction of the signal, not the logical negation.

A scheme of the logical operators is depicted in Fig. 12.

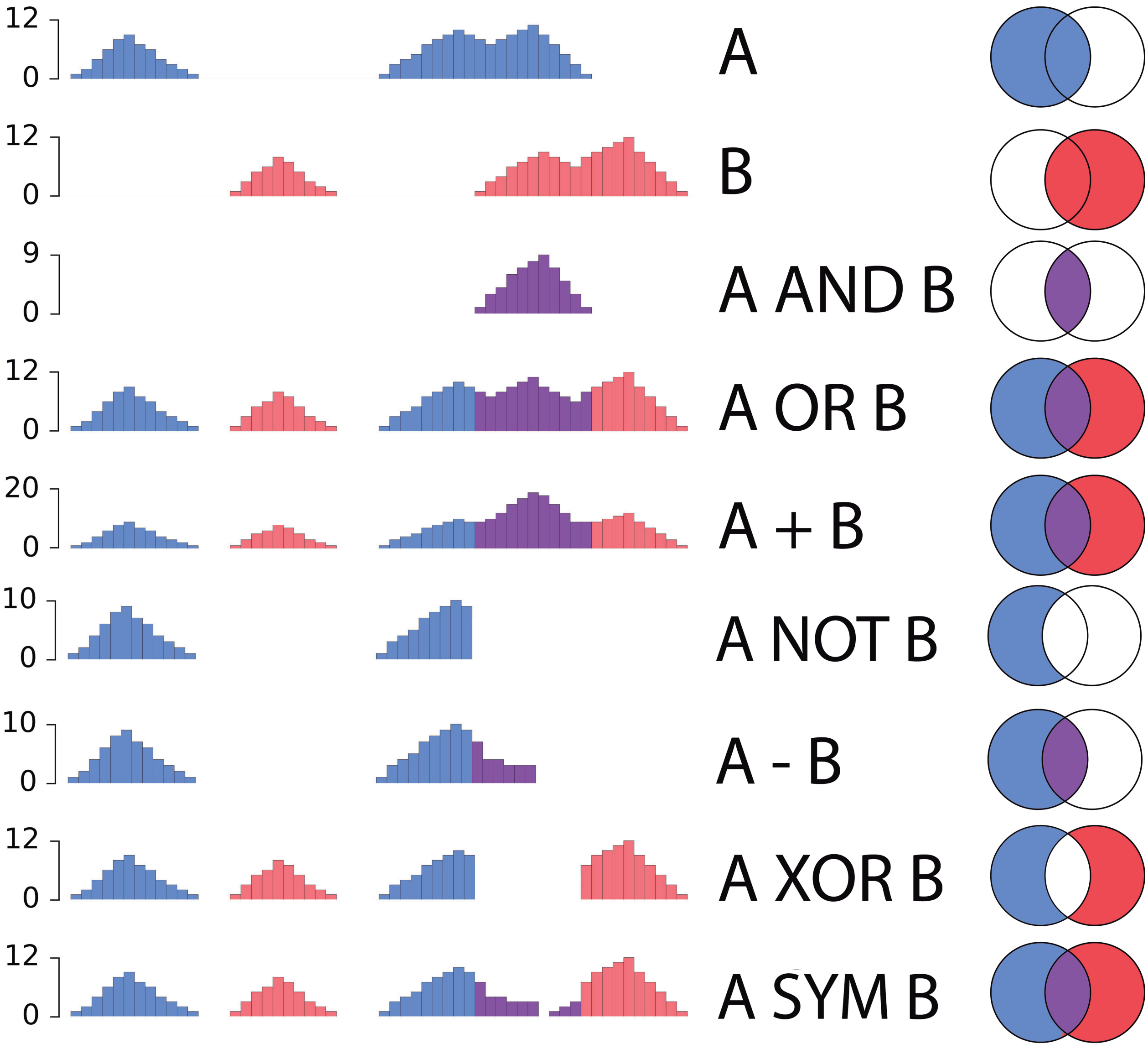

Figure 12: Logical operators

Logical operators work in pairs and sequentially, that is, if we write **A OR B NOT C**, NaviSE will first perform the junction between A and B, and then will remove the peaks from C.

- (O) [-m][–cell-name] (Nothing) [CD4 (A cell type, for instance)]: A secondary specifier for the filename.

  Combining both [-n] and [-m] parameters, NaviSE will recognize all the files, and will name all the subsequent files with the format 'M_N' (for example, CD4_H3K27AC). This name structure will be used throughout the process and will appear as such in the report. If no [-m] is assigned, the final name will only be the correspondent to [-n].

- (O) [-c][–control] (Nothing) [CD4_CTRL]: The name of the control file(s). If more than one replicate exists, use the root name of all replicates, like with [-n]. In this case, there is no [-m] secondary specifier. If any, it has to be manually separated from the main name by an underscore.
- (R) [-g][–genome] [hg38]: Input the genome of the organism. Supported genomes: hg38, hg19, hg18 (might cause some problems) for human; mm10, mm9 for mouse.)
- (O) [-s][–stitching-distance] (12500) [12500]: The stitching distance ROSE will use for the stitching of MACS peaks. These stitched regions will be used later on to calculate the number of reads and will be ranked, resulting in the list of superenhancers.
- (O) [-d][–tss-distance] (2500) [2500]: The distance from the center of the peaks to the TSS ROSE will filter out of the analysis.
- (O) [-x][–macs-threshold] (MAX) [PER25]: The threshold for peak selection after peaks have been identified by MACS. There are several options:
  - [MAX]: NaviSE calculates the maximum bin from the histogram of the distribution of values (the kind of value is determined with the [-y] option). The number of optimal bins in order to select the maximum bin (which will stablish the threshold) is selected according to the Freedman-Diaconis rule.

    $$h = 2\frac{IQR}{n^{1/3}}$$

    The bin width is proportional to the interquartile range (IQR) and inversely proportional to the cube root of the size. Can be too conservative for small datasets, but is quite good for large datasets. The IQR is very robust to outliers.
  - [PERXX]:XX is a value between 1 and 99. NaviSE calculates this value according to the percentile of values; that is, if [PER99], NaviSE will select the highest 1% of the peaks.
  - [##]: ## is a number above zero. NaviSE will select the values above the threshold.
- (O) [-y][–macs-choice] (1) [3]: The statistic value upon which NaviSE will apply the threshold. Only accepts numerical values:

  0 -log10(p-val)

  1 -log10(q-val) (recommended)

  2 Pileup: Pileup height at peak summit.

  3 Fold enrichment: Fold enrichment for the peak summit against random/control background.
- (O) [-t][–time] (T) [F]: Makes a tabular (csv) report of how long has each process of the program taken.

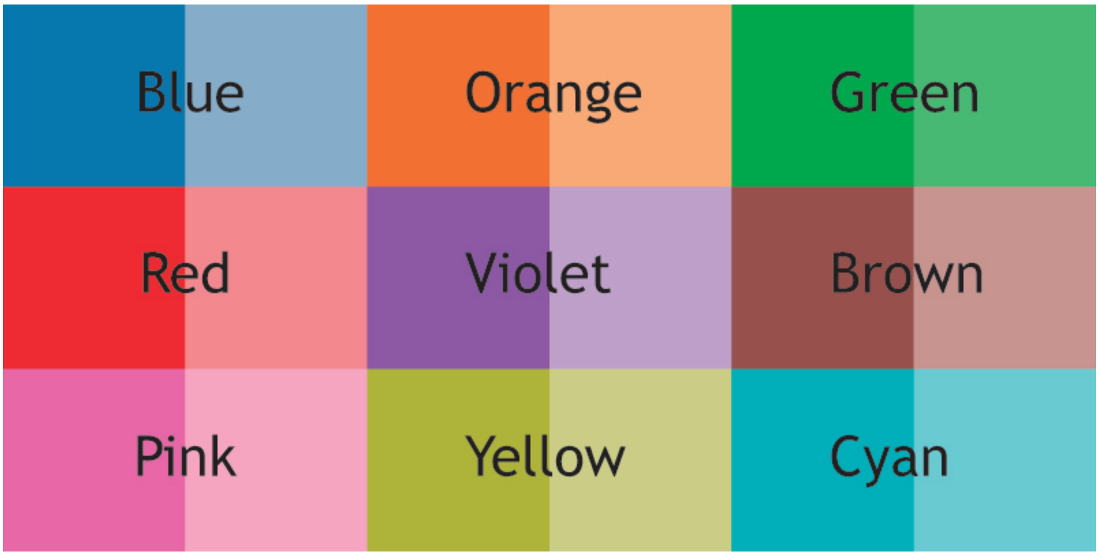

Figure 13: List of colors for coloring.

- (O) [-P][–processors] (auto) [4]: The number of processors NaviSE will use for process multiprocessing. When [auto], NaviSE calculates the optimal number of processors according to how much memory each process consumes, using a maximum of the 80%.

- (O) [-M][–mode] (1) [0]: Mode in which NaviSE will run its commands.

  0 : NaviSE will run the basic commands (obtain superenhancer files, basic statistical graphs and chromosome plots, a few of the superenhancer snapshots and the html report).

  1 : NaviSE will run everything.

  2 : NaviSE will take some snaps and will do all graphs, but will exclude Enrichr, StringDB, HOMER and GSEA.'

> Note: modes [0] and [2] are for computers that do not contain almost any processing capacity. However, if the computer has more than 4 or 6 processors, we recommend using the [1] mode, since even with little processors, the amount of time saved will be considerable for the amount of information this mode provides.

- (O) [-N][–samples] (30) [47]: The number of elements (e.g. number of snaps in mode 2, number of bars in the graph of GOEA, etc.) NaviSE will represent in the report.

- (O) [-C][–colors] (Blue Red Green) [Pink Orange]: List of color pairs that will be used for graph making. If less than three colors are chosen, NaviSE will choose the remaining at random. The list of colors is the following (Fig. 13):

- (O) [-D][–dpi] (450) [790]: The number of dots per inch of the graphs. We recommend a value around 300 or 400. A value higher than 700/800 or below 150 dpi is not recommended.

- (O) [-G][–gsea-cutoff] (All) [PER75 SE 390 All]: The choice (or list of choices) of superenhancers + typical enhancers that NaviSE will use for GSEA.

  – [All/None]: GSEA will be run with all superenhancers and typical enhancers.

- [PERXX]:XX is a value between 1 and 99. NaviSE calculates this value according to the percentile of values; that is, if [PER99], NaviSE will select the highest 1% of the peaks; and will run GSEA with the superenhancers + typical enhancers within that range.
- [##]: ## is a number above zero. NaviSE will select the lines corresponding to superenhacers or typical enhancers threshold.
- [SE/ONLYSE]: GSEA will be run only with suerenhancers.

Note: We know that the number of superenhancers or typical enhancers varies a lot between samples and we don't know this beforehand, so using a number *per se* or the [PERXX] choice might be risky. Still, it is interesting to guess which this value might be (after all, if it is wrong NaviSE will pop this value out of the list), so you can input several values and select the results that best fit to your analysis.
On the other hand, it is not recommendable to use the [SE] option, as most of the times no signatures of GSEA are matched to this sample.
Taking these recommendations into account, the best choice is to make a range of thresholds, trying to exclude the highest number of typical enhancers as possible, but without reducing the number of matches too much.

- (O) [-S][–signatures] (All) [h c1 c3 c6]: The gene sets corresponding to signatures of MSigDB. Currently, no custom gene sets can be added. Options: "All" (all the signatures), "h" and "cX" being X from 1 to 7. These are the gene set category that each signature includes (according to the MsigDB page):

  h hallmark gene sets: are coherently expressed signatures derived by aggregating many MSigDB gene sets to represent well-defined biological states or processes.

  c1 positional gene sets: for each human chromosome and cytogenetic band.

  c2 curated gene sets: from online pathway databases, publications in PubMed, and knowledge of domain experts.

  c3 motif gene sets: based on conserved cis-regulatory motifs from a comparative analysis of the human, mouse, rat, and dog genomes.

  c4 computational gene sets: defined by mining large collections of cancer-oriented microarray data.

  c5 GO gene sets: consist of genes annotated by the same GO terms.

  c6 oncogenic signatures: defined directly from microarray gene expression data from cancer gene perturbations.

  c7 immunologic signatures: defined directly from microarray gene expression data from immunologic studies.

Warning: The signatures from MsigDB correspond only to human genes. Still, trying GSEA with mouse samples might lead to interesting results as well.

- (O) [-L][–gene-list] (Nothing) [SOX2 POU5F1 ACTN1 FN1 PI3K]: A list of genes determined by the user (may refer to genes they are interested in). NaviSE will recognize those genes and will mark them in bold in the chromosomal plots or in tables if they appear.

- (O) [-A][–aligner] (BOWTIE) [MOSAIK]: Choice of the aligner for the program to align the fastq files to sam files.

- (O) [-Z][–with-subpeaks] (True) [True]: Add the subpeak locations at the Genome Viewer graph. This feature is explained later.

Running NaviSE

For those users who are interested in knowing the details and the process NaviSE goes through, here is a detailed explanation of each process:

a) **Sra to bam:** At this very first step, NaviSE recognizes files that contain the introduced filename and determines their format, as well as control files. Allowed **dataformats** are `.sra`, `.fastq`, `.sam`, `.bam` and `.bed`. After determining the formats, NaviSE will transform a superior format (`.sra`, `.fastq` or `.sam`) into `.bam`. If there are more than one formats, NaviSE will make a decision upon the number of files of each type. For instance, if the number of superior files is higher than the inferior ones, NaviSE interprets this as if the transformation was not complete, so it transforms all the superior files again.

   Alignments are performed by bowtie2. Bowtie2 needs to create some index files for alignment of reads with the genome, those index files will be located at NaviSE directory. The first time NaviSE is run, bowtie2 will create these files, which may take around 3 hours. However, this is a once-in-a-lifetime process, since at the subsequent runs NaviSE will detect these files and bowtie2 will not need to generate new ones. However, if this files are moved, renamed or deleted bowtie2 will need to remake those files (it may take less time since some files are stored internally), so take that in mind. Also, if another genome is used, bowtie2 will create other files for that genome version.

   If other aligners are used, the procedure is the same: if it is the first time that the aligner is used for one genome, the NaviSE will call the aligner to generate the index files and then it will align the reads using those index files.

   Warning: Despite NaviSE transforming successfully .bed format to .bam, we recommend using a format that has not been aligned, such as .sra or .fastq, in order to make sure the processed files correspond to the correct version of the genome.

b) **FastQC:** FastQC is a program that performs a quality analysis of `.fastq` files (Fig. 14). Then, it creates a report in which several quality parameters are included, such as per base quality, GC content, or presence of adapters.

c) **Combination of bams:** If there is more than one replicate or control, NaviSE will combine all the `.bam` files into one, and use this file for the analysis. If subsequent analysis are performed with that file and NaviSE detects this combined file, NaviSE will not combine the files again. Therefore, if any modification is performed to any of the original files, mind deleting the combined file as well.

d) **MACS:** MACS is a software that calculates peaks from bam files. Those peaks indicate the presence of a histone mark or the binding of the protein/transcription factor ChIP-seq analysis is performed with. If a control is introduced, MACS will use the information from control signal to calculate the peaks from the sample. Instead, if no control is introduced, MACS will use a precalculated background.

   Once MACS has determined the peaks from the sample, peak files are processed for Superenhancer prediction by ROSE. In this processing, peak values below a threshold determined by [-x] and [-y] are excluded.

e) **SE prediction:** NaviSE uses the algorithm developed by Young to predict the presence of superenhancers in a sample (Fig. 15). The algorithm is a stitching algorithm, that is, given a

f

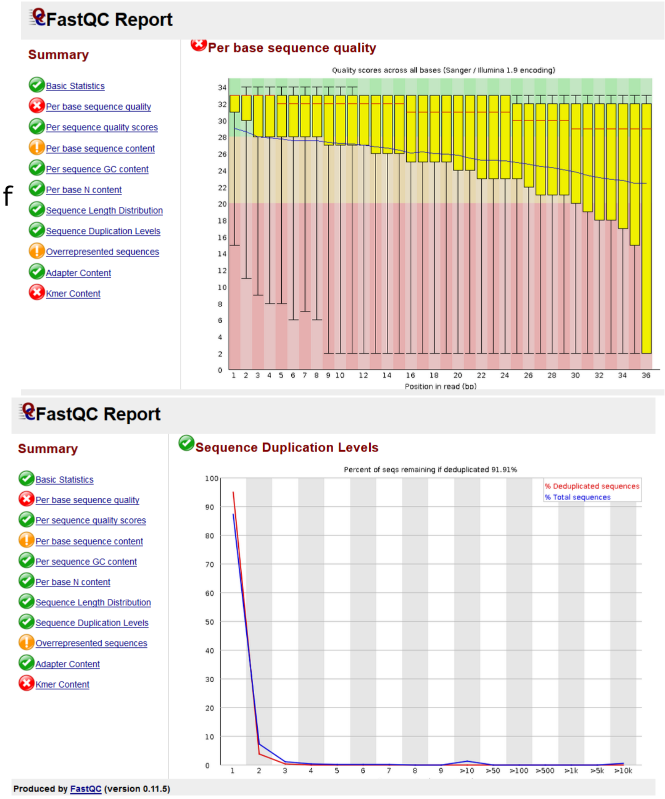

Figure 14: FastQC analysis

file that contains the location of enhancers (in our case, the processed peak file from MACS), NaviSE "stitches" those enhancers separated less than a threshold value. Out of these "stitched" enhancers, NaviSE ranks them by the number of reads that fall within that region. Finally, NaviSE establishes a cutoff, so the "stitched" enhancers falling within that range will be considered as Superenhancers. (Image from Sebastian Pott & Jason D Lieb. What are super-enhancers. Nature Genetics 47,812(2015). doi:10.1038/ng.3167)

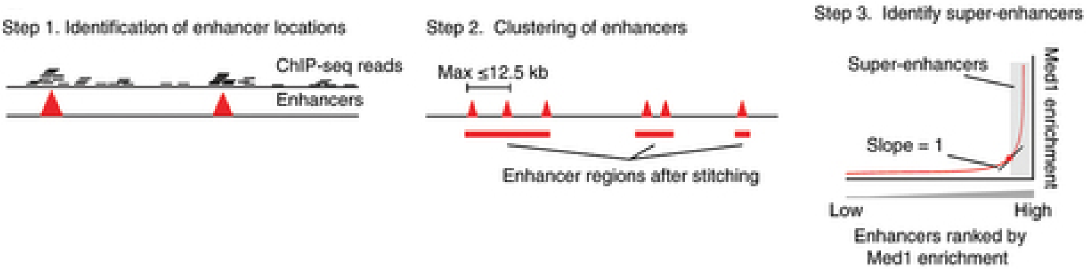

Figure 15: SE prediction algorithm.

f) **Gene annotation:** once the superenhancer locations are determined, each superenhancer is assigned a gene by proximity (independent of the strand of the gene). The following columns are included:

- Overlapping Genes: indicates genes that are overlapped with the superenhancer.
- Proximal Genes: genes that are next to the superenhancer but are not overlapped, and whose TSS are found at less than 250 kb from the superenhancer.

- Closest Gene: the closest gene to the superenhancer.
- Gene Type: the category to the gene corresponds (ncRNA, protein-coding, snRNA, etc.)
- Gene Description: brief description of the closest gene.

g) **Subpeak annotation:** in this subsection, further columns are created which expand information about superenhancers by adding data corresponding to the MACS peaks within each superenhancer. This information is contained in several columns:

- Number of subpeaks: number of subpeaks that each superenhancer has.
- Loci and TSS locations: locations of the subpeaks and the distance from each subpeak to the TSS of the Closest Gene.
- SE Status, INS, OUTS, Percentage OUTS and Enhancer Type: SE Status, INS and OUTS relate to those subpeaks that fall within the range of the TSS-threshold determined by [-d]. From these values, a percentage of OUTS is calculated and, from this value a Enhancer Type is assigned, among 3 possibilities: *Mixed* (it contains OUT and IN subpeaks), *Pure* (it contains only OUT peaks) and *Only TSS* (it contains only IN peaks).

h) **Snaps:** NaviSE takes two snapshots of each superenhancer, one of them called *near* and the other one called *far*. Both options extend the locus begin and end following this equation:

$$x_0^{'},\ x_f^{'} = x_0 - \frac{(x_f - x_0)\cdot(k-1)}{2},\ x_f + \frac{(x_f - x_0)\cdot(k-1)}{2} \qquad , \qquad x_f > x_0$$

where k is 1.2 for *near* and $\frac{400}{(x_f - x_0)^{0.34}}$ for *far*. If more than one sample is plotted, then an additional *Preview* snap is added, which contains the first sample; and which is added to the html final report (Fig. 16).

i) **HOMER motif finding:** HOMER (Hypergeometric Optimization of Motif EnRichment) is a suite of tools for Motif Discovery and NGS analysis. NaviSE uses HOMER in order to identify motifs of regulatory elements (mainly transcription factors) that are specifically enriched in the loci of superenhancers, relative to the loci of typical enhancers (which will be used as background). As a result, HOMER writes a list of motifs enriched in superenhancers and another list of *de novo* motifs, that is, a novel algorithm developed by HOMER which finds motifs for which their binding element is unknown, and tries to determine which is this element. Results of HOMER are explained in HOMER analysis.

j) **Gene Ontology Enrichment Analysis (GOEA):** GOEA is an analysis performed over the set of superenhancer genes. A GOEA results in a list of Gene Ontologies, that is, sets of genes belonging to a certain metabolic/cellular pathway, to which the set of superenhancer genes is enriched in contrast to the background gene set. A result from a GOEA is further explained in GOEA.

k) **Enrichr and StringDB results:** In this subsection, we extract data submitted to Enrichr and StringDB webpages. Enrichr comprises a number of databases, such as Human/Mouse Gene Atlas, ChEA/ENCODE consensus TF from ChIP-X, TRANSFAC and JASPAR PWMs, or Reactome/Wikipathways/KEGG pathways. StringDB is a protein-protein interaction (PPI) database which stablishes PPI networks, based on literature-determined interactions or predicted interactions. In both cases, NaviSE submits the superenhancer set and collects and processes that information. Results from Enrichr in Enrichr results and from StringDB in StringDB results. Finally, all the raw data from Enrichr is processed for an easier interpretation for the user, or filtered (selects organism-specific results).

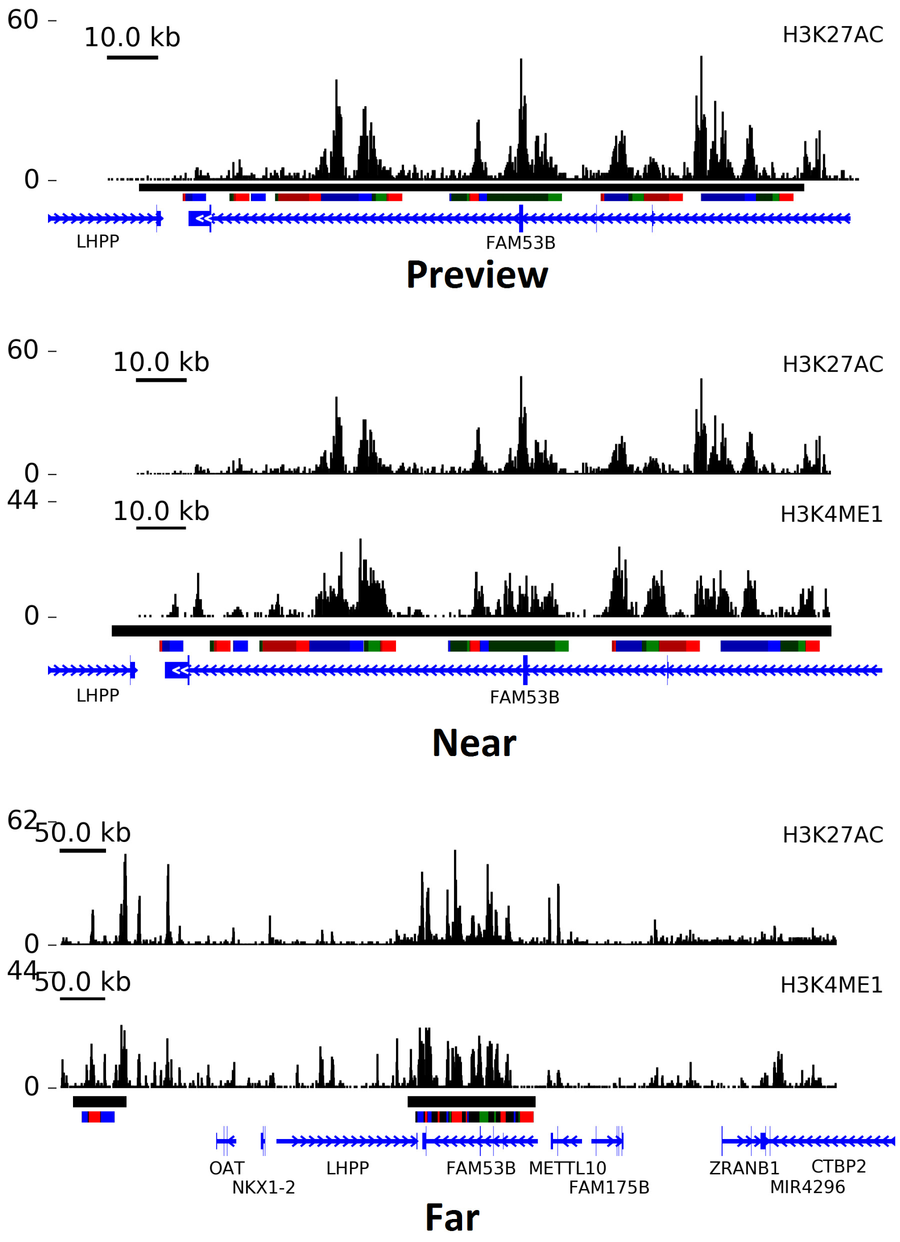

Figure 16: Genome Viewer sample at preview, near and far distances.

l) **Graphs and chromosomal plots:** The graphs production comprises a number representations about statistical values or properties shown before. These graphs will be explained in depth in NaviSE Graphs. They also include barplots for easier interpretation of GSEA or Enrichr results (explained in their correspondent sections).

As for chromosomal plots, which are further explained in Chromosomal plots, they are representations of superenhancer loci located in a karyotype. These plots allow users to make a first impression on how near or far two superenhancers are apart, or if a chromosome is depleted or enriched in superenhancers. In one NaviSE run three plots are generated:

- Simple plot: it just shows the loci location and the chromosome enrichment/depletion.
- Rank plot: loci are colored according to their rank. Several percentiles are represented,

so superenhnacers falling within a percentile will be colored with its corresponding color.

- Closeness plot: this plot represents the range of proximity between superenhancers (< 5 Mb for instance) which will be colored with its corresponding color. This plot is increadibly useful to tell apart clusters of superenhancers, which may look like one superenhancer. For a number of superenhancers within a chromosome, $x_1, x_2, \cdots, x_{a-1}, x_a, x_{a+1}, \cdots, x_{f-1}, x_f$, their distance is determined by the following formula:

$$d = \begin{cases} x_2 - x_1 & \text{for } x_1 \\ \min(x_{a+1} - x_a, x_a - x_{a-1}) & \text{for } x_a \\ x_f - x_{f-1} & \text{for } x_f \end{cases}$$

In all the plots a p-value for a enrichment score is calculated, which determines whether a chromosome is enriched (⅄ for $p < 0.05$ and ⅄⅄ for $p < 0.01$) or depleted (Υ for $p < 0.05$ and ΥΥ for $p < 0.01$). This p-value is calculated by a binomial approximation of the hypergeometric distribution, where N is the number of genes in the whole genome, K is the number of superenhancers in all chromosomes, n is the number of genes in a chromosome and k is the number of suerenhancers in a chromosome. This hypergeometric distribution is approximated to a binomial distribution ($h(k; K, n, N) \rightarrow b(k; K, p) \quad ; \quad p = \frac{n}{N}$), so the p-value for depletion is the cumulative distribution function for this binomial approximation and the p-value for enrichment is survival function.

m) **GSEA (Gene Set Enrichment Analysis):** GSEA is an analysis similar to GOEA, is a computational method that determines whether an a priori defined set of genes shows statistically significant, concordant differences between two biological states. In our case, the defined gene sets are the signatures from MsigDB (explained in signatures), and the two biological states are the superenhancers and the typical enhancers.

In our case, a similar approach is followed: both superenhancers and typical enhancers are ranked by their signal, the user filters out as many lines of these rank as it is stablished with the parameter [-G], and those genes that match the genes from a gene set from a signature are marked as positive. Then, positive matches are given a score according to their position in the list and a GSEA plot is drawn, which shows a curve that represents how "fitted" the ranking of genes is to the gene set. Further values representing the overall score of this "fitness" are also calculated.

GSEA results will be further analyzed in GSEA results. For further information about gsea, we recommend reading the following article.

n) **Writing HTML report, deleting files and writing the timetable:** This is the last part of the analysis. NaviSE gathers all the information into a user-friendly html interface through which the user can navigate and access all the aforementioned information, and which is discussed in detail with an example in NaviSE output. Obviously, all the information (tables, graphs,...) will be available in their respective files and directories in case the user wants to publish the figures or extract some data.

Finally, NaviSE removes some intermediate files which have no relevant information for the user; and creates a timetable (csv) in which the time taken for each part is shown.

Warning: Some of the processes that the timetable shows are not mentioned in this section, have a different name or belong to more than one section. However, the information is relevant and the user should have no problem in recognizing each process of the table.
If the timetable (which should appear in **FILES/timetable-DATE.csv**) is not present, it might be due to an error during processing. Also, if not all the subprocesses are present, this is due to an error or, simply, because that process was not run (because it was run before or because the selected mode restricts the process).

Parallelization of NaviSE

One of the min characteristics of NaviSE is its parallelization process, which considerably reduces the processing time. Currently, NaviSE parallelizes the most consuming processes, like e), f), g), h) and m), as well as minor processes such as a), c), d), in which the parallelization process is notorious at cases with multiple samples.
NaviSE determines the optimal number of processes, k, compatible with the computer resources. Such resources are the parallel processing capability of the computer measured as the number of cores, C, and the total main memory, M in GB. NaviSE optimizes automatically, for each processing task i, the number of processes, k_i:

$$k_i = \min\left(C, C_u, \lfloor M/m_i \rfloor, l_i\right) \tag{1}$$

where C_u is the maximum number of cores reserved by the user to run NaviSE, m_i is the memory, measured in gigabytes (GB), needed to run one process in task i, $\lfloor \, \rfloor$ is the floor operator and l_i is the cardinal of $D_i = \{d_1, d_2, \cdots, d_m\}$ which is the set of *chunks* of distributed elements to be processed in task i. If $l_i > k_i$, the first k_i chunks are distributed to k_i cores. The distribution of information (SE peak distribution profiles, number of gene sets for GSEA, chromosomes for Superenhancer prediction) to be parallelised is based on a cyclic algorithm, implemented in Python. For the ordered set $S_i = \{s_1, s_2, \cdots, s_n\}$ of information elements, the set $P_i = \{1, \cdots, k_i\}$ of processes and for the set D_i (chromosomes, gene sets, positions on a list) to be distributed across processors, we define D_{pi} as the *chunk* of the task i that is assigned to each processor p:

$$D_{pi} = \{d_j \mid \forall d \in D_i, p \in P_i, j \in \{1, \cdots, l_i\},\ \ j \bmod k_i = p\} \tag{2}$$

where mod is the module operator. Once the *chunk* D_{pi} is constructed, the subset of information elements $S_{D_{pi}} \subset S_i$ will be defined depending on the type of process which is being parallelised.
The list of parallelised tasks is $i = \{\text{STIT}, \text{SNAP}, \text{GSEA}, \text{HOMER}\}$. In the case of SE prediction (STIT), the input table with peak coordinates from MACS (S_{STIT}) is divided in k_{STIT} files, calculated with Equation 1, with $m_{\text{STIT}} = 2$ GBs. Here, $D_{p,\text{STIT}}$ represents the groups of chromosomes that will be processed in each $p \in P$, and $S_{D_{p,\text{STIT}}}$ is the *chunk* of $s \in S_{\text{STIT}}$ elements which share the same chromosome from each group of chromosomes from D_p. In this case, $D_{\text{STIT}} = \{Y, 22, \cdots, X, \cdots 2, 1\}$ (for human), i.e., the chromosomes are arranged in increasing length order, so that the distribution of $D_{p,\text{STIT}}$ is balanced across processors. For a better understanding of the process, an example is developed in Figure 17.
In the case of SE signal profile snapshot parallelisation, $S_{\text{SNAP}} \equiv D_{\text{SNAP}}$, is the set of SE *loci*. Hence $D_{p,\text{SNAP}}$ contains all the *loci* that fulfill Equation 2, based on k_{SNAP} with $m_{\text{SNAP}} = 2$ GBs.
In the case of GSEA parallelisation, S_{GSEA} is the set of genes ranked by SE score and D_{GSEA} is the set of combinations (GSEA signatures $\times$ GSEA cutoffs). Therefore, $D_{p,\text{GSEA}}$ contains all the combinations that fulfill the Equation 2, based on k_{GSEA} with $m_{\text{GSEA}} = 2$ GBs.
The parallelisation of all these cases has been implemented with the *multiprocessing* module of Python. In the case of HOMER parallelisation, we took advantage of the HOMER parallelisation capabilities already implemented in HOMER, with the number of processes k_{HOMER}, optimized by Equation 1, with $m_{\text{HOMER}} = 2$ GBs.

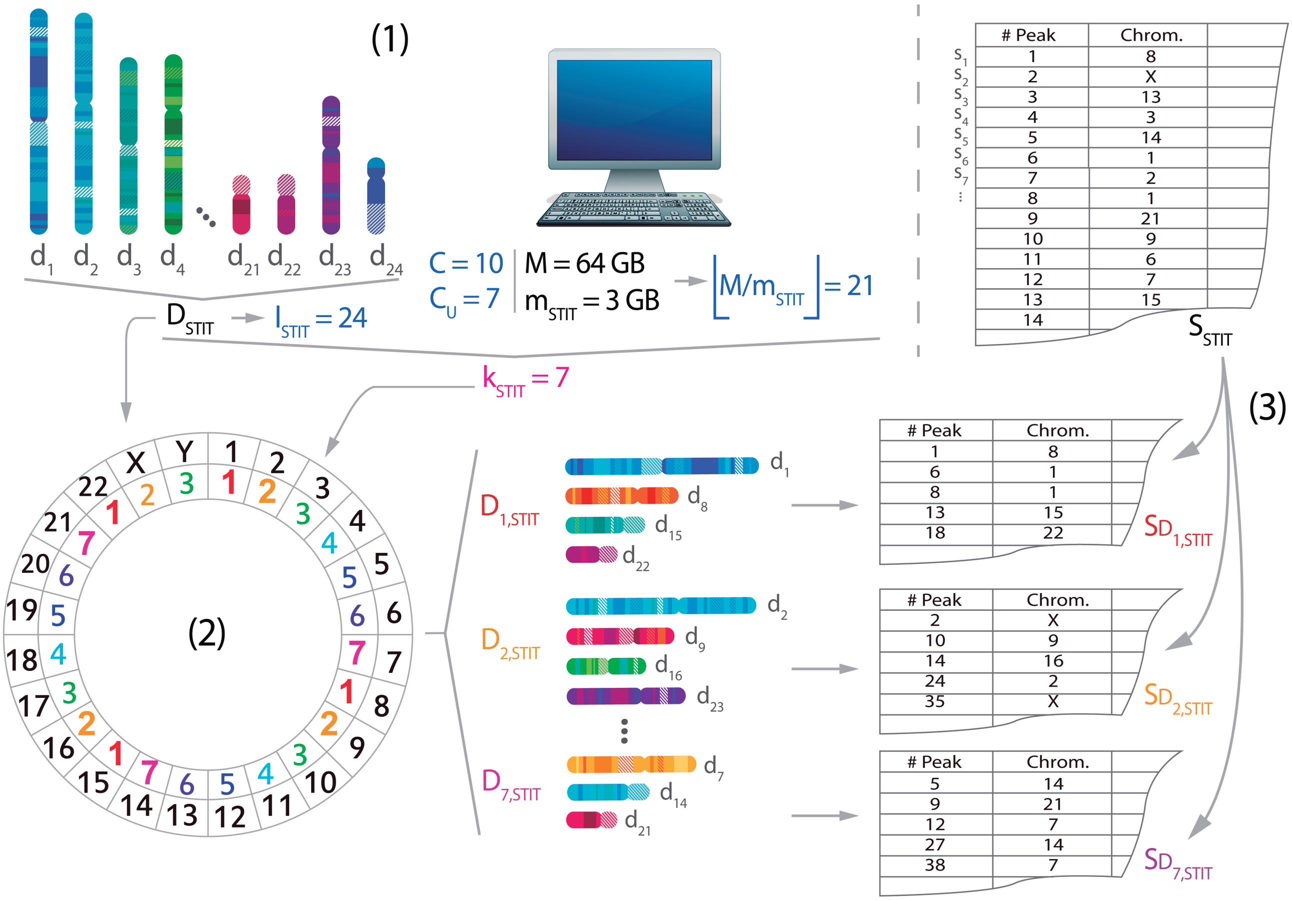

Figure 17: **Scheme of parallelisation of SE predicton.** (1) Determination of the number of processes (k_{STIT}) based on Equation 1, for which the number of cores (C) is 10, the maximum number of cores allocated (C_u) is 7, the memory of the computer (M) is 64 GB, the memory allocated to ROSE (m_{STIT}) is 3 GB and the cardinal (l_{STIT}) of the set of chromosomes ($D_{\text{STIT}} = \{d_1 = 1, d_2 = 2, \cdots, d_{22} = 22, d_{23} = X, d_{24} = Y\}$) is 24. The calculated value is $k_{\text{STIT}} = C_u = 7$. (2) Construction of *chunks* based on Equation 2. Since $k_{\text{STIT}} = 7$, the set of chromosomes D_{STIT} is divided into 7 subsets or *chunks*: $D_{1,\text{STIT}} = \{d_1, d_8, d_{15}, d_{22}\}$; $D_{2,\text{STIT}} = \{d_2, d_9, d_{16}, d_{23}\}$; $\cdots$; $D_{6,\text{STIT}} = \{d_6, d_{13}, d_{20}\}$ and $D_{7,\text{STIT}} = \{d_7, d_{14}, d_{21}\}$. (3) Assignation of information elements. In the case of ROSE, assigned elements are MACS peaks (inferred as enhancers). After the assignation of the subsets $D_{1,\text{STIT}}$, $D_{2,\text{STIT}}$, etc., the set of MACS peaks, $S_{\text{STIT}} = \{s_1, s_2, \cdots\}$ is divided into 7 subsets of elements, $S_{D_{1,\text{STIT}}} = \{s_1, s_6, s_8, \cdots\}$, $S_{D_{2,\text{STIT}}} = \{s_2, s_{10}, s_{14}, \cdots\}$, $\cdots$, $S_{D_{7,\text{STIT}}} = \{s_5, s_9, s_{12}, \cdots\}$. Finally, each subset of elements is simultaneoulsy processed by ROSE, all the 7 subsets of stitched enhancers are combined into one file and the SE rank is performed.

NaviSE output

NaviSE outputs a huge amount of heterogeneous data, which is explained thoroughly in this section. We will use an example run of human embrionic stem cells with the H3K27Ac histone mark.

The following commands were introduced in the command prompt:

```
python3.5 /media/labcombio1/8TB-HD/Alex/Programs/NaviSE/NaviSE.py
-a /media/labcombio1/8TB-HD/Alex/Programs/ -i /media/labcombio1/8TB-HD/Alex/hESC/
```

```
-n H3K27AC -m hESC -c CTRL -g hg38 -s 12500 -d 2500 -x MAX -y 1
-t T -p auto -M 5 -N 35 -C 'Blue Red Green' -D 450
-G 'se per80 all' -S 'h c1 c2 c3 c4 c5'
-L 'POU5F1 OCT4 NANOG SOX2 KLF4 ESRRB BRD4 PRDM14 SMAD3 TCF3 ZMYND8 RNU2-1'
```

The main interface of NaviSE consists of a top navigation bar showing the different subcommands; and also a left navigation bar (sidebar) which contains the different subsections of each subcommand from the top navigation bar.

Main page

The main pages, which contains NaviSE logo on the top navigation bar, contains a small table indicating main characteristics, such as the sample name, cell name or number of superenhancers (Fig. 18). On the other hand, the sidebar includes all the chromosomal plots.

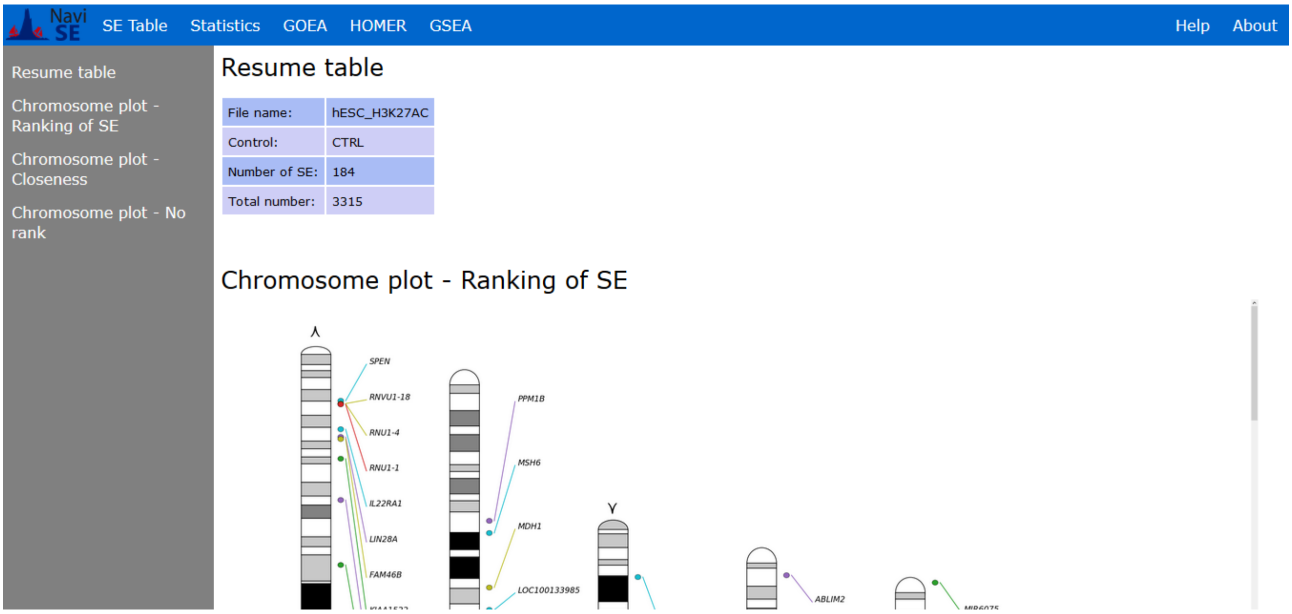

Figure 18: Main window.

The user can click on each name of the chromosomal plot, which will redirect to the correspondent superenhancer at the SE table section (Fig. 19).
Here is an example of some chromosomes from the chromosomal plot defined by closeness (Fig. 20):

SuperEnhancer table

SE table includes two subsections: *Complete table of SE*, which can be found at the **SUPERENHANCERS/ SUPER_CELL_NAME-CONTROL/FILES/Annotated_SE_table.csv** file and *Table of SE*, which can be found at the same file. The complete table contains extra information derived from the reduced table,and includes several columns, most of them previously explained at f) and g) paragraphs from Running NaviSE.
The reduced table includes seven columns (Rank, Gene, Locus, SE Score, # Subpeaks, Snap and Zoom out.); shown in the Fig. 21

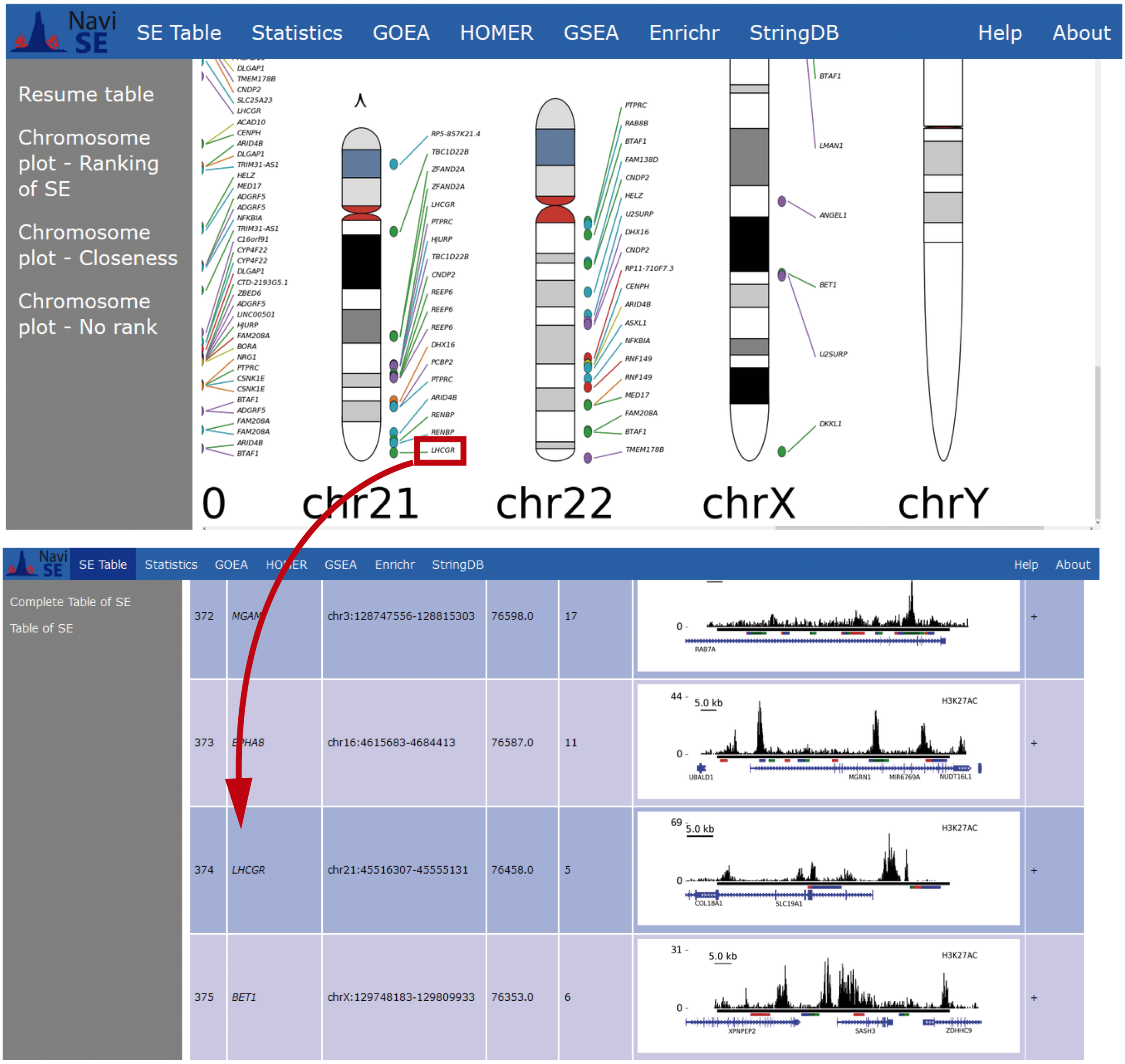

Figure 19: Main window 2.

Moreover, all those names which are included in the gene list determined by [-L] will appear in bold, for easier identification. Focusing on the two last columns, each superenhancer will contain a screenshot of the bam reads from that region. Clicking on the '+' symbol on the *Zoom out* column will redirect to the zoomed out screenshot of the superenhancer area.
Gene and *Locus* columns contain clickable links that will redirect to the Genecards website of that gene and to the UCSC Genome Browser website showing the locus region (Fig. 22).

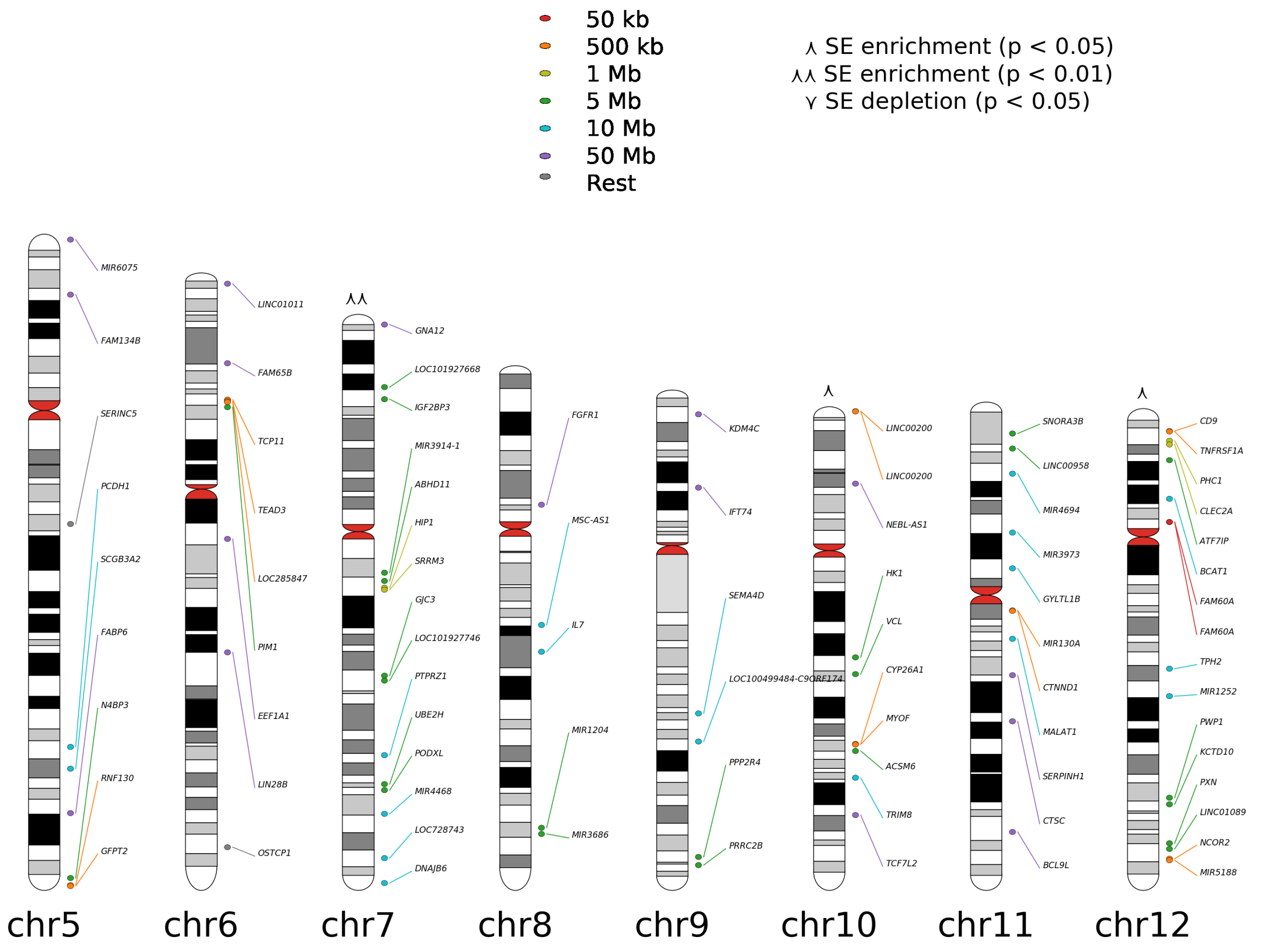

Figure 20: Chromosomal plot by closeness.

NaviSE Graphs

NaviSE implements a series of graphs which allow the user to obtain information related to the superenhancers in the sample. Those graphs are located in the *Statistics*. The sidebar contains all the accessible graphs, each of which is located in the **SUPERENHANCERS/SUPER_CELL_NAME-CONTROL/GRAPHS/** directory. If clicked on the graph, the image of the graph is displayed for easier observation (Fig. 23).
The included graphs are:

- **Ranking by SE score**: This graph could be considered as the most representative graph of the distribution of superenhancers. The superenhancer score is represented against the rank of each superenhancer, which follows a *hockey stick* distribution (Fig. 24).

  Superenhancers are painted in a darker color, while typical enhancers are painted in a lighter color. Generally, a *hockey stick* distribution in which the curve is more pronounced indicates that the resolution of the "technique/histone mark/DNA binding protein" is higher.

- **INS and OUTS**: Ins and Outs graphs contains two subgraphs. The first one shows the percentage of superenhancers or typical enhancers that contain any of the types of regions (pure,

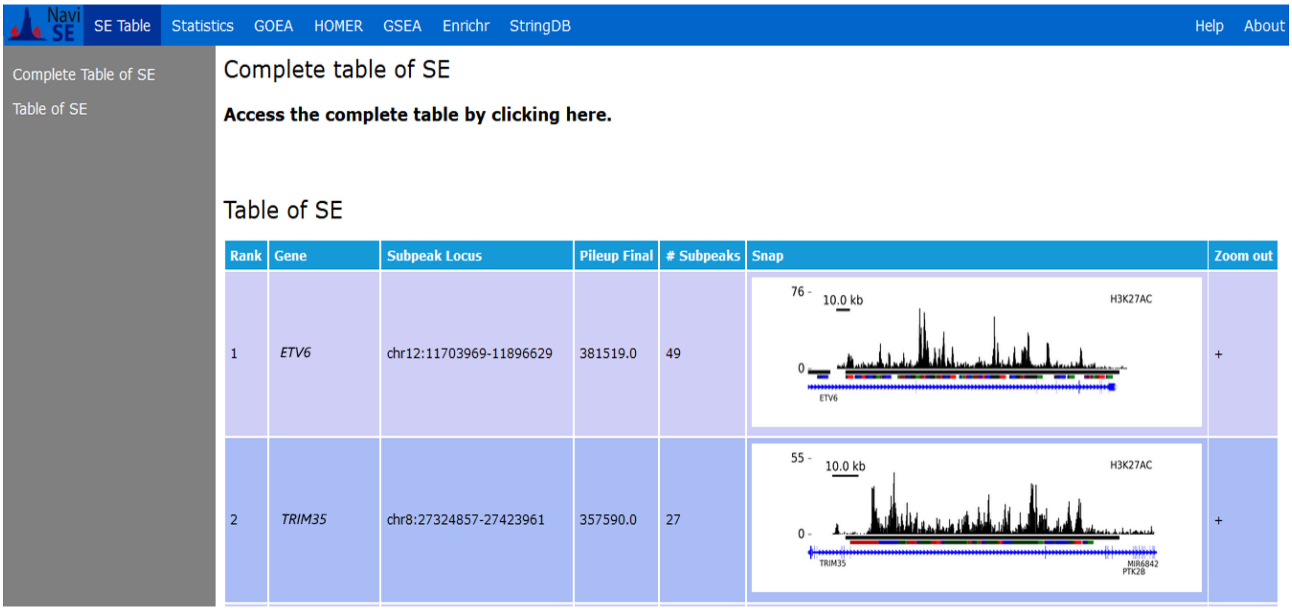

Figure 21: Superenhancer Table

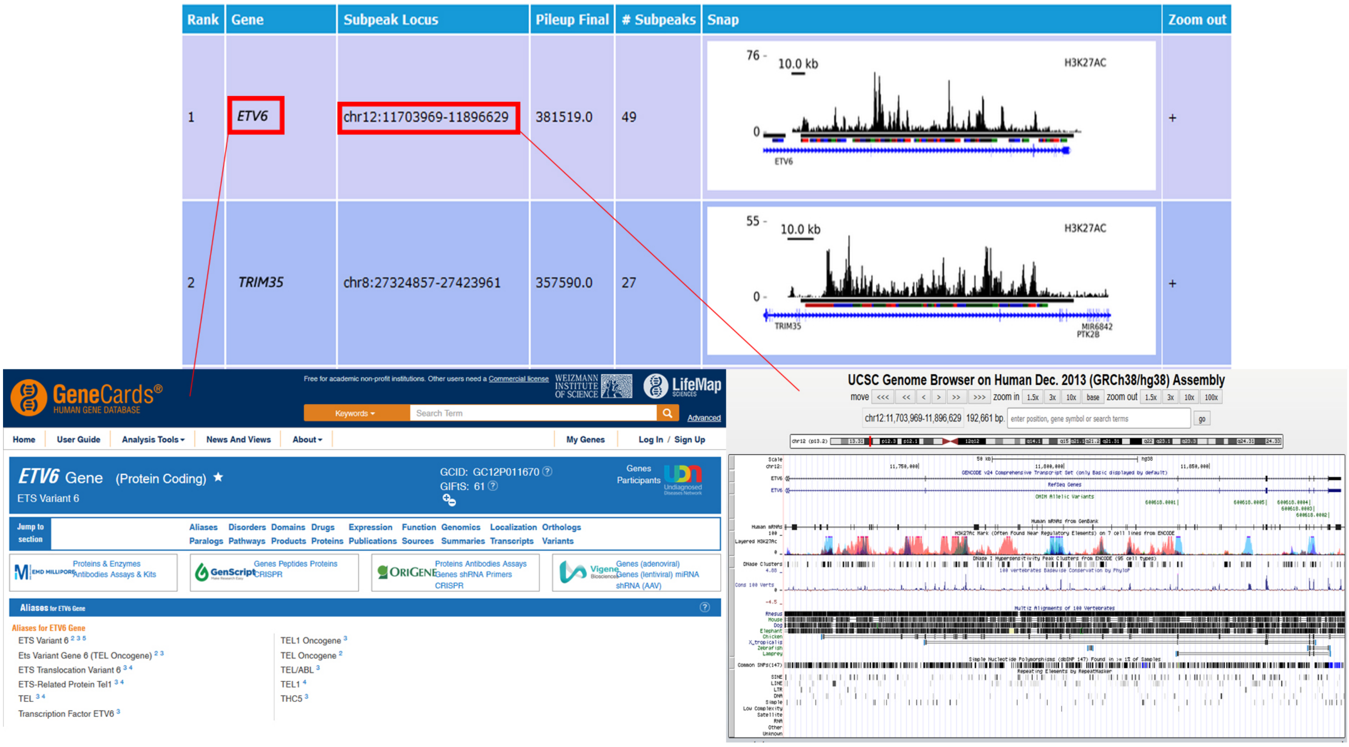

Figure 22: Superenhancer Table with links to GeneCards and UCSC Genome Browser

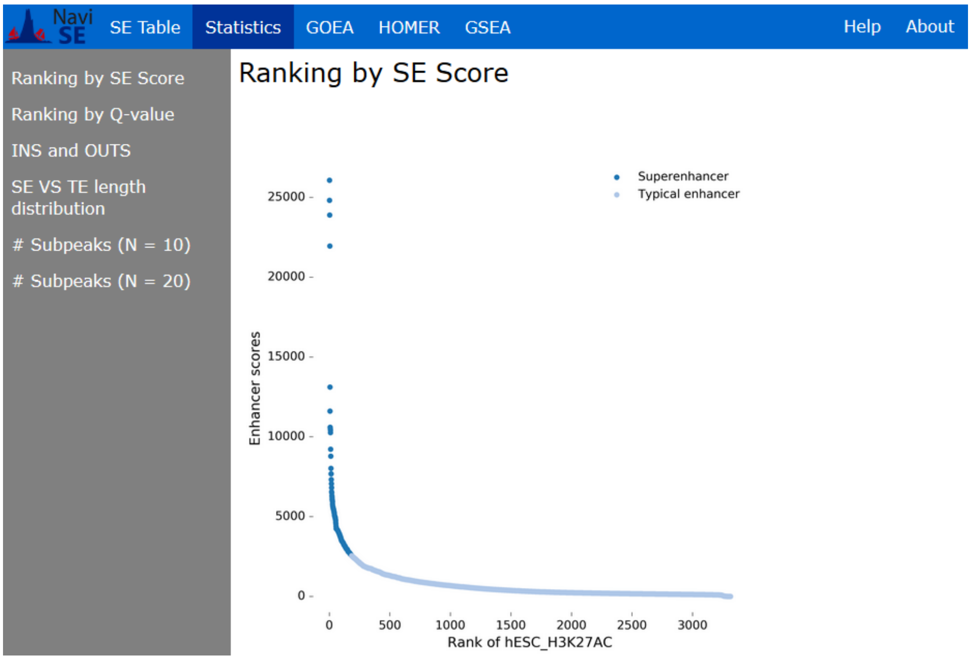

Figure 23: Window of SE Statistics.

mixed or only TSS). The second graph shows out of all types or out of mixed" types, which is the distribution of OUT subpeak in the regions. (Fig. 25)

In this example, we may observe that the number of pure superenhancers is diminished in comparison with typical enhancers, consistent with the fact that H3K27Ac is located in both TSS and enhancer regions. As for the amount of ins and outs in each superenhancer/typical enhancer, there are no statistically significant differences between both samples.

- **Length distribution**: This graph shows in a double histogram and a scatter plot the distribution of superenhancer and typical enhancer length and pileup (number of BAM reads) (Fig. 26). This graph is also developed for subpeaks from superenhancers and typical enhancers. The histogram lying on the X axis of the scatter corresponds to the length of superenhancers/typical enhancers; and the histogram on the Y-axis corresponds to the pileup.
- **Number of subpeaks**:This graph simply shows the distribution of the number of subpeaks superenhancers and typical enhancers have. (Fig. 27) Typically, typical enhancers show a zipfian distribution while superenhancers show a chi-square-like distribution, indicating that the number of subpeaks in superenhancers is clearly displaced in comparison with typical enhancers.

GOEA results

GOEA results includes all the results present at the **GENE_ONTOLOGY** directory. At first, the significant terms, that is the amount of highest-scoring positive GOEA terms (determined by the [-N] parameter) will show in a barplot representing the rank VS -log(p-value). On the other hand, a table with all the GO terms with a p-value smaller than 0.01 will be shown. GOEA discerns three different terms: biological process, molecular function and cellular component, each of which will appear as a .png file inside **GENE_ONTOLOGY** (Fig. 28).

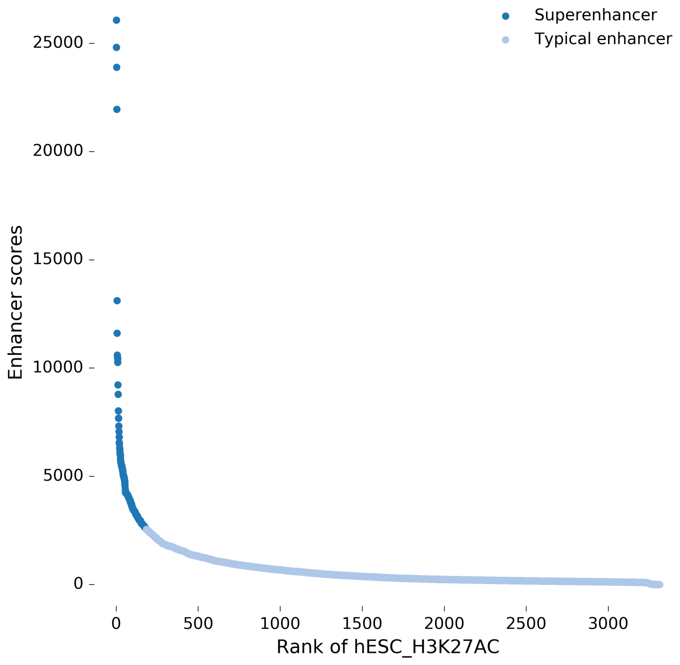

Figure 24: Rank of Superenhancers.

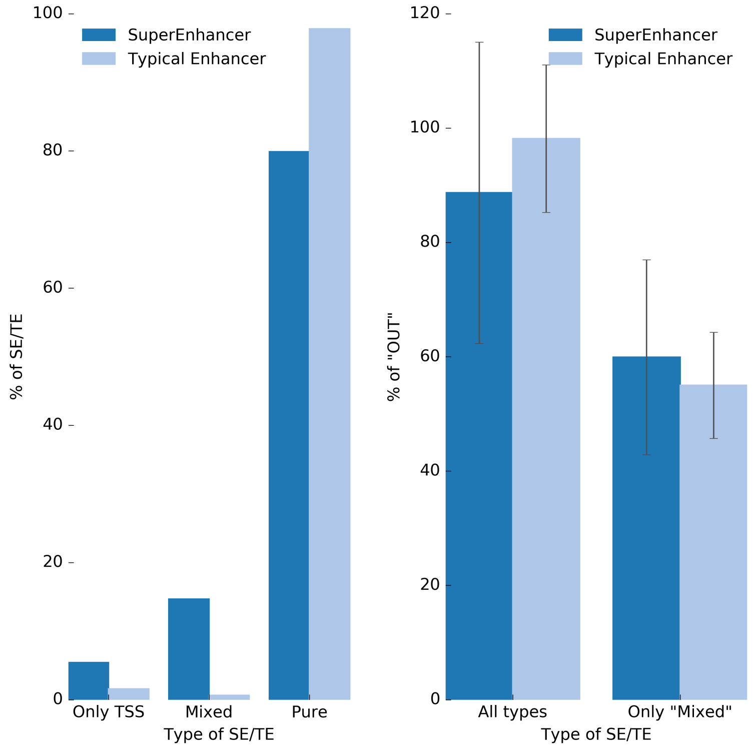

Figure 25: Graph of INS and OUTs regions.

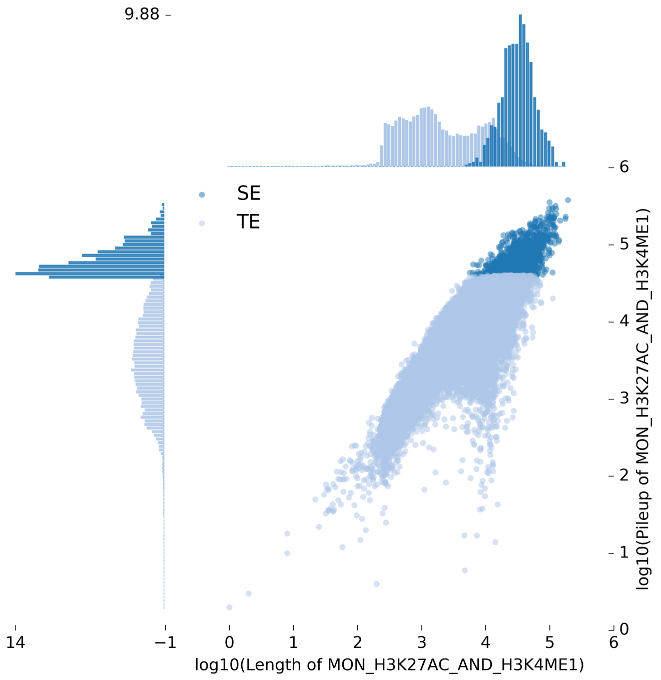

Figure 26: Graph of length/pileup distribution.

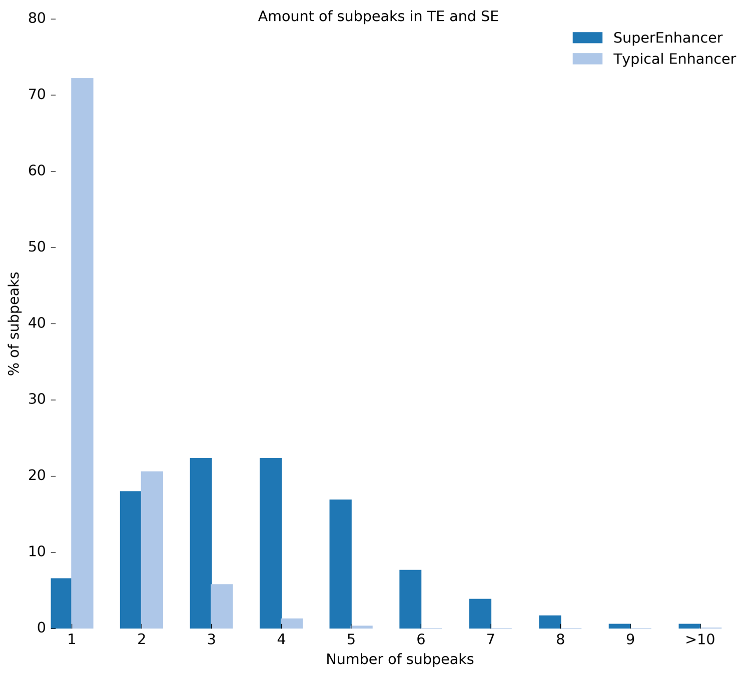

Figure 27: Graph of number of subpeaks.

These files represent a graph that links different GO terms with the positive GO terms in a way that, the higher the position in the graph, the more general the GO term is. Usually, these three graphs

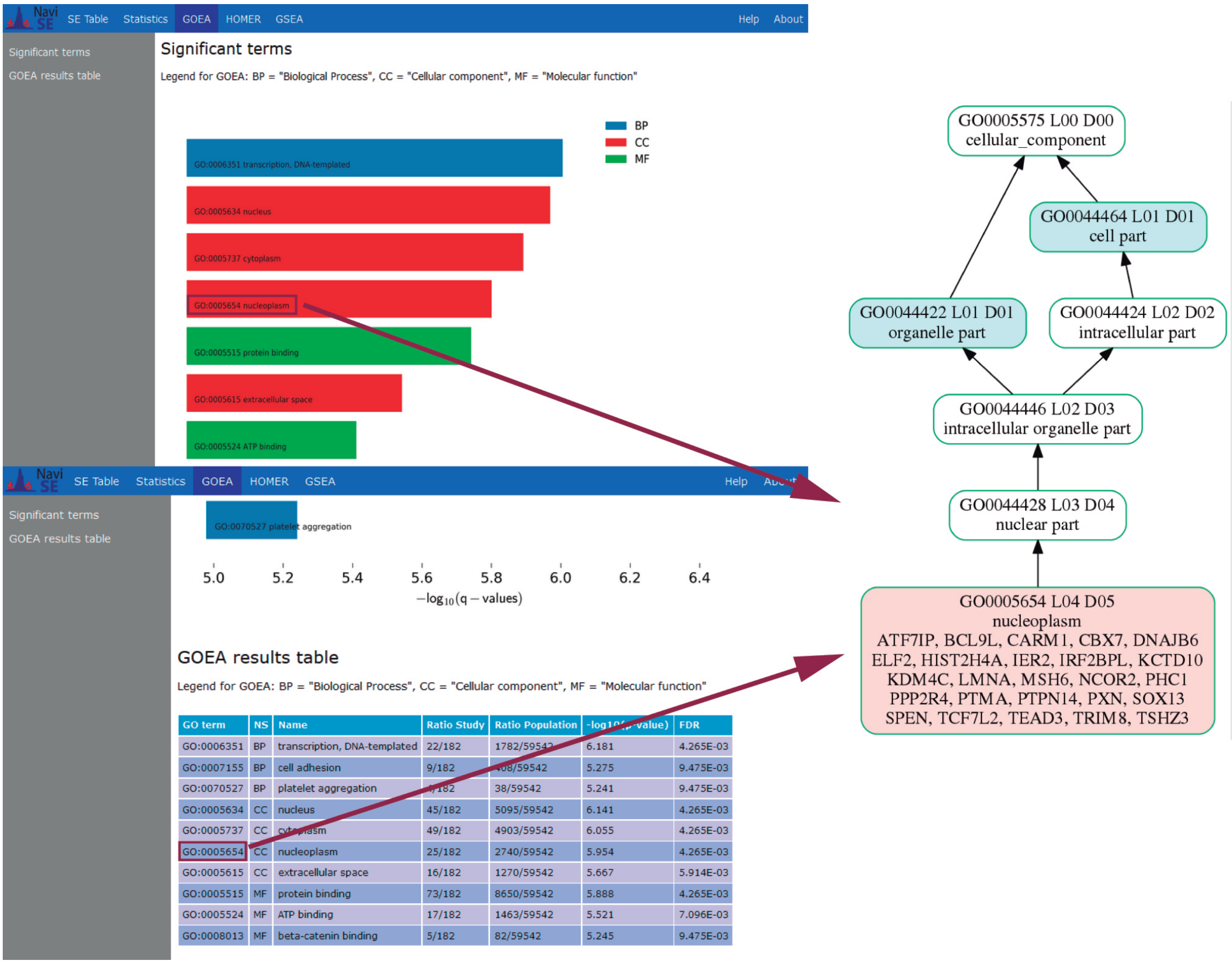

Figure 28: GOEA results.

tend to be messy due to the high amount of positive matches and, therefore, the amount of nodes and links between them.

Due to this, any term in the barplot and any cell from *GO term* column will contain a link that will redirect to the image of that individual GO term, that will contain fewer links between nodes in comparison with the general terms.

Moreover, the *GOEA results table* includes complementary columns, such as *ratio study* or *ratio population* which point out how many superenhancers out of the total superenhancers resulted positive to that GO term and how many of the genes from a pre-generated background list resulted positive in that GO term. The table shows the -log10(p-value) of this association and its correspondent False Discovery Rate value.

Note: images from GO terms may contain genes that result interesting to you and want to access to some information, like GeneCards or *SE table* data, about them. If so, it is possible to search a superenhancer in *SE table* by going to that tab in the navigation bar and pressing Ctrl + F, which will prompt a search box.

HOMER analysis

The HOMER analysis section includes two tables, one for known motifs and another one for de novo motifs. The 6rst one, HOMER known motifs contains the following distribution (Fig. 29):

- *Rank* of the motifs.
- *Motif* : LOGO of the motif
- *Name*: Name of the transcription factor / DNA binding protein that binds to that motif. It also includes a GSE number from Gene Expression Omnibus related to the experiment. Transcription factors are linked to their respective GeneCards page.
- *P-value* related to the enrichment of the sequences in suerenhancers VS typical enhancers.
- *% of Target sequences with Motif* and *% of Background sequences with Motif*, being Target the superenhancers and the Background the typical enhancers.

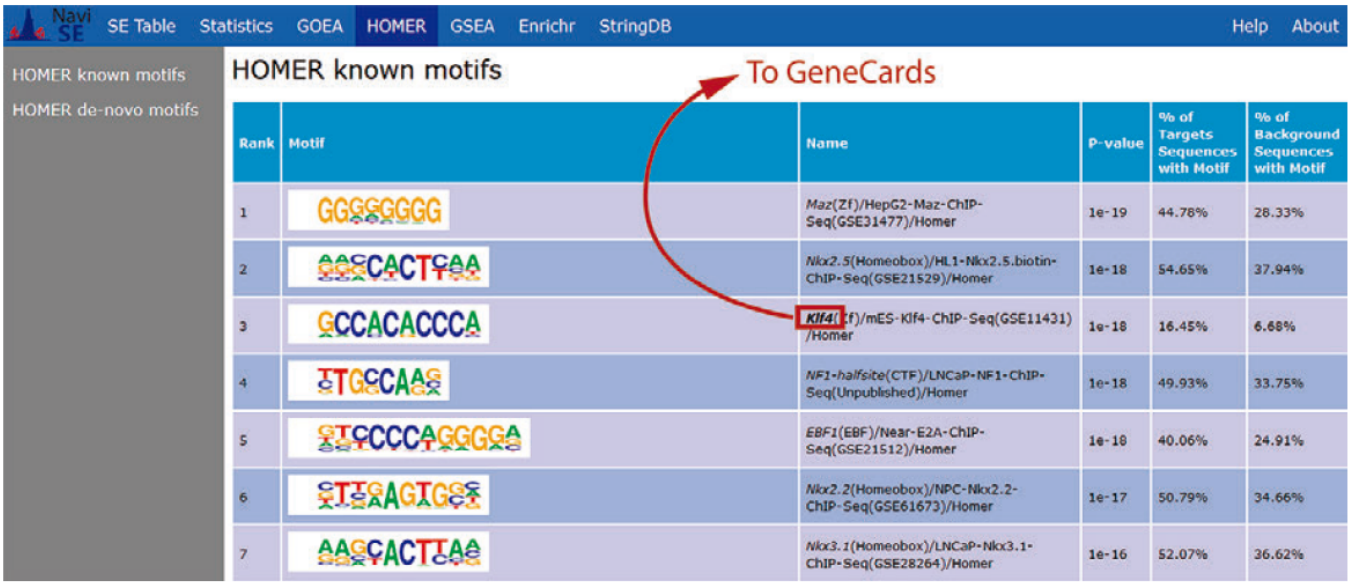

Figure 29: Table of motifs from HOMER

As for de novo motifs, the table is similar to known motifs, with some differences: (Fig. 30)

- *Rank*, *Motif*, P-*value*, *% of targets* and *% of background* are the same as in known motifs.
- *Best match*: it is a display of a known transcription factor which most closely matches the *de novo* motif. Clicking on the text leads to a second page which includes further information about the motif and other matches that HOMER assigns to this motif.

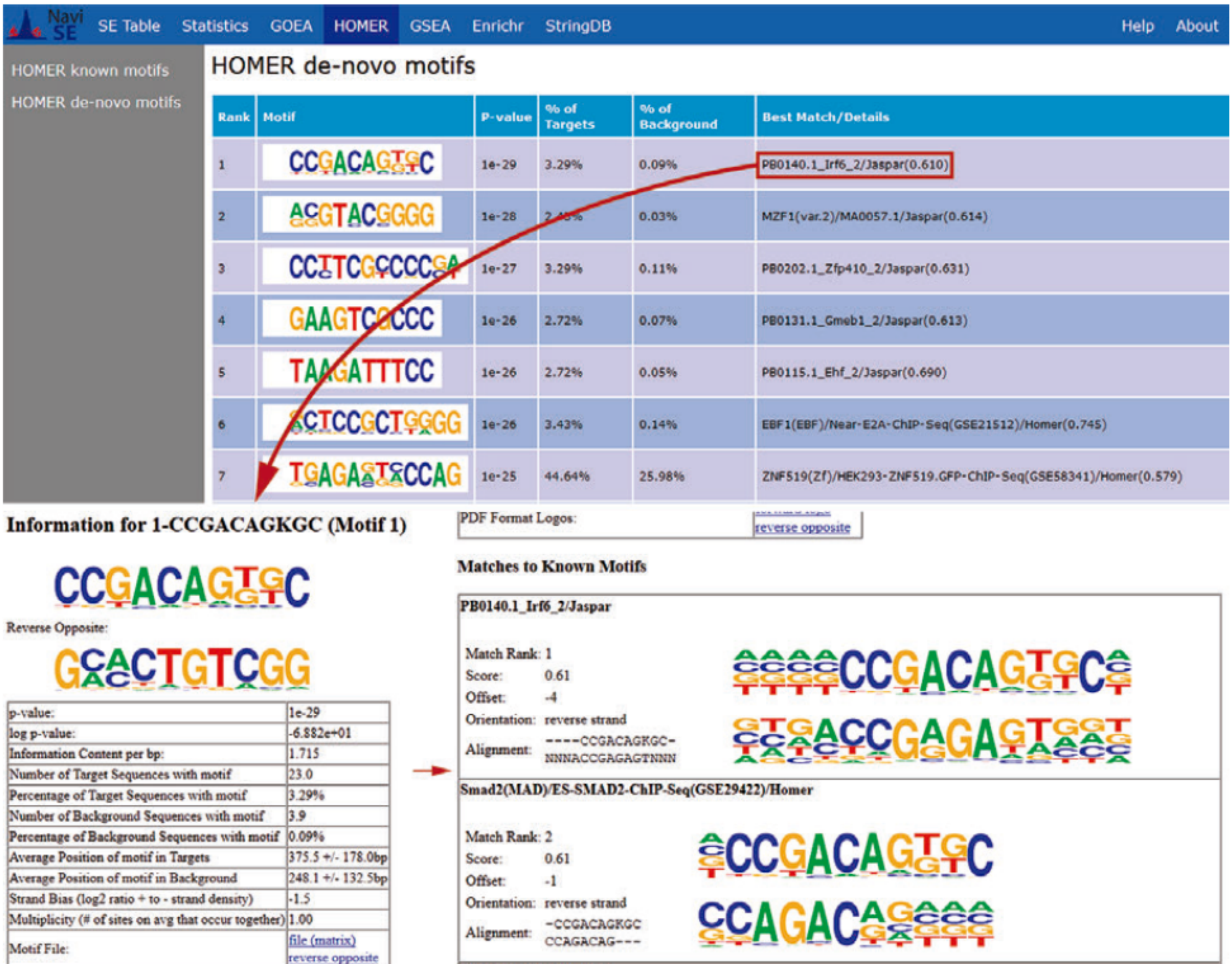

Figure 30: Table of motifs from HOMER

Warning: Analysis of motifs (almost as anything else on NaviSE report) are based on predictions. As HOMER webpage indicates, HOMER results (even more **de novo** result) must be taken **With a grain of salt**. These are orientative results and hence, discovering that the best match of any analysis is the XX transcription factor does not **directly** imply that XX is the main target of suerenhancers and plays an essential role for that cellular type in particular. Therefore, it is highly recommendable to take some time and do a thorough comparison between the multiple results NaviSE o5ers before making erroneous claims. We personally recommend taking into account the personal tips HOMER offers before launching into analyzing HOMER results

Enrichr results

As it is explained above, Enrichr comprises a number of databases related to transcription factors or genetic regulation (ENCODE/ChEA, JASPAR/TRANSFAC), cell/tissue speci6cation or metabolic pathways (KEGG, Wikipathways, Reactome).
The overall of the page (Fig. 31) contains all the figures first and the tables afterward. Clicking on each name in the barplot will lead to its corresponding term in the table. Each barplot represents the -log10(p-value) of the corresponding term. Depending on the p-value, there are three possible colors for each bar: gray, if p-value > 0.05, **light colour** if p-value < 0.05 and **dark colour** if p-value < 0.01. Bars are ordered depending on their p-value. Each result is explained below.

TRANSFAC and JASPAR PWMs

TRANSFAC/JASPAR (Fig. 32) contains information about transcription factors related to the superenhancer set in the cell/tissue. The table contains several columns: Term indicates the transcription factor associated with the superenhancer, with positive superenhancers matching to that transcription factor appearing in Genes column. Both Term and Genes contain links to GeneCards of their respective genes. Moreover, other two columns (which also appear in other tables) are Adjusted p-value and Z-score, which are intrinsic values indicating the quality of the match.

ENCODE/ChEA Transcription factors from ChIP-X

ENCODE/ChEA, similar to TRANSFAC/JASPAR, yields a list of transcription factors related to the set of superenhancers (Fig. 33). The distribution of columns is identical to TRANSFAC/JASPAR, with Term and Genes columns containing linkable items to GeneCards page of the corresponding gene.

Gene Atlas

Gene Atlas includes cell types to which there might be some relationship with the genes associated with superenhancers in the sample. Thus, the aim of this table would be to indicate to which tissue/ cell type the sample may belong. The table (Fig. 34) contains a Term column, which represents the cell type or tissue, and a Genes column with the genes corresponding to the sample that correlate to the Term. This genes are linked to their respective GeneCards page.

Wikipaths, KEGG Pathways and Reactome

Those three sections contain information about pathways (similar to GOEA or GSEA) whose genes will be present in the superenhancers of the sample. Concerning the content of the columns, they are practically the same as the ones beforehand. We may remark two of the columns: (Fig. 35) The ID

column contains a unique identi6cative item that is related to the name in Term column. This ID name is linked to its respective pathway webpage (i.e. Reactome, Wikipathways or KEGG pathways). The superenhancer genes related to that Term are located in Genes column, which are linked to their respective GeneCards page.

StringDB results

String section contains the protein-protein interaction networks from superenhancers (Fig. 36). There are different *confidence* values determined by how well documented the interaction is, amongst other factors. The current con6dence values are 0.4, 0.7, 0.9, 0.95 and 0.99. This range of values allows users to choose the network that best 6ts their needs, as networks with low *confidence* levels will be overcrowded whereas networks with high *confidence* levels may contain few elements. The links between nodes (genes) are colored in a color code, and the nodes vary in size, as shown in the legend below (Fig. 37).

GSEA results

The *GSEA* section (Fig. 38) includes all the positive analysis for the selected thresholds and signatures. Each signature contains several graphs ordered alphabetically depicting the GSEA curve of the analysis. Generally, the more pronounced and hip-like the curve, the better.

Each graph contains a curve, and below it there is a box with black lines that depict the matches of superenhancer + typical enhancers to the genes corresponding to the gene set from the signature. Below this box there is a graph which shows the value of each position (in this case, the superenhancer score).

Clicking on a graph leads to its corresponding element on a table below, which contains several columns. Focusing on the *SE Genes (Rank)* column, each cell contains genes corresponding to those positive matches, which lead to their respective GeneCards site, and inside parenthesis, there is a number that represents the rank of this gene in the superenhancer list. Clicking on this value will lead to the respective row from *SE Table* section.

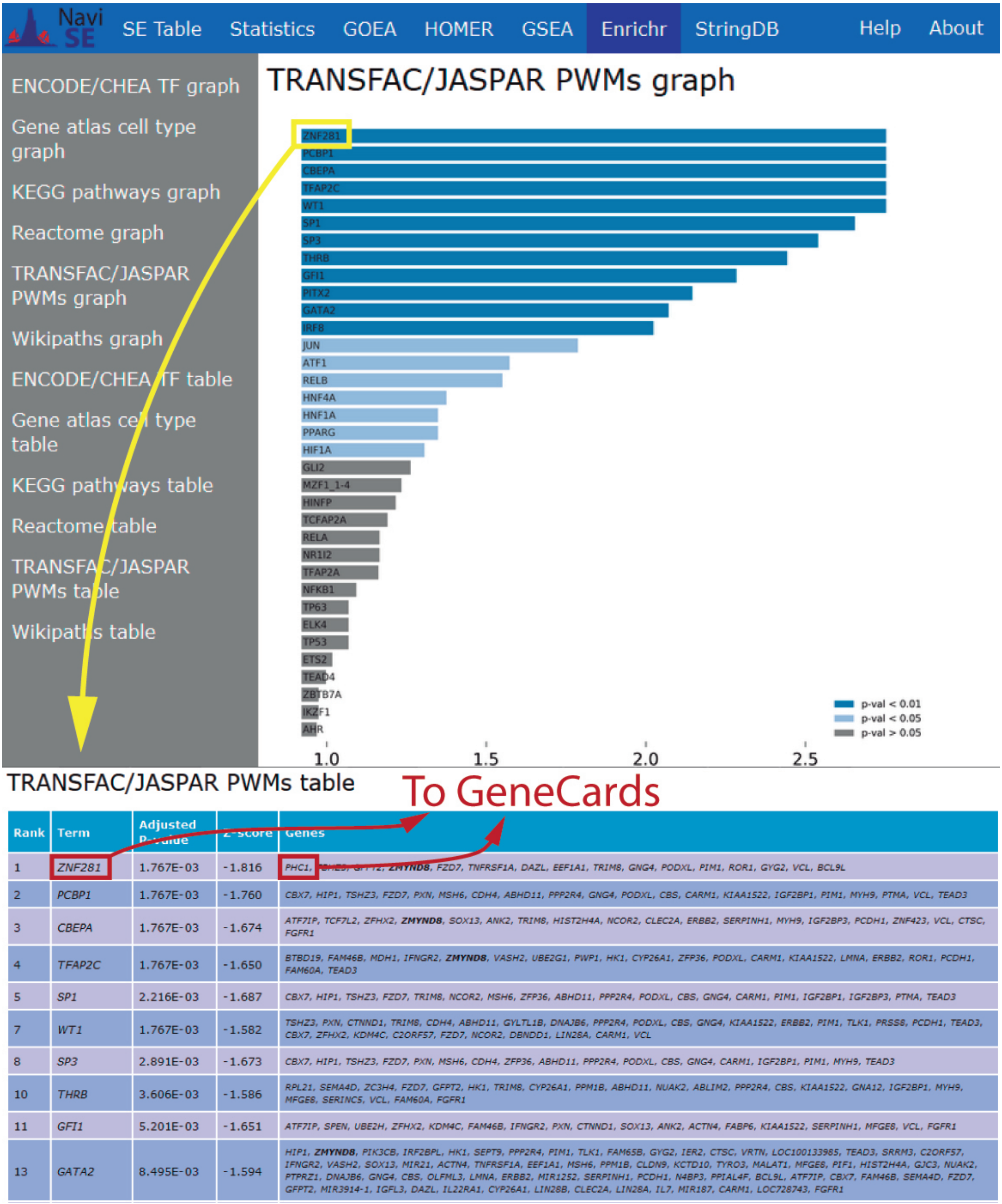

Rank	Term	Adjusted P-value	Z-score	Genes
1	*ZNF281*	1.767E-03	-1.816	*PHC1, [illegible], **ZMYND8**, FZD7, TNFRSF1A, DAZL, EEF1A1, TRIM8, GNG4, PODXL, PIM1, ROR1, GYG2, VCL, BCL9L*
2	*PCBP1*	1.767E-03	-1.760	*CBX7, HIP1, TSHZ3, FZD7, PXN, MSH6, CDH4, ABHD11, PPP2R4, GNG4, PODXL, CBS, CARM1, KIAA1522, IGF2BP1, PIM1, MYH9, PTMA, VCL, TEAD3*
3	*CBEPA*	1.767E-03	-1.674	*ATF7IP, TCF7L2, ZFHX2, **ZMYND8**, SOX13, ANK2, TRIM8, HIST2H4A, NCOR2, CLEC2A, ERBB2, SERPINH1, MYH9, IGF2BP3, PCDH1, ZNF423, VCL, CTSC, FGFR1*
4	*TFAP2C*	1.767E-03	-1.650	*BTBD19, FAM46B, MDH1, IFNGR2, **ZMYND8**, VASH2, UBE2G1, PWP1, HK1, CYP26A1, ZFP36, PODXL, CARM1, KIAA1522, LMNA, ERBB2, ROR1, PCDH1, FAM60A, TEAD3*
5	*SP1*	2.216E-03	-1.687	*CBX7, HIP1, TSHZ3, FZD7, TRIM8, NCOR2, MSH6, ZFP36, ABHD11, PPP2R4, PODXL, CBS, GNG4, CARM1, PIM1, IGF2BP1, IGF2BP3, PTMA, TEAD3*
7	*WT1*	1.767E-03	-1.582	*TSHZ3, PXN, CTNND1, TRIM8, CDH4, ABHD11, GYLTL1B, DNAJB6, PPP2R4, PODXL, CBS, GNG4, KIAA1522, ERBB2, PIM1, TLK1, PRSS8, PCDH1, TEAD3, CBX7, ZFHX2, KDM4C, C2ORF57, FZD7, NCOR2, DBNDD1, LIN28A, CARM1, VCL*
8	*SP3*	2.891E-03	-1.673	*CBX7, HIP1, TSHZ3, FZD7, PXN, MSH6, CDH4, ZFP36, ABHD11, PPP2R4, PODXL, CBS, GNG4, CARM1, IGF2BP1, PIM1, MYH9, TEAD3*
10	*THRB*	3.606E-03	-1.586	*RPL21, SEMA4D, ZC3H4, FZD7, GFPT2, HK1, TRIM8, CYP26A1, PPM1B, ABHD11, NUAK2, ABLIM2, PPP2R4, CBS, KIAA1522, GNA12, IGF2BP1, MYH9, MFGE8, SERINC5, VCL, FAM60A, FGFR1*
11	*GFI1*	5.201E-03	-1.651	*ATF7IP, SPEN, UBE2H, ZFHX2, KDM4C, FAM46B, IFNGR2, PXN, CTNND1, SOX13, ANK2, ACTN4, FABP6, KIAA1522, SERPINH1, MFGE8, VCL, FGFR1*
13	*GATA2*	8.495E-03	-1.594	*HIP1, **ZMYND8**, PIK3CB, IRF2BPL, HK1, SEPT9, PPP2R4, PIM1, TLK1, FAM65B, GYG2, IER2, CTSC, VRTN, LOC100133985, TEAD3, SRRM3, C2ORF57, IFNGR2, VASH2, SOX13, MIR21, ACTN4, TNFRSF1A, EEF1A1, MSH6, PPM1B, CLDN9, KCTD10, TYRO3, MALAT1, MFGE8, PIF1, HIST2H4A, GJC3, NUAK2, PTPRZ1, DNAJB6, GNG4, CBS, OLFML3, LMNA, ERBB2, MIR1252, SERPINH1, PCDH1, N4BP3, PPIAL4F, BCL9L, ATF7IP, CBX7, FAM46B, SEMA4D, FZD7, GFPT2, MIR3914-1, IGFL3, DAZL, IL22RA1, CYP26A1, LIN28B, CLEC2A, LIN28A, IL7, MIR187, CARM1, LOC728743, FGFR1*

Figure 31: Enrichr overall window.

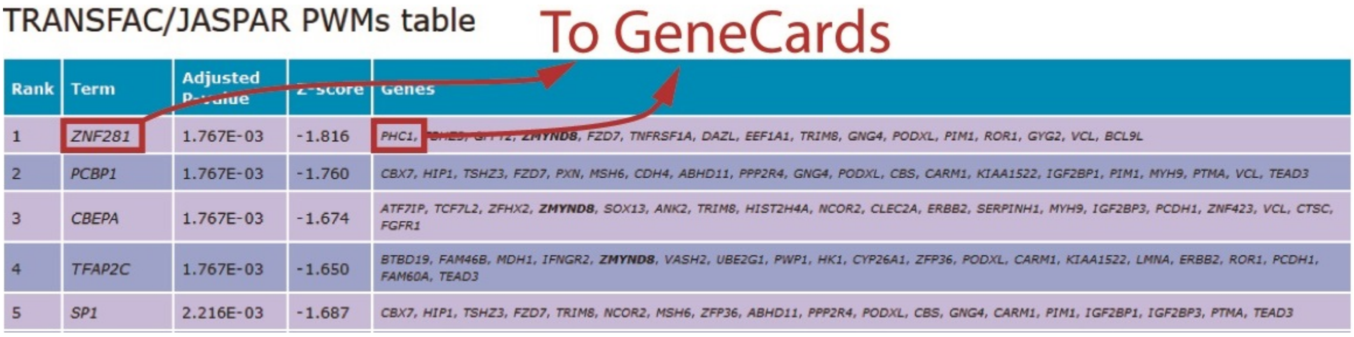

Rank	Term	Adjusted P-value	Z-score	Genes
1	*ZNF281*	1.767E-03	-1.816	*PHC1, [illegible], **ZMYND8**, FZD7, TNFRSF1A, DAZL, EEF1A1, TRIM8, GNG4, PODXL, PIM1, ROR1, GYG2, VCL, BCL9L*
2	*PCBP1*	1.767E-03	-1.760	*CBX7, HIP1, TSHZ3, FZD7, PXN, MSH6, CDH4, ABHD11, PPP2R4, GNG4, PODXL, CBS, CARM1, KIAA1522, IGF2BP1, PIM1, MYH9, PTMA, VCL, TEAD3*
3	*CBEPA*	1.767E-03	-1.674	*ATF7IP, TCF7L2, ZFHX2, **ZMYND8**, SOX13, ANK2, TRIM8, HIST2H4A, NCOR2, CLEC2A, ERBB2, SERPINH1, MYH9, IGF2BP3, PCDH1, ZNF423, VCL, CTSC, FGFR1*
4	*TFAP2C*	1.767E-03	-1.650	*BTBD19, FAM46B, MDH1, IFNGR2, **ZMYND8**, VASH2, UBE2G1, PWP1, HK1, CYP26A1, ZFP36, PODXL, CARM1, KIAA1522, LMNA, ERBB2, ROR1, PCDH1, FAM60A, TEAD3*
5	*SP1*	2.216E-03	-1.687	*CBX7, HIP1, TSHZ3, FZD7, TRIM8, NCOR2, MSH6, ZFP36, ABHD11, PPP2R4, PODXL, CBS, GNG4, CARM1, PIM1, IGF2BP1, IGF2BP3, PTMA, TEAD3*

Figure 32: Transfac/Jaspar PWMs table.

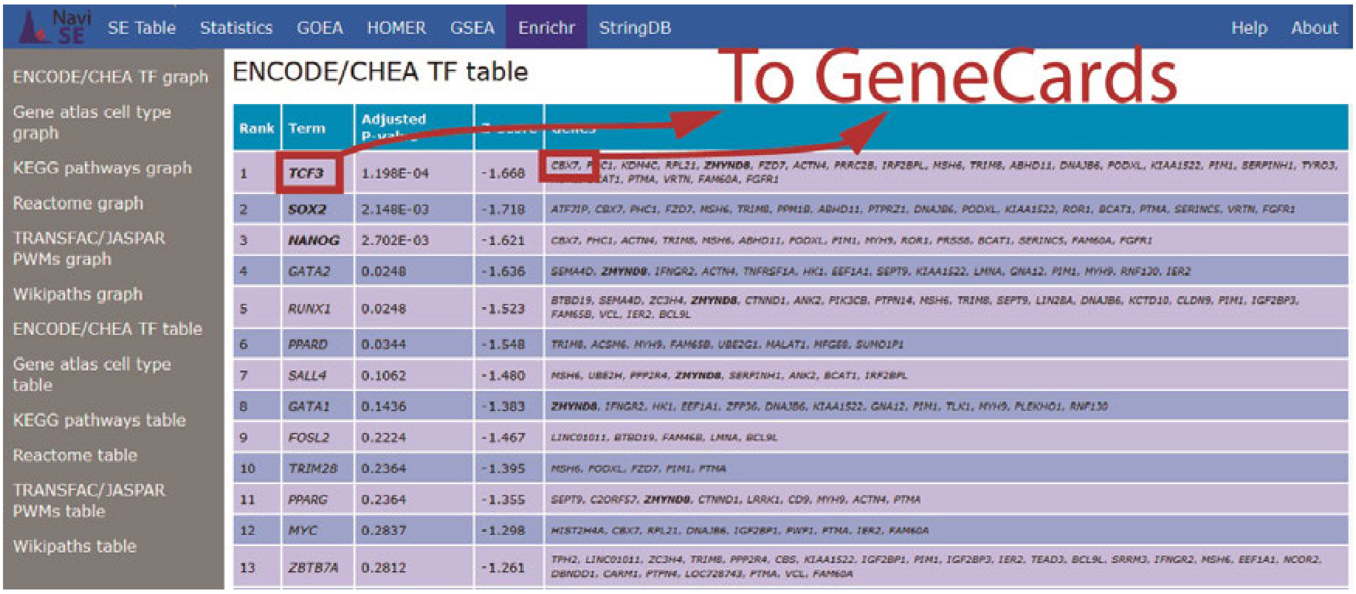

Rank	Term	Adjusted P-value	Z-score	Genes
1	***TCF3***	1.198E-04	-1.668	*CBX7, [illegible]C1, KDM4C, RPL21, **ZMYND8**, FZD7, ACTN4, PRRC2B, IRF2BPL, MSH6, TRIM8, ABHD11, DNAJB6, PODXL, KIAA1522, PIM1, SERPINH1, TYRO3, [illegible]AT1, PTMA, VRTN, FAM60A, FGFR1*
2	***SOX2***	2.148E-03	-1.718	*ATF7IP, CBX7, PHC1, FZD7, MSH6, TRIM8, PPM1B, ABHD11, PTPRZ1, DNAJB6, PODXL, KIAA1522, ROR1, BCAT1, PTMA, SERINC5, VRTN, FGFR1*
3	***NANOG***	2.702E-03	-1.621	*CBX7, PHC1, ACTN4, TRIM8, MSH6, ABHD11, PODXL, PIM1, MYH9, ROR1, PRSS8, BCAT1, SERINC5, FAM60A, FGFR1*
4	*GATA2*	0.0248	-1.636	*SEMA4D, **ZMYND8**, IFNGR2, ACTN4, TNFRSF1A, HK1, EEF1A1, SEPT9, KIAA1522, LMNA, GNA12, PIM1, MYH9, RNF130, IER2*
5	*RUNX1*	0.0248	-1.523	*BTBD19, SEMA4D, ZC3H4, **ZMYND8**, CTNND1, ANK2, PIK3CB, PTPN14, MSH6, TRIM8, SEPT9, LIN28A, DNAJB6, KCTD10, CLDN9, PIM1, IGF2BP3, FAM65B, VCL, IER2, BCL9L*
6	*PPARD*	0.0344	-1.548	*TRIM8, ACSM6, MYH9, FAM65B, UBE2G1, MALAT1, MFGE8, SUMO1P1*
7	*SALL4*	0.1062	-1.480	*MSH6, UBE2H, PPP2R4, **ZMYND8**, SERPINH1, ANK2, BCAT1, IRF2BPL*
8	*GATA1*	0.1436	-1.383	***ZMYND8**, IFNGR2, HK1, EEF1A1, ZFP36, DNAJB6, KIAA1522, GNA12, PIM1, TLK1, MYH9, PLEKHO1, RNF130*
9	*FOSL2*	0.2224	-1.467	*LINC01011, BTBD19, FAM46B, LMNA, BCL9L*
10	*TRIM28*	0.2364	-1.395	*MSH6, PODXL, FZD7, PIM1, PTMA*
11	*PPARG*	0.2364	-1.355	*SEPT9, C2ORF57, **ZMYND8**, CTNND1, LRRK1, CD9, MYH9, ACTN4, PTMA*
12	*MYC*	0.2837	-1.298	*HIST2H4A, CBX7, RPL21, DNAJB6, IGF2BP1, PWP1, PTMA, IER2, FAM60A*
13	*ZBTB7A*	0.2812	-1.261	*TPH2, LINC01011, ZC3H4, TRIM8, PPP2R4, CBS, KIAA1522, IGF2BP1, PIM1, IGF2BP3, IER2, TEAD3, BCL9L, SRRM3, IFNGR2, MSH6, EEF1A1, NCOR2, DBNDD1, CARM1, PTPN4, LOC728743, PTMA, VCL, FAM60A*

Figure 33: ENCODE/CHEA TF tabl.e

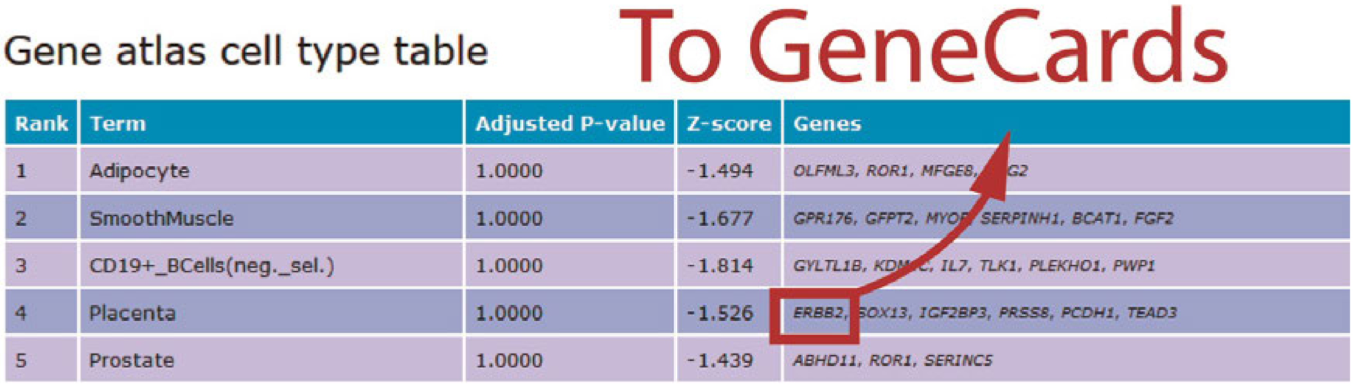

Rank	Term	Adjusted P-value	Z-score	Genes
1	Adipocyte	1.0000	-1.494	*OLFML3, ROR1, MFGE8, [illegible]G2*
2	SmoothMuscle	1.0000	-1.677	*GPR176, GFPT2, MYO[illegible] SERPINH1, BCAT1, FGF2*
3	CD19+_BCells(neg._sel.)	1.0000	-1.814	*GYLTL1B, KDM[illegible], IL7, TLK1, PLEKHO1, PWP1*
4	Placenta	1.0000	-1.526	*ERBB2, SOX13, IGF2BP3, PRSS8, PCDH1, TEAD3*
5	Prostate	1.0000	-1.439	*ABHD11, ROR1, SERINC5*

Figure 34: Gene atlas cell type table.

As for the rest of the columns, which appear in more detail here, **ES** and **NES** are the Enrichment Score and the Normalised Enrichment Score. ES reflects the degree to which a gene set is over-represented at the top or bottom of a ranked list of genes. The ES is the maximum deviation from

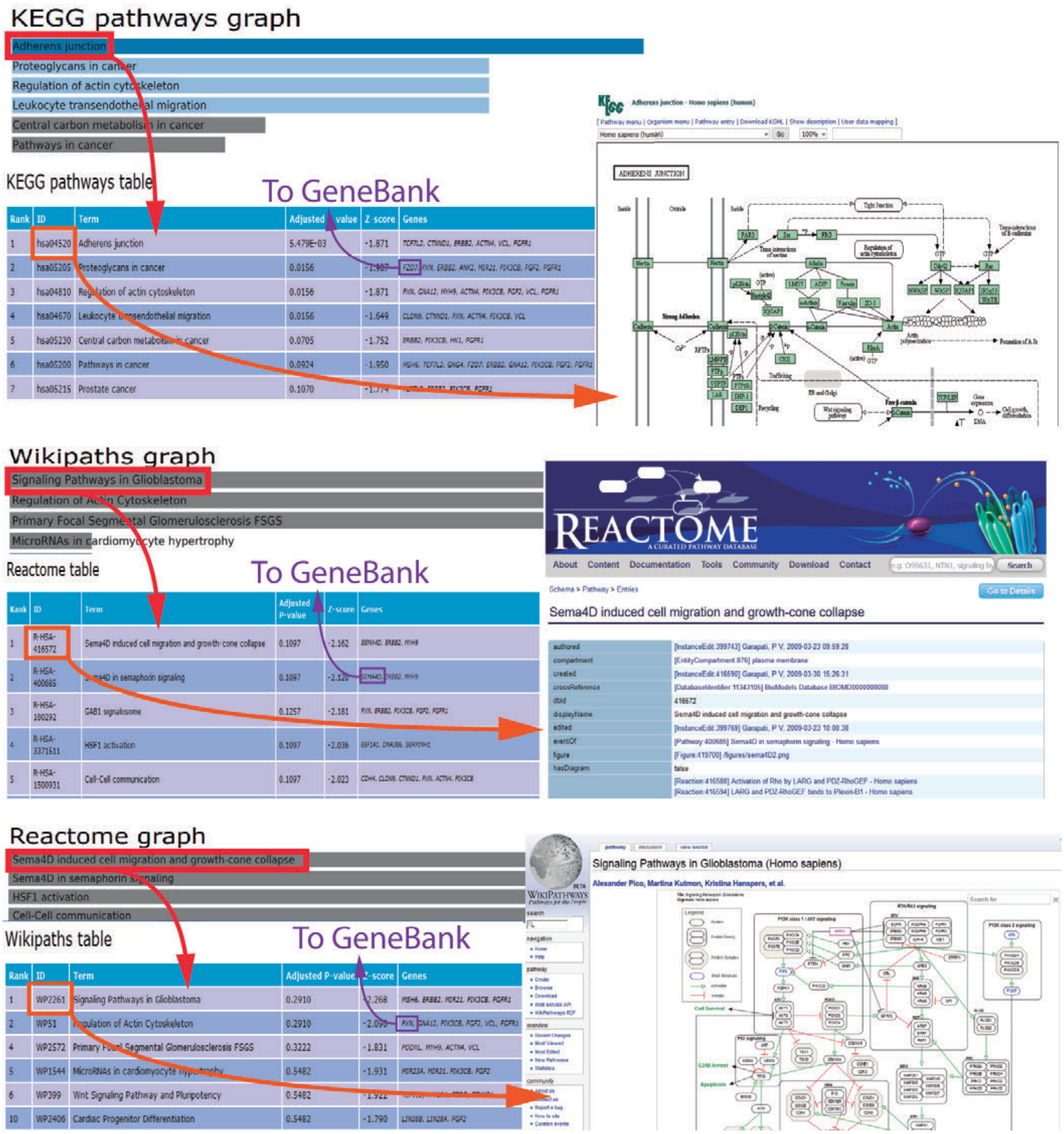

Figure 35: Metabolism pathways tables and main targets.

zero encountered in walking the list. A positive ES indicates gene set enrichment at the top of the ranked list; a negative ES indicates gene set enrichment at the bottom of the ranked list. In our case, we are not going to find negative ES. The normalized enrichment score (NES) is the primary statistic for examining gene set enrichment results. By normalizing the enrichment score, GSEA accounts for differences in gene set size and in correlations between gene sets and the expression dataset;

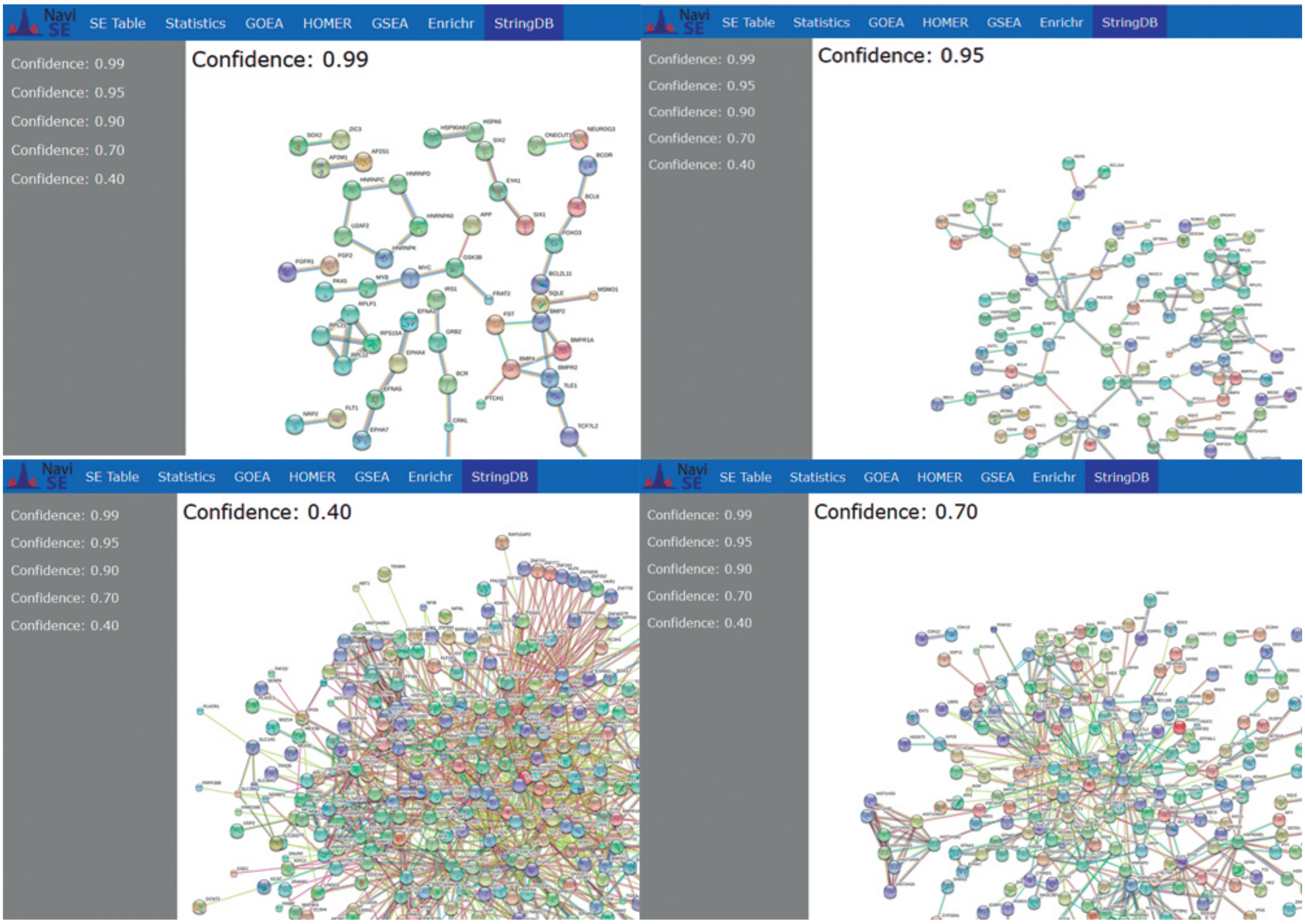

Figure 36: String window.

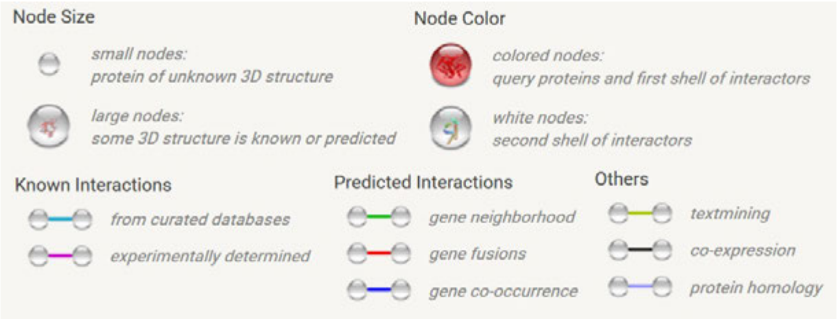

Figure 37: String legend.

therefore, the normalized enrichment scores (NES) can be used to compare analysis results across gene sets.

The **FDR** is the estimated probability that a gene set with a given NES represents a false positive finding. Thus, the smaller the FDR the better. The **nominal p value** estimates the statistical significance of the enrichment score for a single gene set. Finally, the **−log10(Ratio p-value)** corresponds to the hypergeometric test between the number of matches between SE and TE, and the number of

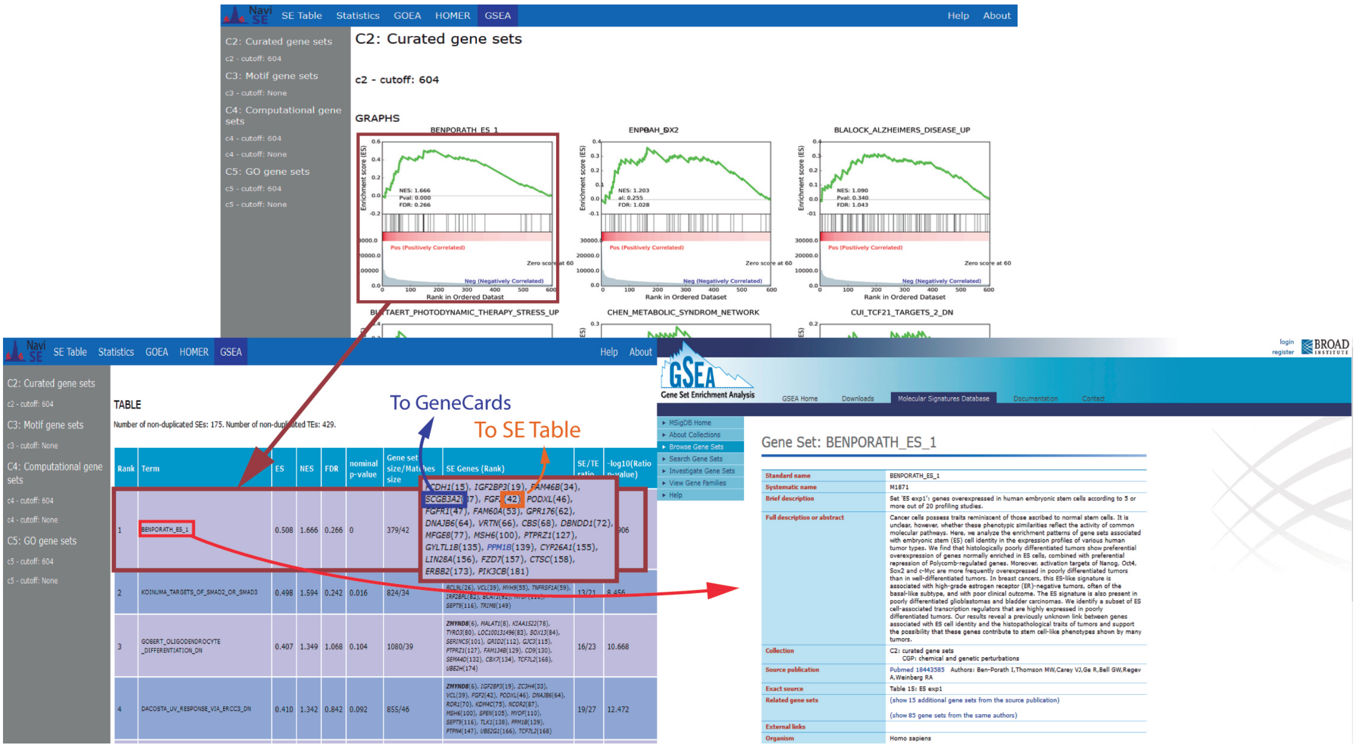

Figure 38: GSEA window.

SE and TE (with or without matched) that our sample contains. This p-value should be indicative of how enriched in SE matches against TE matches the sample is.

Thus, the user should be able to discern which graphs are really representative based in FDR, nominal p-value, distribution of the curve and NES, from those graphs which also appear but are not fully reliable or statistically significant.
